# Diversity and Antimicrobial Activity of Vietnamese Sponge-Associated Bacteria

**DOI:** 10.3390/md19070353

**Published:** 2021-06-22

**Authors:** Ton That Huu Dat, Nguyen Thi Kim Cuc, Pham Viet Cuong, Hauke Smidt, Detmer Sipkema

**Affiliations:** 1 Mientrung Institute for Scientific Research, Vietnam Academy of Science and Technology, 321 Huynh Thuc Khang, Hue City, Thua Thien Hue 531600, Vietnam; kimcuc@imbc.vast.vn (N.T.K.C.); pvcuong@misr.vast.vn (P.V.C.); 2 Laboratory of Microbiology, Wageningen University & Research, Stippeneng 4, 6708 WE Wageningen, The Netherlands; hauke.smidt@wur.nl

**Keywords:** antimicrobial activity, cultivable bacteria, secondary metabolites, sponge-associated bacteria

## Abstract

This study aimed to assess the diversity and antimicrobial activity of cultivable bacteria associated with Vietnamese sponges. In total, 460 bacterial isolates were obtained from 18 marine sponges. Of these, 58.3% belonged to *Proteobacteria*, 16.5% to *Actinobacteria*, 18.0% to *Firmicutes*, and 7.2% to *Bacteroidetes*. At the genus level, isolated strains belonged to 55 genera, of which several genera, such as *Bacillus*, *Pseudovibrio*, *Ruegeria*, *Vibrio*, and *Streptomyces*, were the most predominant. Culture media influenced the cultivable bacterial composition, whereas, from different sponge species, similar cultivable bacteria were recovered. Interestingly, there was little overlap of bacterial composition associated with sponges when the taxa isolated were compared to cultivation-independent data. Subsequent antimicrobial assays showed that 90 isolated strains exhibited antimicrobial activity against at least one of seven indicator microorganisms. From the culture broth of the isolated strain with the strongest activity (*Bacillus* sp. M1_CRV_171), four secondary metabolites were isolated and identified, including cyclo(L-Pro-L-Tyr) (**1**), macrolactin A (**2**), macrolactin H (**3**), and 15,17-epoxy-16-hydroxy macrolactin A (**4**). Of these, compounds **2**-**4** exhibited antimicrobial activity against a broad spectrum of reference microorganisms.

## 1. Introduction

Antimicrobial resistance decreases the possibilities for prevention and treatment of infectious diseases caused by viruses, bacteria, parasites, and fungi. Factors listed as causes for the rising prevalence of antibiotic resistance include over-prescription of antibiotics both in hospitals and agriculture, poor infection control in hospitals and clinics, lack of hygiene, and poor sanitation [1,2,3,4,5]. Thus, antibiotic resistance has considerable social and economic impact by the increase of morbidity and mortality of infectious diseases. Currently, it is estimated that approximately 700,000 people die every year from drug-resistant infections. By 2050, antibiotic resistance may attribute to 10 million deaths worldwide each year and trigger an economic loss of up to 100 trillion US dollars [6]. While antibiotic resistance increases, only a limited number of new antibiotics have been discovered and approved for medical treatment [7]. Therefore, increasing efforts towards discovery and exploitation of novel antimicrobial compounds are urgently needed.

Modifications of existing drugs are often not effective enough to overcome the mutation rate of microbial pathogens and do not lead to the introduction of new classes of antimicrobial compounds [8]. In addition, chemical synthesis and semi-synthesis approaches of new antimicrobial compounds and their analogues are hindered by the complexity of the molecules and low yield [9,10]. Therefore, the discovery of novel antimicrobial compounds from the biosphere remains an important avenue for finding our future antibiotics.

The terrestrial environment has been the main source of new antibiotics in recent decades. However, re-discovery of known compounds has limited the development of new drugs from the terrestrial environment for treating infectious diseases [7]. The marine environment encompasses several of the richest ecosystems on Earth, with an extreme diversity of life forms. However, its bioactive compounds have been largely unexplored [11,12,13]. Of marine organisms, sponges (phylum Porifera) are considered the most promising source of bioactive natural products and contribute about 30% of the known marine natural products [14,15]. Furthermore, sponge-derived compounds exhibit a wide spectrum of biological properties, including antimicrobial, anticancer, anti-inflammatory, immunosuppressive, and neurosuppressive activity [14,15,16,17]. However, the low concentration of sponge-derived compounds in their tissues has led to issues with supply and halted preclinical and clinical studies of many promising therapeutic drug candidates [18].

To date, of many sponge-derived secondary metabolites, the true origin is unknown. The secondary metabolites could be produced by the sponges, by their microbial symbionts, or by the cooperation between sponges and symbionts [19]. Interestingly, several studies have highlighted that many bioactive compounds from sponges might be of bacterial origin due to the structural similarity of the molecules to compounds found in terrestrial microorganisms [20,21,22]. For example, it is now known that polybrominated biphenyl ether antibiotics isolated from the sponge *Dysidea herbacea* are actually produced by the endosymbiotic cyanobacterium *Oscillatoria spongeliae* [23]. The antifungal peptide theopalauamide isolated from the marine sponge *Theonella swinhoei* has been found to be contained in a δ-proteobacterial symbiont [24]. Furthermore, recent studies have confirmed that sponge-associated microorganisms are a valuable source of antimicrobial compounds with potent biological activity and diverse structural features, which make these microbial communities promising sources of novel antimicrobials [22,25,26,27]. Thus, obtaining the sponge-associated bacterial producers of these compounds as pure cultures presents a way to overcome the supply issue.

A previous study based on cultivation-independent methods showed a high diversity of the prokaryotic communities associated with Vietnamese sponges [28]. To explore the antimicrobial activity from sponge-associated bacteria, in this study, we aimed to isolate bacteria and screen the antimicrobial activity from 18 Vietnamese sponge species. The strain (*Bacillus* sp. M1_CRV_171) with the highest antimicrobial activity was then subjected to isolation and purification of antimicrobial compounds.

## 2. Results

### 2.1. Diversity of Sponge-Associated Cultivable Bacteria

**Figure 1:**
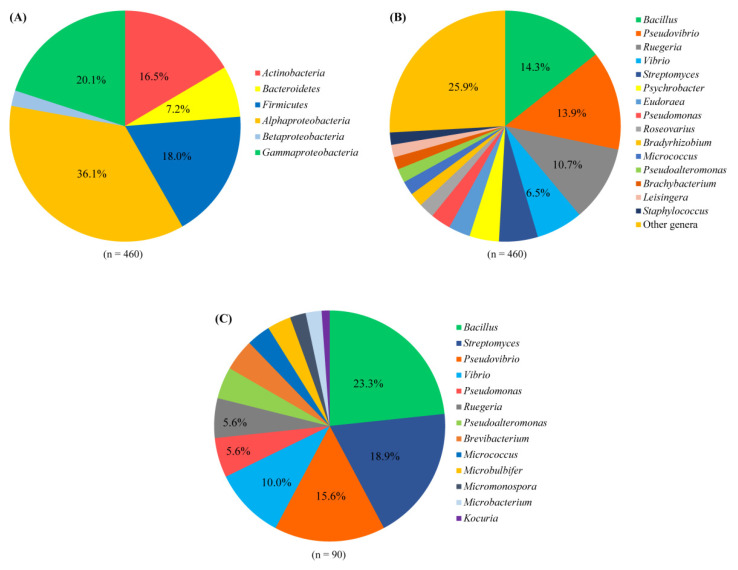
Composition of cultivable bacteria associated with sponges at the phylum level (at the class level for the phylum *Proteobacteria*) (**A**) and the genus level (**B**). Composition of bacteria with antimicrobial activity associated with sponges at the genus level (**C**).

**Figure 2:**
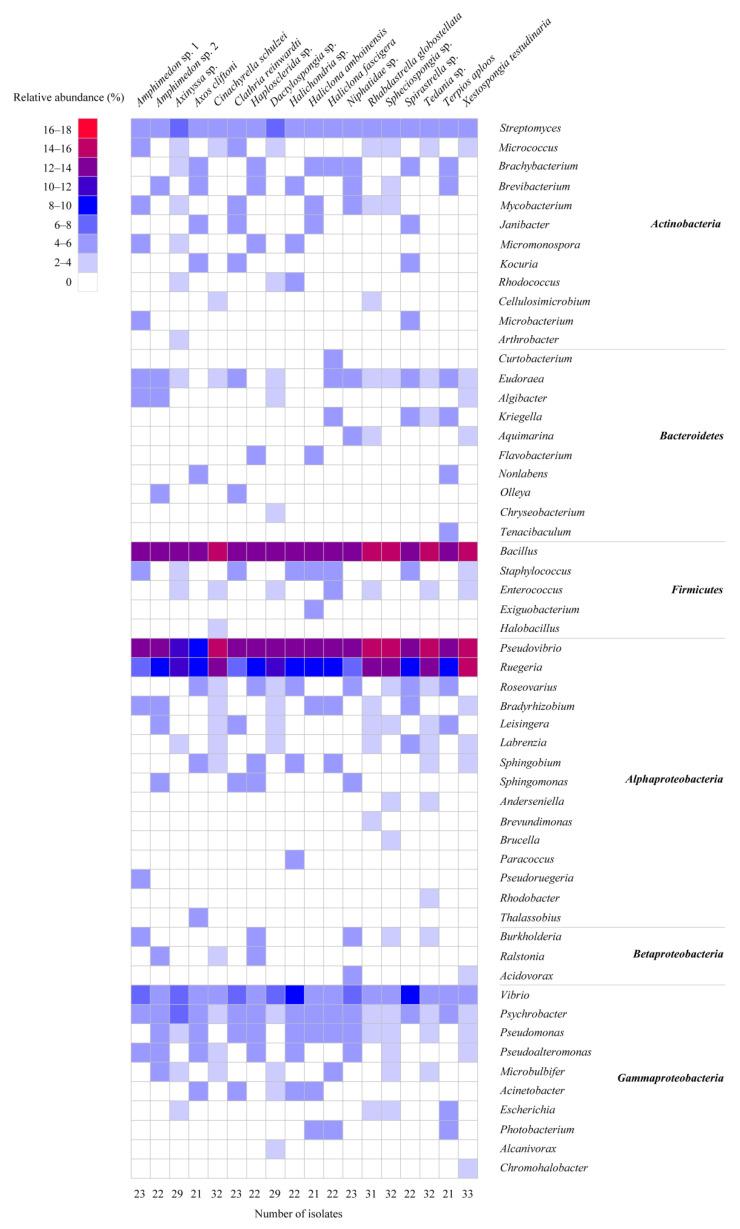
Heatmap of the composition and relative abundance of cultivable bacteria isolated from different sponge species at the genus level.

In total, agar plates with seven different media yielded 473 bacterial colonies from 18 sponge species (Table S1). Of these, 460 bacterial colonies were regrown and identified by their 16S rRNA gene sequences. The isolated strains included four phyla: *Proteobacteria* (*α*-, *γ*- and *β*-*proteobacteria*), *Actinobacteria*, *Firmicutes*, and *Bacteroidetes* (Figure 1A). Among them, the phylum *Proteobacteria* was the most predominant (58.3% of the isolated strains), followed by *Firmicutes* (18.0%) and *Actinobacteria* (16.5%), whereas the phylum *Bacteroidetes* represented 7.2% of the isolated strains. At the genus level, 55 genera were represented, of which the genera *Bacillus*, *Pseudovibrio*, *Ruegeria*, *Vibrio*, and *Streptomyces* were the most predominant, each accounting for more than 5% of all isolated strains (Figure 1B and Figure 2). The number of bacterial genera recovered from sponges ranged from 15 to 21. The composition of cultivable bacteria from different sponges was relatively similar at the genus level (Figure 2).

**Figure 3:**
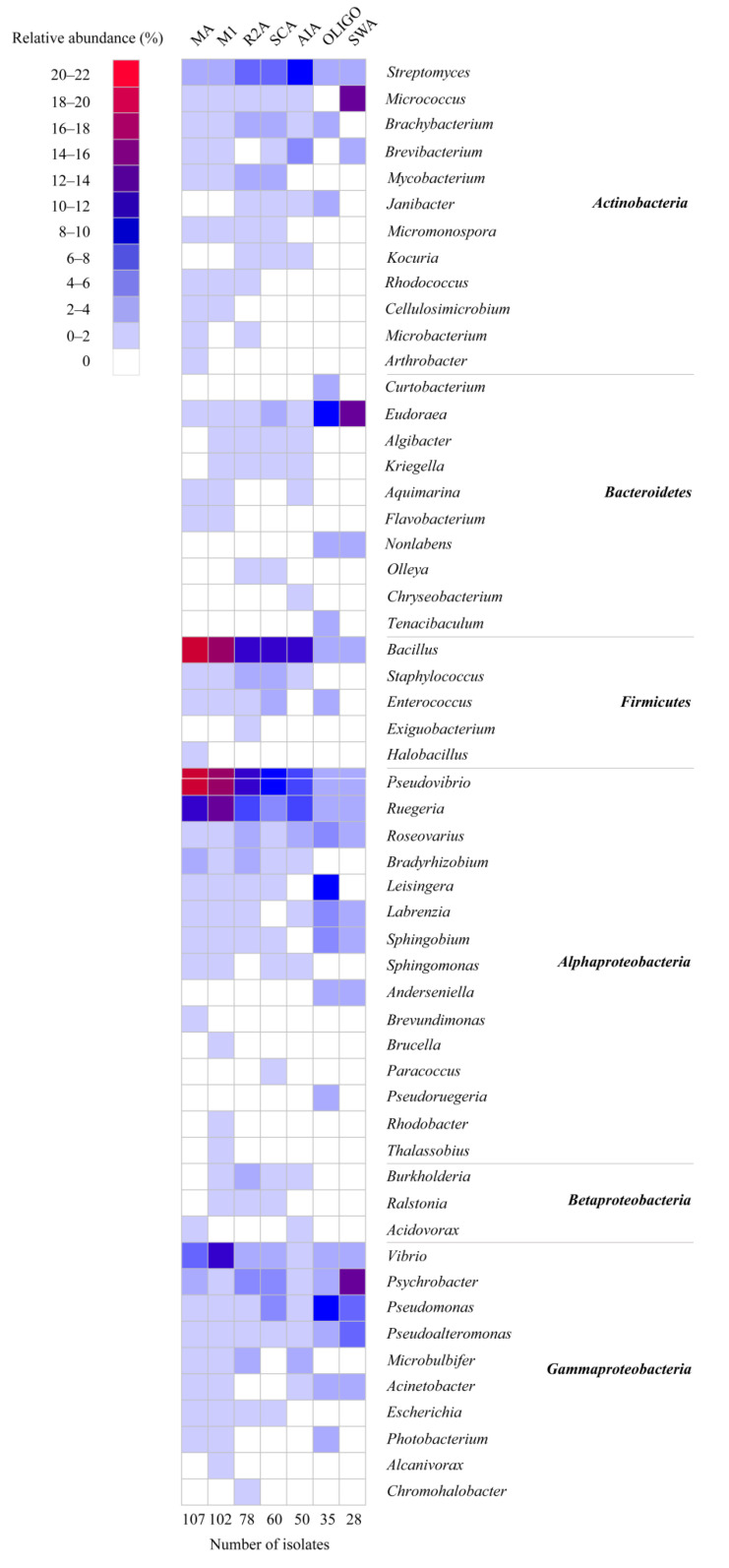
Heat map of the composition and relative abundance of cultivable bacteria isolated from different culture media at the genus level.

The culture media significantly affected the cultivable bacteria (*p*-values of 0.21 (Betadisper) and <0.001 (Adonis)) (Table S2). Although the most abundant genera *Bacillus*, *Pseudovibrio*, *Ruegeria*, *Vibrio*, and *Streptomyces* were isolated on all cultivation media, several genera were only isolated from nutrient-poor media, such as *Arthrobacter*, *Curtobacterium*, *Nonlabens*, *Tenacibaculum*, *Exiguobacterium*, *Anderseniella*, *Pseudoruegeria*, *Thalassobius*, and *Alcanivorax* (Figure 3). None of the isolated strains represented new species as, based on their 16S ribosomal RNA (rRNA) gene sequence, they were all 99–100% similar to known strains (Table S5). The nutrient-rich media resulted in higher numbers of colonies and genera than nutrient-poor media (e.g., OLIGO, SWA) (Table S1). M1 medium yielded the highest number of genera (38), followed by MA medium (35 genera), R2A (33 genera), SCA (30 genera), AIA (27 genera), and OLIGO (23 genera), and the lowest number of genera was observed with SWA, on which 17 genera were recovered (Figure 3). In addition, nutrient-rich media had significantly higher richness, Shannon diversity, and Inverse Simpson diversity, while nutrient-poor media (i.e., OLIGO, SWA) exhibited higher evenness (Tables S3 and S4). The higher alpha diversity indices for rich media may be partially explained by the higher number of colonies obtained from those media (Table S1).

**Figure 4:**
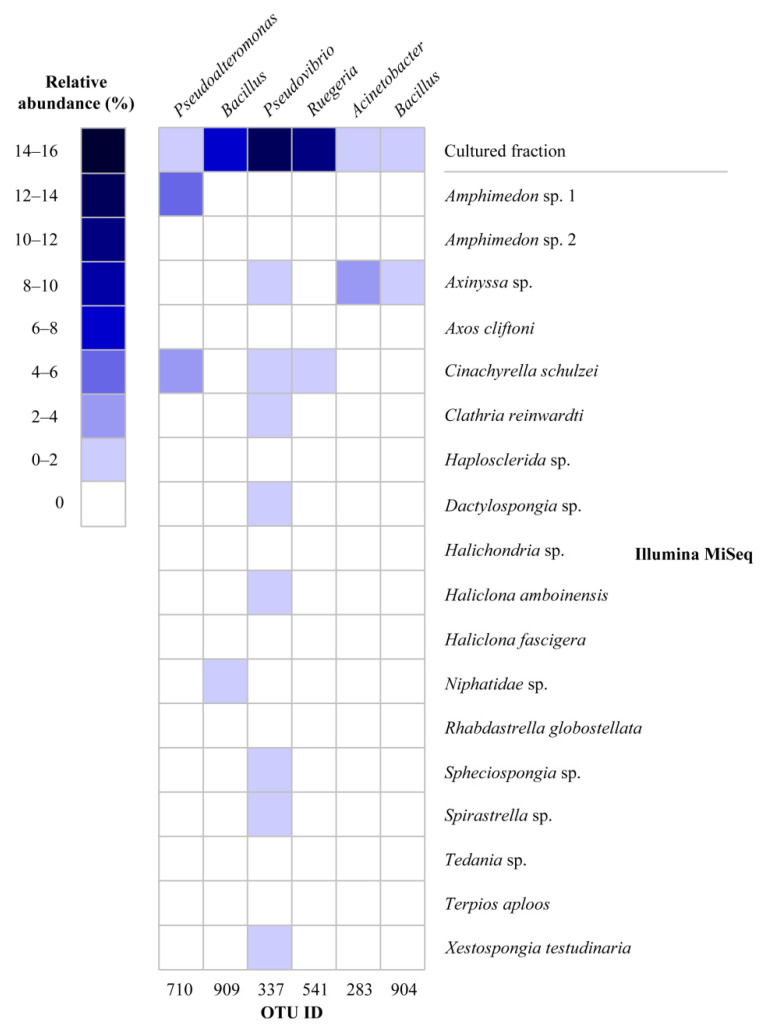
Heat map of relative abundance of shared OTUs recovered by cultivation and cultivation-independent approaches (MiSeq). The relative abundance of OTUs in the cultivable fraction was calculated for total sequences for all sponge species, whereas the relative abundance of OTUs in Illumina MiSeq data was calculated for each sponge species.

The 16S rRNA gene sequences from the isolated strains were compared to sequences that were generated by direct Illumina MiSeq amplicon sequencing analysis of the same sponge samples [28]. Six OTUs out of 921 OTUs detected by MiSeq sequences were also found by cultivation; however, their abundance was distinct for the two methods (Figure 4). For example, OTU909 (*Bacillus*), OTU337 (*Pseudovibrio*), OTU541 (*Ruegeria*), and OTU710 (*Pseudoalteromonas*) were cultivated from all sponge species; however, these OTUs were not detected or detected at very low relative abundance from the same sponge species by MiSeq analysis.

### 2.2. Antimicrobial Activity of Isolated Strains

Cell-free supernatants from cultures of all isolated strains (n = 460) were screened for their antimicrobial activity against seven indicator microorganisms: *Escherichia coli* ATCC 25922, *Pseudomonas aeruginosa* ATCC 25923, *Bacillus subtilis* ATCC 27212, *Staphylococcus aureus* ATCC 12222, *Candida albicans* ATCC 7754, *Salmonella enterica* ATCC 13076, and *Enterococcus faecalis* ATCC 29212. The cell-free culture supernatants of 90 isolated strains (nearly 20%) showed antimicrobial activity against one or more of the tested indicator microorganisms (Table 1). Among them, the cell-free culture supernatants of 57 isolated strains exhibited activity against one indicator strain, while 21 isolated strains exhibited activity against 2 indicator strains, 8 isolated strains exhibited activity against 3 indicator strains, 3 isolated strains exhibited activity against 4 indicator strains, and 1 isolated strain exhibited activity against 5 tested indicator strains. It was observed that the cell-free culture supernatants showed more antimicrobial activity towards Gram-positive bacteria (21 isolated strains against *S. aureus*, 26 isolated strains against *E. faecalis*, and 27 isolated strains against *B. subtilis*) than Gram-negative bacteria (16 isolated strains against *P. aeruginosa*, 17 isolated strains against *S. enterica*, and 20 isolated strains against *E. coli*), whereas the number of isolated strains exhibiting activity against yeast was the lowest (15 isolated strains). The identification of the active isolated strains revealed that they belonged to 13 genera, with *Bacillus*, *Streptomyces*, and *Pseudovibrio* being predominant with 23.3%, 18.9%, and 15.6%, respectively (Figure 1C). 

### 2.3. Isolation and Identification of the Compounds Produced by Bacillus sp. M1_CRV_171

**Figure 5:**
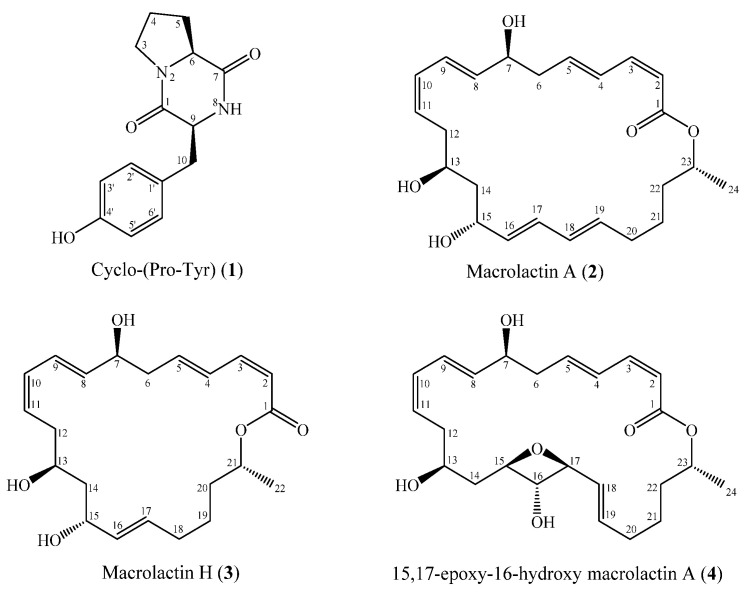
Chemical structures of isolated compounds (**1**–**4**).

Well diffusion assays revealed that *Bacillus* sp. M1_CRV_171 was the only isolated strain to inhibit five reference strains, including Gram-negative bacteria, Gram-positive bacteria, and yeast. Furthermore, this isolated strain was also found in the same sponge by the cultivation-independent method [28], as the 16S rRNA gene sequence of this isolated strain was 100% identical with a bacterial OTU recovered from sponge tissue. Therefore, this isolated strain was subjected to isolation of secondary metabolites. Isolation and purification of ethyl acetate extracts from the culture broth of *Bacillus* sp. M1_CRV_171 led to the isolation of four compounds **1**–**4** (Figure 5). The structures of these compounds were elucidated by the examination of their ESI-MS and NMR (^1^H NMR, ^13^C NMR, HMBC, HSQC) spectra (Figures S1–S20) and comparison with reported data. The compounds were identified as cyclo(L-Pro-L-Tyr) (**1**) [29], macrolactin A (**2**) [30,31], macrolactin H (**3**) [32], and 15,17-epoxy-16-hydroxy macrolactin A (**4**) [33].

### 2.4. Antimicrobial Activity of the Isolated Secondary Metabolites

The four compounds isolated from the strain *Bacillus* sp. M1_CRV_171 were evaluated for their antimicrobial activity against a broad spectrum of microorganisms (Table 2). Compound **1** was inactive against most of the reference strains, except for *S. aureus*, *Vibrio parahaemolyticus*, *Fusarium oxisporum*, and *Ralstonia solani*, with minimum inhibitory concentrations (MICs) > 256 µg/mL. Compounds **2**–**4** exhibited antimicrobial activity against a wide spectrum of reference strains. Compound **2** exhibited antimicrobial activity against *P. aeruginosa*, *S. aureus*, and *Rhodococcus* sp., with MICs of 8, 16, and 32 µg/mL, respectively. Notably, antimicrobial activity against *P. aeruginosa* of compound **2** was comparable to the positive controls tetracycline (MIC > 64 µg/mL), kanamycin (MIC > 256 µg/mL), and ampicillin (MIC > 256 µg/mL). Furthermore, compound **2** exhibited activity against a wide spectrum of tested microorganisms, with MICs ranging from 64 to >256 µg/mL, while it was inactive against *S. enterica*, *E. faecalis*, *Vibrio vulnificus*, and *Vibrio alginolyticus*. Compound **3** showed antimicrobial activity against *E. coli* and *S. aureus*, with MIC values of 16 µg/mL and 32 µg/mL, respectively, which was comparable to the positive controls (ampicillin, kanamycin, and tetracycline), with MIC values ranging from 4 to 16 µg/mL. Compound **3** also exhibited antimicrobial activity against other tested microorganisms, with MICs ranging from 64 to >256 µg/mL. For compound **4**, the strongest activity was found against *E. coli*, with a MIC value of 32 µg/mL. For other tested microorganisms, compound **4** showed antimicrobial activity with MICs ≥ 64 µg/mL.

## 3. Discussion

### 3.1. Cultivable Bacteria from Vietnamese Sponges

It is well known that bacteria associated with marine sponges are phylogenetically diverse and an important source of bioactive compounds, including antimicrobial compounds [22,25,26,27]. The present study aimed to isolate bacteria associated with Vietnamese sponges, which have only been studied very little, and screen for antimicrobial activity of the isolated strains. The 16S rRNA gene sequence analysis showed that cultivable bacteria from the Vietnamese sponges belonged to four phyla, which is much lower compared to the 15 phyla that were detected by direct Illumina MiSeq amplicon sequencing analysis of the same sponge samples [28]. This discrepancy has also been observed in previous investigations of sponge-associated bacteria using both cultivation-dependent and -independent approaches [34,35,36,37,38]. The failure of obtaining the diverse bacteria associated with sponges by cultivation can be partially explained by the failure to mimic the natural growth conditions of sponge-associated bacteria (e.g., host-symbiont interactions, substrates required for growth) in the laboratory. Recent developments in reconstructing composite genomes of currently uncultivable bacteria out of metagenomes have provided clues on growth substrates of some sponge-associated bacteria, e.g., predicted consumption of carnitine, spermidine, and sulfated polysaccharides [39,40], compounds that are hardly ever included in bacterial cultivation media. In addition, extensive cultivation efforts by using alternative techniques (e.g., cultivation media and growth condition diversification, liquid culture, floating filter, diffusion chamber cultivation) have improved the cultivability of sponge-associated bacteria and should be used in more bacterial cultivation experiments with sponges [35,38,41,42].

At the genus level, cultivable bacteria from Vietnamese sponges were predominated by genera such as *Bacillus*, *Pseudovibrio*, and *Ruegeria*, which are commonly isolated from marine sponges [35,36,41,43,44,45,46,47]. A cultivable *Pseudovibrio* sp. has also been found in sponge larvae and may thus represent a bacterium that is vertically transmitted in sponges [45]. Despite their predominance in our cultivation experiment, these genera were not detected, or were detected at very low relative abundance, in the same sponge specimens by direct Illumina MiSeq amplicon sequencing analysis [28]. 

It is possible that these bacteria may be selected for cultivation experiments as they may grow quickly in rich-nutrient media and outcompete other slow-growing species, although they are not abundant in the original inoculum [48].

In addition, several common genera, such as *Pseudovibrio*, *Bacillus*, *Vibrio*, and *Ruegeria* grew well on different cultivation media, suggesting that these genera can adapt well to different cultivation media and conditions. Recent genome studies have reported that genomes of these genera contain the genetic machinery encoding for a versatile metabolism and harbor diverse genomic features linked to symbiosis and lifestyles allowing for host switching [49,50,51,52,53,54,55,56,57,58,59,60,61].

### 3.2. Antimicrobial Activity of Sponge-Associated Bacteria

Marine sponges are sessile organisms, and their defense mechanism against predators (e.g., bacteria, eukaryotic organisms, viruses) is mainly based on the production of a diverse range of secondary metabolite products, allowing efficient chemical protection [62,63,64]. Increasing evidence has shown that many bioactive compounds, including antimicrobial compounds [22,25,26,27], from sponges are produced by their microbial symbionts [23,24,65,66,67,68,69]. The antimicrobial screening assay of cultured bacteria in this study showed that approximately 20% of the isolated strains exhibited antimicrobial activity. It was observed that strains showed more activity against Gram-positive bacteria than Gram-negative bacteria. The difference in membrane structure between Gram-positive and Gram-negative bacteria could partially explain their different sensitivity to antimicrobial agents. The outer membrane of Gram-negative bacteria is covered by a layer of lipopolysaccharide, which protects them from antibiotics attacking the peptidoglycan layer [70]. Among the isolated strains with antimicrobial activity, *Bacillus*, *Streptomyces*, and *Pseudovibrio* were the most frequently obtained. These genera have been reported as major producers of bioactive compounds with antimicrobial activity in sponges [27]. Genome analyses of sponge-derived isolated bacteria from these genera have reported that they contain a large number of secondary metabolite biosynthesis gene clusters [50,53,54,55,71,72,73]. One *Bacillus* isolated strain (M1_CRV_171) inhibited the growth of Gram-positive and Gram-negative bacteria and fungi. The genus *Bacillus* produces versatile antimicrobials with diverse structures (e.g., polyketides, ribosomal peptides, non-ribosomal peptides, lipopeptides, macrolactones, polypeptides, isocoumarins) [74,75,76]. A large-scale analysis of 1566 genomes of *Bacillus* strains resulted in identifying nearly 20,000 biosynthetic gene clusters (BGCs) [77]. Of these, a large number of BGCs encode the enzymes required for the production of unknown compounds, indicating *Bacillus* as a prolific source of secondary metabolites. It is estimated that at least 4–8% of the genome of *Bacillus* is devoted to synthesizing antimicrobial compounds [78,79,80].

Among the antimicrobial isolated strains, the strain *Bacillus* sp. M1_CRV_171 is one of the cultured strains that was found in the cultivation-independent bacterial community analysis (OTU909). Therefore, this isolated strain was subjected to investigation of its secondary metabolites. From culture broth of *Bacillus* sp. M1_CRV_171, one cyclic dipeptide, cyclo(L-pro-L-tyr) (**1**), was isolated and purified. This cyclic dipeptide has been reported to be produced by different bacteria (i.e., *Bacillus*, *Pseudomonas*, *Ruegeria*, *Psychrobacter*) associated with different sponges [81,82,83,84,85,86], indicating that it may play a role in the sponge holobiont. The antimicrobial assays in our study showed that cyclo(L-pro-L-tyr) exhibited antimicrobial activity against *S. aureus*, *V. parahaemolyticus*, *F. oxisporum*, and *R. solani* and showed no activity against the other tested microorganisms. This confirms previous bioactivity investigations of cyclo(L-pro-L-tyr), which reported that this cyclic dipeptide exhibits antimicrobial activity against several bacteria and plant pathogenic fungi, such as *S. epidermis*, *Klebsiella pneumoniae*, *Proteus mirabilis*, and *V. cholerae* [87], *Micrococcus luteus*, *S. aureus*, *S. enterica*, *E. coli*, and *Fusarium* sp. [88], *Xanthomonas axonopodis pv. citri*, and *Ralstonia solanacearum* [29], *F. oxysporum* and *Penicillium* sp. [89], *A. niger* [90], *Phytophthora infestans*, and *Plasmopara viticola* [91,92]. However, cyclic dipeptides, including cyclo(L-pro-L-tyr), have gained more attention because of their role as quorum sensing (QS) signal molecules [93,94,95,96,97,98,99,100]. It has been reported that sponge-associated bacteria, as well as cyclic dipeptides isolated from sponge-associated bacteria, are involved in QS by activating acyl homoserine lactones (AHLs) bioreporters [85]. AHLs are QS signal molecules mainly produced by Gram-negative bacteria and may directly bind to transcription factors (e.g., LuxR) to regulate gene expression [101,102], indicating that these bacteria and cyclic dipeptides may interact with AHL producers and play a certain role in QS. Interestingly, previous studies have demonstrated the production of AHLs by both sponges and their associated bacteria [103,104,105,106], and these compounds may thus be involved in communication in the sponge holobiont.

Furthermore, three macrolides **2**–**4** were isolated from culture broth of *Bacillus* sp. M1_CRV_171. Macrolide compounds are widely used as antibiotics in clinics (e.g., erythromycin) and display different biological activities, including modulating inflammation [107]. Macrolactins are a large group of macrolide antibiotics with a 22- to 24-member lactone ring mainly discovered in marine microorganisms, especially in the genus *Bacillus* [74,108]. Although numerous macrolide compounds have been isolated and identified from sponges and their symbionts [108], this is the first study to report the isolation of macrolactins from sponge-associated bacteria. Macrolactin A (**2**) has been frequently isolated from different bacteria [30,31,32,109,110,111,112], whereas macrolactin H (**3**) and 15, 17-epoxy-16-hydroxy macrolactin A (**4**) have been rarely isolated [32,33]. Previous bioassays revealed that macrolactin A exhibited antimicrobial activity against Gram-negative bacteria (i.e., *E. coli*), Gram-positive bacteria (i.e., *S. aureus*, methicillin-sensitive *S. aureus* (MSSA), methicillin-resistant *S. aureus* (MRSA), *B. subtilis*, *E. faecalis,* and vancomycin-resistant enterococci), as well as fungi (i.e., *Botrytis cinerea,* *A. niger*, *B. cinerea*, *Colletotrichum acutatum*, *R. solani*, *S. cerevisiae*, *C. albicans, Pestalotiopsis theae*, and *C. gloeosporioides*) [30,31,32,109,110,111,112]. The antimicrobial assays in our studies showed similar results, in which macrolactin A exhibited antimicrobial activity against a broad spectrum of reference microorganisms. Furthermore, antibacterial activity of macrolactin A against *Vibrio* spp. was reported for the first time in this study. The biological properties of macrolactin H (**3**) and 15, 17-Epoxy-16-hydroxy macrolactin A (**4**) are less well known. The isolation and antimicrobial activity of these two compounds have been reported once by [33] and [32], respectively. Macrolactin H was reported to exhibit antimicrobial activity against *S. aureus* (MIC = 10 µg/mL) and *B. subtilis* (MIC = 60 µg/mL) [32], whereas 15, 17-epoxy-16-hydroxy macrolactin A was reported to exhibit antimicrobial activity against *B. subtilis*, *E. coli*, and *S. cerevisiae* with the same MICs of 0.16 µM [33]. Although macrolactins show antimicrobial activity against a broad spectrum of microorganisms, including Gram-negative and Gram-positive bacteria and fungi, the mode of action of macrolactins is not understood. However, several recent studies indicate that some macrolactin compounds, such as 7-O-Malonyl Macrolactin A, induce disruption of cell division [31] and macrolactin N inhibits bacterial peptide deformylase [113], whereas bamemacrolactin C affects fungal mycelial morphology, the cell wall, and protein expression by interrupting the oxidative phosphorylation [114]. In addition, Zotchev et al. [115] speculate that the antibacterial activity of macrolactins could be due to the inhibition of the H^+^-transporting two-sector ATPase, which is essential for viability of bacterial cells.

## 4. Materials and Methods 

### 4.1. Collection and Identification of Sponges

Sponge specimens were collected by Scuba diving from May to September 2015 from the central coastal region of Vietnam at 5–10 m depth and identified using molecular markers (18S rRNA and cytochrome oxidase I (COI) genes) in a previous study: *Axinyssa* sp., *Xestospongia testudinaria*, *Clathria reinwardti*, *Spirastrella* sp., *Dactylospongia* sp., *Haliclona amboinensis*, *Cinachyrella schulzei*, *Niphatidae* sp., *Haliclona fascigera*, *Amphimedon* sp. (1), *Amphimedon* sp. (2), *Haplosclerida* sp., *Rhabdastrella globostellata*, *Spheciospongia* sp., *Halichondria* sp., *Tedania* sp., *Terpios aploos*, and *Axos cliftoni* [28].

### 4.2. Isolation of Bacteria from Sponges

The sponge specimens were rinsed three times with sterile seawater to remove bacteria attached to the surface of sponges. The specimens were then further cleaned with a sterile scalpel in order to remove sediment and other organisms attached to the sponge. A piece of sponge specimen (~1 cm^3^) was crushed with a sterile mortar and pestle and then homogenized in 10 volumes of sterile seawater. The cell suspension was serially diluted till 10^−6^ and subsequently plated onto seven different media: MA (0.5% peptone, 0.1% yeast extract, 1.5% agar) (modified from [116]); M1 (1.0% starch, 0.4% yeast extract, 0.2% peptone, 1.5% agar) (modified from [117]); R2A (0.05% yeast extract, 0.05% glucose, 0.05% peptone, 0.05% casein hydrolysate, 0.05% starch, 0.03% sodium pyruvate, 0.03% K_2_HPO_4_, 0.005% MgSO_4_, 1.5% agar) (modified from [118]); SCA (0.5% starch, 0.002% casein, 0.1% KNO_3_, 0.1% NaCl, 0.1% K_2_HPO_4_, 0.5 mL/L MgSO_4_ 100 mM; 0.5 mL/L FeSO_4_ 100 mM, 0.5 mL/L CaCO_3_ 100 mM, 1.5% agar) (modified from [119]); AIA (0.01% peptone, 0.001% L-asparagine, 0.4% sodium propionate, 0.005% K_2_HPO_4_, 0.001% MgSO_4_, 0.001 g/L FeSO_4_, 1 mL/L glycerol, 1.5% agar) [35]; OLIGO (0.05% yeast extract, 0.05% tryptone, 0.01% sodium glycerolphosphate, 1.5% agar) [120]; SWA (1.5% agar). Nalidixic acid and cycloheximide were supplemented into culture media at concentrations of 100 µg/L and 25 µg/L, respectively, to reduce growth of fast-growing Gram-negative bacteria and fungi. All culture media were prepared with sterile natural seawater at pH 7.8 and all plates were produced in triplicate and incubated for 3–5 days at 30 °C. The pure cultures were obtained by streaking on agar plates and were stored with 20% glycerol (*v*/*v*) at −80 °C at the Mientrung Institute for Scientific Research, Hue city, Thua Thien Hue province, Vietnam.

### 4.3. Screening for Antimicrobial Activity of the Isolates 

The isolates (n = 460) were incubated in M1 broth under aerobic conditions on a rotary shaker (150 rpm) at 30 °C until they reached the stationary phase, then the cultures were centrifuged at 14,000× *g* for 10 min. The cell-free supernatants were used for screening their antimicrobial activity.

Antimicrobial activity of the isolates was tested against seven indicator strains, including Gram positive bacteria: *Bacillus subtilis* ATCC 6633, *Staphylococcus aureus* ATCC 25923, *Enterococcus faecalis* ATCC 29212, Gram negative bacteria: *Escherichia coli* ATCC 25922, *Pseudomonas aeruginosa* ATCC 27853, *Salmonella enterica* ATCC 13076, and the yeast *Candida albicans* ATCC 10231 using the agar well diffusion method. The indicator bacteria were grown in nutrient broth (NB), whereas the yeast was grown in potato dextrose agar (PDA). All indicator strains were grown aerobically on a rotary shaker (150 rpm) overnight and then the density of the strains in cultures was adjusted to an OD_600_ of 0.5. Subsequently, 100 µL of these growing cultures was spread on Mueller-Hinton (MH) agar (for bacteria) and RMPI agar supplemented with 2% glucose (for yeast). The agar wells were prepared by using a sterilized cork borer (6 mm in diameter). Antimicrobial activity of the isolates was examined by adding 100 µL of the cell-free supernatant of each isolate to the wells. For the negative controls, 100 µL of the respective sterile, uninoculated liquid media was added to the 6 mm wells. For positive controls, Ampicillin (10 µg), Kanamycin (30 µg), and Tetracycline (30 µg) were added to the 6 mm wells. The assay was conducted in triplicate for each of the indicator strains.

The agar plates were incubated at 35 °C for 24 h (for the indicator bacteria) and 28 °C for 48 h (for the yeast). After incubation, antimicrobial activity of isolates was determined based on the formation of inhibition zones around wells.

### 4.4. Identification of the Isolates by 16S rRNA Gene Analysis

The glycerol stocks of picked isolates were regrown in the liquid media that were used for their initial isolation. The regrown isolates were identified by colony PCR. For cell lysis, the liquid cultures (2 mL) were centrifuged at 14,000× *g* for 10 min and the obtained pellets were suspended in 50 µL nuclease-free water. Subsequently, the cell suspension was stored at −20 °C for 2 h, followed by incubation at 98 °C for 10 min. The 16S rRNA gene of isolates was directly amplified with universal primers: 27f and 1492r [121] through the following PCR program: an initial denaturation at 94 °C for 5 min, followed by 30 cycles of denaturation at 94 °C for 1 min, annealing at 56 °C for 50 s, amplification at 72 °C for 1.5 min, and a final extension at 72 °C for 7 min. The 16S rRNA gene sequencing was carried with a ABI PRISM 3100^®^ Genetic Analyzer (Applied Bioscience and Hitachi, Foster city, CA, USA) with primers 27f and 907r/1492r [122] to facilitate alignment with Illumina MiSeq sequencing reads that were used to characterize the prokaryotic communities of the sponges previously [28]. The sequences were quality checked, and low-quality regions were removed from the sequence ends using BioEdit software v.7.2.6.1. The quality-checked sequences of the isolates were compared to available sequences in the NCBI GenBank using BLAST v.2.7.1+ [123], with the algorithm megablast and the database nt (5 September 2018).

Alpha diversity indices (i.e., observed richness *S*, Shannon diversity index *H*, inverse Simpson *In*, Pielou’s evenness *J*) were calculated in R v.3.0.3 with the vegan package [124,125]. The relative abundance of the cultivable bacteria at the genus level from different sponge species and media was visualized with JColorGrid [126].

To compare the sequences of the isolates to the cultivation-independent fraction, 16S rRNA gene sequences of the isolates in this study were blasted against Illumina MiSeq sequences of prokaryotic OTUs from the same sponge specimens described in the previous study [28] using a BLAST search [123]. A match was defined as sequences shared between the cultivation-dependent fraction and cultivation-independent fraction if their identity was 100%. The relative abundance of the shared OTUs from the cultivation fraction and cultivation-independent fraction was visualized with JColorGrid [126].

### 4.5. Cultivation, Extraction, and Isolation of Secondary Metabolites

The strain *Bacillus* sp. M1_CRV_171 was cultured in a 250 mL flask containing 125 mL M1 broth at 30 °C for 48 h in a shaking incubator. The culture was transferred to a 5 L bioreactor (BioFlo 120, Eppendorf, Hamburg, Germany) containing 2.5 L M1 broth with an inoculum of 5% (*v*/*v*). The reactor was operated at 30 °C with agitation of 150 rpm for 48 h. Subsequently, the culture was used to inoculate a 100 L bioreactor (BioFlo 610, Eppendorf, Hamburg, Germany) containing 50 L M1 medium with an inoculum 5% (*v*/*v*). The reactor was operated at 30 °C with agitation of 150 rpm for 7 days. The culture broth (50 L) was extracted with ethyl acetate (25 L × 3 times) at room temperature. The ethyl acetate extract was concentrated under a reduced pressure to yield 11.0 g of crude residue. The residue was subjected to a silica gel chromatography column (CC) (Kiesel gel 60, 70–230 mesh, and 230–400 mesh, Merck, Germany) and eluted with a dichloromethane/methanol gradient (100/1 to 1/100, *v*/*v*) to give seven fractions VKB1-VKB7. The fraction VKB2 was chromatographed on an RP-18 column (30–50 μm, Fuji Silysia Chemical Ltd., Kasugai Aichi, Japan) and eluted with methanol/water (1/1, *v*/*v*) to obtain compound **2** (17.8 mg) and a subfraction VKB2.1. Compound **1** (6.3 mg) was obtained from fraction VKB2.1 by silica gel CC, eluted with dichloromethane/methanol (30/1, *v*/*v*). The fraction VKB3 was subjected to a silica gel CC and eluted with dichloromethane/methanol (30/1, *v*/*v*) to give two smaller fractions VKB3.1-VKB3.2. The fraction VKB3.1 was further separated on an RP-18 column and eluted with methanol/water (1.5/1, *v*/*v*) to give compound **3** (3.7 mg). Compound **4** (3.4 mg) was purified from the fraction VKB3.2 on an RP-18 column eluted with methanol/water (1.5/1, *v*/*v*).

The electrospray ionization mass spectra (ESI-MS) of the compounds were recorded on a MicroQ-TOF III mass spectrometer (Bruker Daltonics, Bremen, Germany), and the NMR spectra (^1^H NMR, ^13^C NMR, HMBC, HSQC) of the compounds were recorded on a Bruker Avance III HD 500 FT NMR spectrometer (Bruker BioSpin, Rheinstetten, Germany) with TMS as an internal standard. The optical rotations were recorded on a JASCO P-2000 digital polarimeter (JASCO, Tokyo, Japan). The structures of the isolated compounds were elucidated by the examination of their ESI-MS, NMR spectra and compared with reported data.

Compound **1** (Cyclo(L-Pro-L-Tyr)): white solid; ESI-MS *m/z* 261.1 [M+H]^+^; [α]_D_^24^ = -17.5 (c 0.05, MeOH); ^1^H NMR (500 MHz, CD_3_OD) *δ*_H_ (ppm): *δ*_H_ 1.82 (2H, m, H-4), 2.11 (2H, m, H-5), 3.07 (2H, m, H-10), 3.37 (1H, m, H_a_-3), 3.57 (1H, m, H_b_-3), 4.07 (1H, m, H-6), 4.37 (1H, m, H-9), 6.72 (2H, d, *J* = 8.5 Hz, H-3’, 5’), 7.05 (2H, d, *J* = 8.5 Hz, H-2’, 6’); ^13^C NMR (125 MHz, CD_3_OD) *δ*_C_ (ppm): *δ*_C_ 22.7 (C-4), 29.3 (C-5), 37.6 (C-10), 45.9 (C-3), 57.9 (C-9), 60.0 (C-6), 116.2 (C-3’, 5’), 127.6 (C-1’), 132.1 (C-2’, 6’), 157.6 (C-4’), 166.9 (C-1), 170.8 (C-7).

Compound **2** (Macrolactin A): amorphous solid; ESI-MS *m/z* 403.2 [M+H]^+^; [α]_D_^24^ = -8.3 (c 0.10, MeOH); ^1^H NMR (500 MHz, CD_3_OD) *δ*_H_ (ppm): *δ*_H_ 1.27 (3H, d, *J* = 6.5 Hz, H-24), 1.52 (2H, m, H-21), 1.59 (1H, m, H_a_-22), 1.63 (2H, m, H-14), 1.65 (1H, m, H_b_-22), 2.12 (1H, m, H_a_-20), 2.20 (1H, m, H_b_-20), 2.34 (1H, m, H_a_-12), 2.44 (2H, m, H-6), 2.50 (1H, m, H_b_-12), 3.87 (1H, m, H-13), 4.28 (1H, m, H-7), 4.32 (1H, m, H-15), 5.02 (1H, m, H-23), 5.55 (1H, m, H-11), 5.56 (1H, m, H-2), 5.57 (1H, m, H-16), 5.66 (1H, m, H-19), 5.77 (1H, dd, *J* = 6.0, 15.0 Hz, H-8), 6.05 (1H, m, H-18), 6.10 (1H, m, H-17), 6.13 (1H, m, H-10), 6.19 (2H, m, H-5), 6.58 (1H, dd, *J* = 11.0, 15.0 Hz, H-9), 6.65 (1H, t, *J* = 11.5 Hz, H-3), 7.23 (1H, m, H-4); ^13^C NMR (125 MHz, CD_3_OD) *δ*_C_ (ppm): *δ*_C_ 20.1 (C-24), 25.6 (C-21), 32.9 (C-20), 36.1 (C-22), 36.4 (C-12), 42.8 (C-6), 43.9 (C-14), 69.2 (C-13), 69.8 (C-15), 72.2 (C-23), 72.3 (C-7), 117.9 (C-2), 125.9 (C-9), 128.4 (C-11), 130.2 (C-4), 131.2 (C-17), 131.3 (C-10), 131.7 (C-18), 135.1 (C-19), 135.2 (C-16), 137.5 (C-8), 142.1 (C-5), 144.9 (C-3), 168.0 (C-1).

Compound **3** (Macrolactin H): amorphous solid; ESI-MS *m/z* 377.3 [M+H]^+^; [α]_D_^24^ = -24.6 (c 0.05, MeOH); ^1^H NMR (500 MHz, CD_3_OD) *δ*_H_ (ppm): *δ*_H_ 1.27 (3H, d, *J* = 6.5 Hz, H-22), 1.40 (2H, m, H-19), 1.58 (1H, m, H_a_-12), 1.59 (2H, m, H-14), 1.65 (1H, m, H_a_-20), 2.08 (2H, m, H-18), 2.33 (1H, m, H_b_-12), 2.45 (2H, m, H-6), 4.26 (1H, m, H-15), 4.28 (1H, m, H-21), 4.32 (1H, m, H-13), 4.98 (1H, m, H-7), 5.46 (1H, m, H-11), 5.55 (2H, m, H-2, 16), 5.59 (1H, m, H-4), 5.63 (1H, m, H-10), 5.65 (2H, m, H-3, 17), 5.76 (1H, m, H-8), 6.17 (1H, m, H-5), 6.75 (1H, m, H-9); ^13^C NMR (125 MHz, CD_3_OD) *δ*_C_ (ppm): *δ*_C_ 166.3 (C-1), 118.0 (C-2), 144.8 (C-3), 130.4 (C-4), 141.7 (C-5), 42.5 (C-6), 72.0 (C-7), 137.2 (C-8), 126.1 (C-9), 131.0 (C-10), 134.7 (C-11), 36.5 (C-12), 69.7 (C-13), 44.6 (C-14), 70.2 (C-15), 128.3 (C-16), 131.7 (C-17), 32.9 (C-18), 25.9 (C-19), 36.6 (C-20), 70.8 (C-21), 20.1 (C-22).

Compound **4** (15,17-epoxy-16-hydroxy macrolactin A): amorphous powder; ESI-MS *m/z* 419.3 [M+H]^+^; [α]_D_^24^ = -37.2 (c 0.01, MeOH); ^1^H NMR (500 MHz, CD_3_OD) *δ*_H_ (ppm): *δ*_H_ 1.28 (3H, d, *J* = 6.0 Hz, H-24), 1.40 (1H, m, H_a_-14), 1.52 (2H, m, H-21), 1.67 (2H, m, H-22), 1.99 (1H, m, H_b_-14), 2.05 (1H, m, H_a_-20), 2.11 (1H, m, H_b_-20), 2.13 (1H, m, H_a_-12), 2.42 (1H, m, H_a_-6), 2.51 (1H, m, H_b_-6), 2.57 (1H, m, H_b_-12), 2.94 (1H, t, *J* = 9.0 Hz, H-16), 3.46 (2H, m, H-13, 17), 3.54 (1H, m, H-15), 4.27 (1H, m, H-7), 4.98 (1H, m, H-23), 5.46 (1H, m, H-11), 5.54 (1H, d, *J* = 11.5 Hz, H-2), 5.64 (1H, m, H-8), 5.67 (1H, m, H-18), 5.70 (1H, m, H-19), 6.07 (1H, t, *J* = 11.0 Hz, H-10), 6.27 (1H, m, H-5), 6.56 (1H, dd, *J* = 11.0, 15.0 Hz, H-9), 6.67 (1H, t, *J* = 11.5 Hz, H-3), 7.25 (1H, m, H-4); ^13^C NMR (125 MHz, CD_3_OD) *δ*_C_ (ppm): *δ*_C_ 19.8 (C-24), 26.6 (C-21), 34.4 (C-20), 35.1 (C-12), 36.8 (C-22), 40.8 (C-14), 42.7 (C-6), 72.9 (C-23), 73.1 (C-7), 73.9 (C-15), 76.0 (C-13), 77.7 (C-16), 80.2 (C-17), 118.2 (C-2), 127.7 (C-9), 129.1 (C-18), 129.5 (C-11), 130.6 (C-4), 131.4 (C-10), 132.2 (C-19), 136.2 (C-8), 141.4 (C-5), 144.6 (C-3), 168.2 (C-1).

### 4.6. Antimicrobial Activity of the Secondary Metabolites

Antimicrobial activities of the isolated secondary metabolites **1**–**4** were tested against a broad spectrum of reference microorganisms. Besides the seven reference microorganisms used for antimicrobial screening assays of isolated strains (see above), antimicrobial activity was evaluated against several microbial plant and aquaculture pathogens isolated in Vietnam: *P. putida* MISR 71218, *Rhodococcus* sp. MISR 16518, *B. cereus* MISR 12818, *Vibrio parahaemolyticus* MISR 21116, *V. vulnificus* MISR 20716, *V. alginolyticus* MISR 30816, *Aspergillus niger* MISR-11215, *Fusarium oxisporum* MISR 20415, and *Rhizoctonia solani* MISR 11115.

MICs of the secondary metabolites against reference microorganisms were determined using the broth microdilution method. Determination of MICs of the isolated compounds against the indicator bacteria was performed according to recommendations of the European Committee on Antimicrobial Susceptibility Testing (EUCAST) discussion document E.Dis 5.1 [127]. Briefly, the isolated compounds were dissolved in dimethyl sulfoxide (DMSO) and serially two-fold diluted in MH broth to a concentration range that was twice the desired final concentration (0.5–512 µg/mL) and obtained by adding an equal volume of indicator bacteria cell suspension (see below). 100 µL of the compound solutions were added to wells of 96-well plates. The indicator Bacteria were then incubated overnight in NB on a rotary shaker (150 rpm) at 37 °C, then the density of strains was adjusted to a McFarland standard of 0.5, which contains approximately 1.5 × 10^8^ CFU/mL (range 1–2 × 10^8^ CFU/mL). The bacterial solution was subsequently diluted 150-fold in MH broth to reach a starting inoculum of 1 × 10^6^ CFU/mL. Finally, 100 µL of this inoculum was added to wells containing 100 µL of the compound solution (see above) to obtain a final inoculum of 5 × 10^5^ CFU/mL. The plates included growth control wells (inoculated in compound-free medium) and sterile (uninoculated) wells. The plate was incubated at 37 °C for 24 h, then the absorbance at 630 nm was measured using a microplate reader. The MIC was defined as the lowest concentration of a compound, at which there was no visible growth of indicator bacteria.

Determination of MICs of the isolated compounds against the yeast (*C. albicans*) was performed as recommended in the EUCAST definitive document E.DEF 7.3 [128]. The isolated compound solutions were diluted to the same concentrations (0.5–512 µg/mL) as the antibacterial assay in double strength RPMI 1640 broth (with L-glutamine and a pH indicator but without bicarbonate), supplemented with glucose to a final concentration of 2% (RPMI 2% G), similar to the above-described concentrations. The yeast strain was grown on PDA at 35 °C for 48 h, then colonies were suspended in 10 mL distilled water. The suspension was homogenized by vortexing for 15 s. Subsequently, the cell density was adjusted to the 0.5 McFarland standard, which contains approximately 1–5 × 10^6^ CFU/mL by measuring the absorbance at a wavelength of 530 nm. The yeast solution was diluted 10-fold in sterile distilled water to obtain a starting inoculum of 1–5 × 10^5^ CFU/mL, and 100 µL of this inoculum was then added to wells containing 100 µL of the compound solution (see above) to obtain a final inoculum of 0.5–2.5 × 10^5^ CFU/mL. The plates included growth control wells (inoculated in compound-free medium) and sterile (uninoculated) wells. The plate was incubated at 35 °C for 24 h, then the absorbance at 530 nm was measured using a microplate reader. The MICs were defined as the lowest concentration of antifungal compounds, at which there was no visible growth of the indicator yeast.

Determination of MICs of the isolated compounds against the filamentous fungi was performed as recommended in the EUCAST definitive document E.DEF 9.3 [129]. For the filamentous fungi, the microdilution was performed as described for the yeast, with an exception for the preparation of the inoculum. Fungi were grown on PDA at 28 °C for 5 days, and the fungal colonies were covered with 10 mL distilled water supplemented with 0.1% Tween 20, and suspensions were made by scraping the surface with a sterile loop. Heavy particles in the suspension were allowed to settle for 15 to 20 min at room temperature. The suspension in the upper clear phase was transferred to sterile tubes and homogenized by vortexing for 15 s. The suspension was filtered using Whatman filter paper no. 1 with a pore size of 11 µm to remove large hyphal fragments. The optical density of the suspensions was recorded at 530 nm and then adjusted to transmittance of 80 to 85%. The number of colony-forming units was quantified by plating 100 µL of suspension on PDA and counting the colonies after incubating at 28 °C for 5 days. The inoculum concentrations used ranged from 2 to 5 × 10^6^ CFU/mL. Each suspension was diluted (1:10) with sterile distilled water to obtain the working inoculum concentration of 2 to 5 × 10^5^ CFU/mL. An aliquot of 100 µL of this inoculum was then added to wells containing 100 µL of the compound solution (see above) to obtain a final inoculum of 1–2.5 × 10^5^ CFU/mL. The plates included growth control wells (inoculated in compound-free medium) and sterile (uninoculated) wells. The plate was incubated at 28 °C for 48 h, and MICs of the antifungal compounds were determined visually as the lowest concentration, at which there was no visible growth of the fungi.

The antibiotics ampicillin, kanamycin, tetracycline, and miconazole were used as positive controls.

## 5. Conclusions

This study yielded cultivable bacteria from 18 Vietnamese sponge species and represented four bacterial phyla and 55 genera. The cultivable bacteria were predominantly members of the genera *Bacillus*, *Pseudovibrio*, *Ruegeria*, *Vibrio*, and *Streptomyces*. A big gap was observed between bacterial communities detected by cultivation-dependent and cultivation-independent approaches. Nevertheless, antimicrobial assays showed that 90 cultivable bacteria in this study exhibited antimicrobial activity against a large number of indicator microorganisms. From *Bacillus* sp. M1_CRV_171, the isolated strain with the most versatile antimicrobial activity, four known compounds with antimicrobial activity against a wide spectrum of indicator microorganisms were isolated: the cyclic dipeptide, cyclo(L-pro-L-tyr) and three macrolactins. Although these results confirm that many sponge-associated bacterial isolates have antimicrobial activity, they also stress the risks of rediscovery of compounds based on isolates obtained through traditional techniques. Therefore, the uncultivated majority of the bacteria should be targeted to get access to the full arsenal of bioactive molecules from sponges.

## Figures and Tables

**Table 1:** Antimicrobial activity of isolated bacterial strains.

Isolated Strains	Genus	Inhibition Zone Diameter (mm)						
		Gram-Negative			Gram-Positive			Yeast
		SE	EC	PA	SA	EF	BS	CA
MA_AMC_32	*Bacillus*	-	-	-	10 ± 2	-	-	-
MA_AMQ_66	*Bacillus*	-	7 ± 2	-	-	-	8 ± 3	-
MA_AXT_69	*Bacillus*	7 ± 2	-	-	-	-	-	-
MA_AXT_70	*Bacillus*	5 ± 2	-	8 ± 3	6 ± 3	-	8 ± 3	-
MA_AXC_75	*Bacillus*	-	7 ± 2	-	-	-	-	-
MA_CIS_78	*Bacillus*	-	-	-	-	-	-	6 ± 2
M1_CRV_171	*Bacillus*	-	5 ± 2	8 ± 2	7 ± 3	10 ± 3	-	6 ± 2
M1_DAS_199	*Bacillus*	-	-	12 ± 3	-	-	-	-
M1_HAA_234	*Bacillus*	-	-	-	-	5 ± 1	11 ± 3	7 ± 2
M1_HAA_246	*Bacillus*	-	-	-	6 ± 2	-	-	-
M1_NIS_274	*Bacillus*	5 ± 2	-	-	-	-	-	-
R2A_NIS_276	*Bacillus*	-	-	-	-	-	12 ± 3	-
R2A_RHG_312	*Bacillus*	-	-	-	5 ± 2	-	-	-
R2A_SPV_326	*Bacillus*	-	-	7 ± 2	-	5 ± 2	7 ± 2	-
R2A_SPV_338	*Bacillus*	-	4 ± 1	-	-	3 ± 1	-	6 ± 2
SCA_SPS_344	*Bacillus*	-	-	-	-	-	4 ± 1	-
SCA_TES_347	*Bacillus*	-	-	-	-	-	-	4 ± 1
AIA_TEA_438	*Bacillus*	-	-	-	-	4 ± 1	-	-
AIA_XES_454	*Bacillus*	-	-	15± 3	-	-	-	-
AIA_XES_458	*Bacillus*	12 ± 3	-	-	-	-	-	10 ± 3
M1_HAF_272	*Bacillus*	-	10 ± 3	-	-	-	8 ± 3	-
AIA_SPV_375	*Brevibacterium*	-	-	-	-	12 ± 3	-	-
AIA_HAS_264	*Brevibacterium*	-	-	-	-	-	4 ± 1	-
M1_AXC_175	*Brevibacterium*	-	-	-	-	6 ± 2	10 ± 3	-
MA_AMQ_34	*Brevibacterium*	-	8 ± 3	-	-	-	9 ± 2	-
R2A_AXC_194	*Kocuria*	10 ± 4	-	-	14 ± 4	-	-	7 ± 2
MA_AMC_87	*Microbacterium*	-	-	-	6 ± 2	-	-	-
R2A_SPS_90	*Microbacterium*	-	-	-	4 ± 1	-	-	-
M1_AXT_2	*Microbulbifer*	-	-	-	5 ± 1	-	-	-
R2A_DAS_4	*Microbulbifer*	-	-	3 ± 1	-	-	-	-
AIA_TES_7	*Microbulbifer*	-	12 ± 4	-	-	-	-	-
R2A_CIS_91	*Micrococcus*	-	-	-	-	-	-	9 ± 2
SCA_CLR_217	*Micrococcus*	8 ± 3	-	-	-	-	-	-
M1_AXT_88	*Micrococcus*	11 ± 3	-	-	-	-	-	-
R2A_CRV_10	*Micromonospora*	-	-	13 ± 3	-	-	8 ± 2	-
SCA_HAS_11	*Micromonospora*	-	-	-	6 ± 2	6 ± 2	-	4 ± 1
M1_AXC_17	*Pseudoalteromonas*	-	-	-	-	8 ± 3	-	-
MA_AMC_15	*Pseudoalteromonas*	6 ± 2	-	-	10 ± 4	8 ± 4	-	4 ± 2
R2A_CIS_18	*Pseudoalteromonas*	-	-	-	-	-	7 ± 2	-
SCA_CRV_19	*Pseudoalteromonas*	-	-	-	-	6 ± 1	-	-
MA_AMQ_98	*Pseudomonas*	-	-	6 ± 2	-	-	-	4 ± 1
M1_AXT_131	*Pseudomonas*	-	12 ± 4	-	-	-	-	-
R2A_AXC_259	*Pseudomonas*	-	-	-	-	-	6 ± 2	-
SCA_CLR_279	*Pseudomonas*	-	-	9 ± 3	-	-	-	-
AIA_HAA_313	*Pseudomonas*	-	11 ± 4	-	-	9 ± 2	14 ± 4	-
MA_AMC_93	*Pseudovibrio*	-	-	-	3 ± 1	-	-	-
MA_AMC_33	*Pseudovibrio*	-	-	6 ± 3	-	-	-	-
MA_AMQ_100	*Pseudovibrio*	-	-	-	-	12 ± 4	-	-
MA_AXT_177	*Pseudovibrio*	13 ± 4	-	-	10 ± 3	-	-	-
MA_AXC_181	*Pseudovibrio*	13 ± 4	-	-	-	-	-	-
MA_CIS_184	*Pseudovibrio*	11 ± 4	-	-	-	8 ± 2	10 ± 3	-
MA_CIS_186	*Pseudovibrio*	-	-	5 ± 1	-	-	-	-
MA_CIS_195	*Pseudovibrio*	-	-	-	-	-	7 ± 1	-
MA_CRV_231	*Pseudovibrio*	-	8 ± 3	-	-	-	-	-
M1_DAS_236	*Pseudovibrio*	-	13 ± 3	-	-	7 ± 2	10 ± 3	-
M1_HAA_265	*Pseudovibrio*	-	-	-	-	-	-	5 ± 1
R2A_RHG_301	*Pseudovibrio*	-	-	6 ± 2	-	-	-	-
SCA_TES_374	*Pseudovibrio*	8 ± 2	-	-	14 ± 4	-	-	-
AIA_TEA_401	*Pseudovibrio*	-	15 ± 4	-	-	-	-	-
MA_AMQ_136	*Ruegeria*	-	-	6 ± 2	-	-	-	-
MA_AXT_139	*Ruegeria*	4 ± 1	-	-	7 ± 2	7 ± 2	13 ± 4	-
MA_CIS_145	*Ruegeria*	-	13 ± 4	-	-	7 ± 2	-	-
M1_DAS_153	*Ruegeria*	3 ± 1	-	-	-	-	-	-
R2A_SPV_381	*Ruegeria*	-	6 ± 2	-	-	-	-	-
MA_AMC_38	*Streptomyces*	-	-	3 ± 1	-	8 ± 4	-	-
MA_AMQ_39	*Streptomyces*	7 ± 2	-	-	5 ± 2	-	-	-
M1_AXT_41	*Streptomyces*	-	13 ± 3	-	-	-	-	-
M1_CIS_51	*Streptomyces*	-	-	-	6 ± 2	-	10 ± 3	-
R2A_CLR_53	*Streptomyces*	-	4 ± 1	-	4 ± 2	-	-	-
R2A_DAS_58	*Streptomyces*	-	-	-	-	-	8 ± 3	-
R2A_HAS_60	*Streptomyces*	-	-	-	-	7 ± 2	-	-
SCA_HAF_63	*Streptomyces*	-	9 ± 3	-	-	-	-	-
SCA_RHG_65	*Streptomyces*	-	-	-	7 ± 2	-	-	-
AIA_SPV_83	*Streptomyces*	-	6 ± 2	-	-	-	-	-
AIA_SPS_85	*Streptomyces*	-	-	-	-	-	4 ± 2	-
AIA_TEA_127	*Streptomyces*	-	-	-	-	5 ± 2	-	-
AIA_TES_126	*Streptomyces*	-	-	-	-	-	14 ± 4	-
AIA_TES_125	*Streptomyces*	-	4 ±1	-	7 ± 2	-	-	-
OLIGO_XES_128	*Streptomyces*	3 ± 1	-	-	-	-	-	-
SWA_XES_129	*Streptomyces*	-	8 ± 3	-	-	-	10 ± 3	-
AIA_SPV_84	*Streptomyces*	-	-	-	-	-	10 ± 3	7 ± 2
MA_AMC_44	*Vibrio*	-	-	-	-	5 ± 3	-	6 ± 2
R2A_SPS_117	*Vibrio*	-	-	-	-	-	8 ± 3	-
MA_AMQ_46	*Vibrio*	-	-	-	10 ± 3	-	-	-
AIA_TEA_122	*Vibrio*	-	-	-	-	4 ± 2	-	-
M1_RHG_114	*Vibrio*	-	-	-	-	-	-	4 ± 1
M1_HAA_109	*Vibrio*	-	-	12 ± 3	-	-	-	-
MA_CIS_55	*Vibrio*	-	-	-	-	5 ± 1	-	-
SCA_TES_120	*Vibrio*	11 ± 3	-	-	-	17 ± 3	-	-
M1_CLR_86	*Vibrio*	-	-	13 ± 4	-	-	-	-
Ampicillin		18 ± 3	20 ± 4	-	25 ± 4	21 ± 3	23 ± 3	na
Kanamycin		12 ± 2	17 ± 3	-	19 ± 3	18 ± 2	19 ± 2	na
Tetracycline		10 ± 2	19 ± 3	-	22 ± 3	15 ± 2	21 ± 3	na
Miconazole		na	na	na	na	na	na	17 ± 2

SE—*Salmonella enterica* ATCC 13076; EC—*Escherichia coli* ATCC 25922; PA—*Pseudomonas aeruginosa* ATCC 27853; SA—*Staphylococcus aureus* ATCC 25923; EF—*Enterococcus faecalis* ATCC 29212; BS—*Bacillus subtilis* ATCC 6633; CA—*Candida albicans* ATCC 10231; “-“: no inhibition; na: not applicable; values in table: mean ± SD of triplicate.

**Table 2:** Antimicrobial activity of the isolated compounds **1**,**2**,**3**, and **4**.

Reference Microorganisms	MIC (µg/mL)							
	1	2	3	4	Amp	Kan	Tet	Mic
**Gram negative bacteria**								
*E. coli* ATCC 25922	-	64	16	32	8	16	4	na
*S. enterica* ATCC 13076	-	-	>256	>256	16	64	32	na
*P. aeruginosa* ATCC 27853	-	8	64	64	>256	>256	64	na
*P. putida* MISR 71218	-	128	128	128	>256	64	64	na
*V. parahaemolyticus* MISR 21116	>256	128	64	128	64	32	32	na
*V. vulnificus* MISR 20716	-	-	128	256	32	64	32	na
*V. alginolyticus* MISR 30816	-	-	256	128	>256	32	64	na
**Gram positive bacteria**								
*E. faecalis* ATCC 29212	-	-	>256	>256	8	128	32	na
*S. aureus* ATCC 25923	>256	16	32	64	16	16	4	na
*B. subtilis* ATCC 6633	-	128	64	64	8	16	32	na
*B. cereus* MISR 12818	-	128	64	64	32	16	64	na
*Rhodococcus* sp. MISR 16518	-	32	64	128	16	8	32	na
**Fungi**								
*C. albicans* ATCC 10231	-	128	64	64	na	na	na	32
*A. niger* MISR 11215	-	64	64	64	na	na	na	8
*F. oxisporum* MISR 20415	>256	64	64	64	na	na	na	32
*R. solani* MISR 11115	>256	64	64	64	na	na	na	32

Amp—ampicillin; Kan—kanamycin; Tet—tetracycline; Mic—miconazole; “-“: no activity; na: not applicable.

## Data Availability

The 16S rRNA gene sequences of isolates are available in the NCBI database under accession numbers: MN703812–MN704271.

## Supplementary Materials

The following are available online at https://www.mdpi.com/article/10.3390/md19070353/s1, Table S1: Total number of colonies and genera from sponge samples and from media, Table S2: Betadisper and Adonis analyses of cultivable bacteria isolated from different culture media types, Table S3: Alpha diversity indexes of cultivable bacteria isolated from culture media, Table S4: Kruskal-Wallis chi-squared of alpha diversity indexes of cultivable bacteria isolated from culture media; Table S5: List of isolated strains from sponges; Tables S6–S9: ^1^H and ^13^C NMR data of the isolated compounds, Figures S1–S20: Spectra of the isolated compounds, Figures S21–S33: Effects of the isolated compounds on the indicator microorganisms.
