# Supplementary material for: Diversity and Antimicrobial Activity of Vietnamese Sponge-Associated Bacteria
Source: Mar Drugs. 2021 Jun 22;19(7):353. doi: 10.3390/md19070353 (PMC8307940; doi:10.3390/md19070353)
Supplement: Supplementary file 1 [file marinedrugs-19-00353-s001.zip › marinedrugs-1074873-supplementary.pdf]

marine drugs

Supplementary materials
Diversity and antimicrobial activity of Vietnamese sponge-associated bacteria

Ton That Huu Dat[1,2,*], Nguyen Thi Kim Cuc[1], Pham Viet Cuong[1], Hauke Smidt[2], Detmer Sipkema[2,*]

[1] Mientrung Institute for Scientific Research, Vietnam Academy of Science and Technology, 321 Huynh Thuc Khang, Hue city, Thua Thien Hue, Vietnam; kimcuc@imbc.vast.vn (N.T.K.C); pvcuong@misr.vast.vn (P.V.C)

[2] Laboratory of Microbiology, Wageningen University & Research, Stippeneng 4, 6708 WE Wageningen, The Netherlands; hauke.smidt@wur.nl (H.S)

* Correspondence: huudat.ton@wur.nl (T.T.H.D); detmer.sipkema@wur.nl (D.S); Tel.: +84-94-949-2778 (T.T.H.D); +31-317-483-113 (D.S)

marine drugs

MDPI

Table S1. Total number of colonies and genera from sponge samples and from media

Sample ID	Sponge sample	Culture media							Total colonies per sample	Number of genera per sample
		MA	M1	R2A	SCA	AIA	OLIGO	SWA		
AMC	*Amphimedon* sp. 1	20	3	0	0	0	2	0	**25**	15
AMQ	*Amphimedon* sp. 2	17	3	2	0	0	0	0	**22**	16
AXT	*Axinyssa* sp.	22	6	1	0	0	0	0	**29**	19
AXC	*Axos cliftoni*	11	6	3	0	0	2	0	**22**	16
CIS	*Cinachyrella schulzei*	20	6	5	2	0	0	0	**33**	21
CLR	*Clathria reinwardti*	9	4	4	6	0	0	0	**23**	15
CRV	*Haplosclerida* sp.	4	6	7	5	0	0	0	**22**	16
DAS	*Dactylospongia* sp.	0	17	6	2	4	0	0	**29**	20
HAS	*Halichondria* sp.	0	10	4	5	2	2	0	**23**	16
HAA	*Haliclona amboinensis*	3	11	4	3	2	0	2	**25**	16
HAF	*Haliclona fascigera*	0	11	3	4	0	4	0	**22**	16
NIS	*Niphatidae* sp.	2	8	4	3	3	3	0	**23**	14
RHG	*Rhabdastrella globostellata*	2	9	11	4	2	3	2	**33**	18
SPV	*Spheciospongia* sp.	0	2	15	3	5	5	2	**32**	21
SPS	*Spirastrella* sp.	0	0	8	6	3	3	2	**22**	15
TES	*Tedania* sp.	0	2	0	17	5	4	5	**33**	19
TEA	*Terpios aploos*	0	0	0	2	10	4	5	**21**	16
XES	*Xestospongia testudinaria*	0	0	2	0	14	6	12	**34**	19
**Total colonies per medium**		**110**	**104**	**79**	**62**	**50**	**38**	**30**	**473**	
**Total genera per medium**		**35**	**38**	**33**	**30**	**27**	**23**	**17**		**55**

marine drugs

MDPI

Table S2. Betadisper and Adonis analyses of cultivable bacteria isolated from different culture media types

	Betadisper				
	**df**	***Sum Sq***	***Mean Sq***	***F***	***p-value***
Culture media types	6	0.040115	0.0066858	1.7053	0.2068
Residuals	14	0.054889	0.0039206		
Total	20	0.076054			
	**Adonis**				
	**df**	***Sum Sq***	***Mean Sq***	R^2	***p-value***
Culture media types	6	1.7159	0.28598	0.46294	**0.001**
Residuals	14	1.9907	0.14219	0.53706	
Total	20	3.7066		1	

Table S3. Alpha diversity indexes of cultivable bacteria isolated from culture media

Media	S	H	J	In
MA	17.0±1.0	2.75±0.06	0.970±0.002	14.14±0.94
M1	17.0±1.0	2.75±0.06	0.972±0.001	14.42±0.86
R2A	17.7±1.5	2.84±0.09	0.989±0.002	16.51±1.54
SCA	16.0±1.0	2.75±0.06	0.993±0.003	15.34±0.80
AIA	13.0±1.0	2.55±0.07	0.994±0.002	12.60±0.87
OLIGO	10.7±1.5	2.35±0.15	0.997±0.001	10.52±1.52
SWA	8.0±1.0	2.06±0.13	0.995±0.001	7.84±1.00

Table S4. Kruskal-Wallis chi-squared of alpha diversity indexes of cultivable bacteria isolated from culture media

Index	df	*Kruskal-Wallis chi-squared*	*p-value*
Richness (*S*)	6	17.083	**0.0090**
Shannon (*H*)	6	16.639	**0.0107**
Evenness (*J*)	6	18.085	**0.0060**
Inverse Simpson (*In*)	6	17.522	**0.0075**

marine drugs

MDPI

Table S5. List of isolated strains from sponges

Isolate ID	Closest relative	Accession number	Identity (%)
MA_AMQ_1	Microbulbifer variabilis strain Ni-2088 16S ribosomal RNA, partial sequence	NR_041021.1	100
M1_AXT_2	Microbulbifer variabilis gene for 16S rRNA, partial sequence	AB266055.1	100
M1_CIS_3	Microbulbifer sp. NBRC 101763 gene for 16S rRNA, partial sequence	AB681553.1	100
R2A_DAS_4	Microbulbifer variabilis strain HNS025 16S ribosomal RNA gene, partial sequence	JN128259.1	100
R2A_HAF_5	Microbulbifer sp. HB09007 16S ribosomal RNA gene, partial sequence	FJ796077.1	100
AIA_SPV_6	Microbulbifer sp. CUA-860 16S ribosomal RNA gene, partial sequence	KJ732924.1	100
AIA_TES_7	Microbulbifer variabilis strain Mcap_H38 16S ribosomal RNA gene, partial sequence	KP640585.1	100
MA_AMC_8	Micromonospora sp. 1G68 16S ribosomal RNA gene, partial sequence	DQ994713.1	100
M1_AXT_9	Micromonospora sp. R1 16S ribosomal RNA gene, partial sequence	EU714258.1	100
R2A_CRV_10	Micromonospora sp. 173307 16S ribosomal RNA gene, partial sequence	EF544151.1	100
SCA_HAS_11	Micromonospora sp. S16-05 gene for 16S ribosomal RNA, partial sequence	LC011674.1	100
MA_HAA_12	Photobacterium sp. VibC-Oc-011 16S ribosomal RNA gene, partial sequence	KF577081.1	100
M1_HAF_13	Photobacterium gaetbulicola strain MMSZ2016-2 16S ribosomal RNA gene, partial sequence	KY174898.1	100
OLIGO_TEA_14	Photobacterium sp. VibC-Oc-011 16S ribosomal RNA gene, partial sequence	KF577081.1	100
MA_AMC_15	Pseudoalteromonas sp. strain E704-6 16S ribosomal RNA gene, partial sequence	MF975627.1	100
M1_AMQ_16	Pseudoalteromonas sp. PL85 gene for 16S ribosomal RNA, partial sequence	LC373532.1	100
M1_AXC_17	Pseudoalteromonas sp. strain E704-6 16S ribosomal RNA gene, partial sequence	MF975627.1	100
R2A_CIS_18	Pseudoalteromonas sp. PL85 gene for 16S ribosomal RNA, partial sequence	LC373532.1	100
SCA_CRV_19	Pseudoalteromonas sp. strain E707-9 16S ribosomal RNA gene, partial sequence	MF975632.1	100
AIA_HAS_20	Pseudoalteromonas sp. strain Bu15_18 16S ribosomal RNA gene, partial sequence	KY671152.1	100
OLIGO_NIS_21	Pseudoalteromonas sp. PL85 gene for 16S ribosomal RNA, partial sequence	LC373532.1	100
SWA_SPV_22	Pseudoalteromonas sp. An2 partial 16S rRNA gene, isolate An2	AJ551143.1	100

SWA_XES_23	Pseudoalteromonas sp. PL85 gene for 16S ribosomal RNA, partial sequence	LC373532.1	100
MA_AXT_24	Enterococcus faecium strain 12 16S ribosomal RNA gene, partial sequence	MH236313.1	99
MA_CIS_25	Enterococcus faecalis strain SLDL-211 16S ribosomal RNA gene, partial sequence	MH779826.1	99
M1_DAS_26	Enterococcus faecalis strain ZX2-1 16S ribosomal RNA gene, partial sequence	MG694661.1	100
R2A_HAF_27	Enterococcus faecalis strain ZX2-1 16S ribosomal RNA gene, partial sequence	MG694661.1	99
SCA_RHG_28	Enterococcus faecalis strain A3-1 16S ribosomal RNA gene, partial sequence	MH385351.1	99
SCA_TES_29	Enterococcus faecium strain HPRTGL206 16S ribosomal RNA gene, partial sequence	MH393916.1	99
MA_AXT_30	Brachybacterium paraconglomeratum strain SL-88 16S ribosomal RNA gene, partial sequence	KU894797.1	99
MA_AMC_31	Microbacterium sp. strain MB30 16S ribosomal RNA gene, partial sequence	MH699187.1	99
MA_AMC_32	Bacillus sp. CF9 16S ribosomal RNA gene, partial sequence	KP126830.1	99
MA_AMC_33	Pseudovibrio sp. strain S42 16S ribosomal RNA gene, partial sequence	KX989360.1	99
MA_AMQ_34	Brevibacterium sp. DN213_3F7 16S ribosomal RNA gene, partial sequence	KP769419.1	99
MA_AMC_35	Bacillus amyloliquefaciens strain B-4 chromosome, complete genome	CP031424.1	100
MA_AMC_36	Bacillus amyloliquefaciens strain B-4 chromosome, complete genome	CP031424.1	100
OLIGO_XES_37	Enterococcus faecalis strain A3-1 16S ribosomal RNA gene, partial sequence	MH385351.1	99
MA_AMC_38	Streptomyces sp. strain 762 16S ribosomal RNA gene, partial sequence	MG654687.1	100
MA_AMQ_39	Streptomyces sp. A01111 16S ribosomal RNA gene, partial sequence	KU382716.1	100
MA_AXT_40	Streptomyces sp. strain 762 16S ribosomal RNA gene, partial sequence	MG654687.1	100
M1_AXT_41	Streptomyces sp. strain 762 16S ribosomal RNA gene, partial sequence	MG654687.1	100
MA_AXT_42	Rhodococcus sp. strain A23 16S ribosomal RNA gene, partial sequence	MH688762.1	100
M1_DAS_43	Rhodococcus qingshengii strain B2 16S ribosomal RNA gene, partial sequence	KJ028076.1	100
MA_AMC_44	Vibrio sp. strain PrV0032 16S ribosomal RNA gene, partial sequence	MF948961.1	100
MA_AMC_45	Vibrio sp. strain PrV0032 16S ribosomal RNA gene, partial sequence	MF948961.1	100
MA_AMQ_46	Vibrio harveyi strain DZ141003 16S ribosomal RNA gene, partial sequence	KU245722.1	100
MA_AXT_47	Vibrio parahaemolyticus strain HH101313 16S ribosomal RNA gene, partial sequence	MG386398.1	100

MA_AXT_48	Vibrio mediterranei partial 16S rRNA gene, strain CAIM 1601	HF541964.1	100
MA_AXC_49	Vibrio sp. strain InS-286 16S ribosomal RNA gene, partial sequence	MF359517.1	99
M1_AXC_50	Streptomyces sp. strain 762 16S ribosomal RNA gene, partial sequence	MG654687.1	100
M1_CIS_51	Streptomyces sp. strain 762 16S ribosomal RNA gene, partial sequence	MG654687.1	100
R2A_CIS_52	Streptomyces sp. PN1018 16S ribosomal RNA gene, partial sequence	KF287177.1	100
R2A_CLR_53	Streptomyces sp. strain 762 16S ribosomal RNA gene, partial sequence	MG654687.1	100
MA_CIS_54	Vibrio harveyi strain DZ141003 16S ribosomal RNA gene, partial sequence	KU245722.1	100
MA_CIS_55	Vibrio harveyi strain vh10 16S ribosomal RNA gene, partial sequence	KJ700303.1	100
MA_AMC_56	Micrococcus luteus strain CGAPGPBBS-084 16S ribosomal RNA gene, partial sequence	KY495217.1	100
R2A_CRV_57	Streptomyces sp. strain 762 16S ribosomal RNA gene, partial sequence	MG654687.1	100
R2A_DAS_58	Streptomyces sp. strain 762 16S ribosomal RNA gene, partial sequence	MG654687.1	100
R2A_DAS_59	Streptomyces sp. strain 762 16S ribosomal RNA gene, partial sequence	MG654687.1	100
R2A_HAS_60	Streptomyces sp. strain 762 16S ribosomal RNA gene, partial sequence	MG654687.1	100
MA_CLR_61	Vibrio harveyi strain vh10 16S ribosomal RNA gene, partial sequence	KJ700303.1	100
SCA_HAA_62	Streptomyces sp. strain 762 16S ribosomal RNA gene, partial sequence	MG654687.1	100
SCA_HAF_63	Streptomyces sp. strain 762 16S ribosomal RNA gene, partial sequence	MG654687.1	100
SCA_NIS_64	Streptomyces sp. strain 762 16S ribosomal RNA gene, partial sequence	MG654687.1	100
SCA_RHG_65	Streptomyces sp. strain 762 16S ribosomal RNA gene, partial sequence	MG654687.1	100
MA_AMQ_66	Bacillus sp. V3X 16S ribosomal RNA gene, partial sequence	HQ727961.1	100
MA_AMQ_67	Bacillus sp. A5-11 16S ribosomal RNA gene, partial sequence	JX134465.1	100
MA_AMQ_68	Bacillus sp. (in: Bacteria) MH50 gene for 16S ribosomal RNA, partial sequence	LC373525.1	100
MA_AXT_69	Bacillus sp. (in: Bacteria) strain LPOC3 16S ribosomal RNA gene, partial sequence	MH412687.1	100
MA_AXT_70	Bacillus sp. (in: Bacteria) MH50 gene for 16S ribosomal RNA, partial sequence	LC373525.1	100
MA_AXT_71	Bacillus sp. (in: Bacteria) MH50 gene for 16S ribosomal RNA, partial sequence	LC373525.1	99
MA_AXT_72	Bacillus sp. (in: Bacteria) MH50 gene for 16S ribosomal RNA, partial sequence	LC373525.1	99

MA_AXC_73	Bacillus pumilus strain PPL-SSC2 16S ribosomal RNA gene, partial sequence	KM226935.1	99
MA_AXC_74	Bacillus aryabhattai strain DN67_5C7 16S ribosomal RNA gene, partial sequence	KP769436.1	100
MA_AXC_75	Bacillus aryabhattai strain DL 1 16S ribosomal RNA gene, partial sequence	MH321604.1	100
MA_CIS_76	Bacillus xiamenensis strain AKU6 16S ribosomal RNA gene, partial sequence	MF173074.1	100
MA_CIS_77	Bacillus megaterium strain As23 16S ribosomal RNA gene, partial sequence	KC633281.1	100
MA_CIS_78	Bacillus algicola strain HMF4132 16S ribosomal RNA gene, partial sequence	KT984000.1	100
MA_CIS_79	Bacillus horikoshii strain DSM 8719 16S ribosomal RNA, partial sequence	NR_040852.1	99
MA_CIS_80	Bacillus sp. (in: Bacteria) strain DE024 16S ribosomal RNA gene, partial sequence	KY860719.1	100
MA_CLR_81	Bacillus sp. DN88_4G3 16S ribosomal RNA gene, partial sequence	KP769435.1	100
SCA_RHG_82	Streptomyces sp. strain 762 16S ribosomal RNA gene, partial sequence	MG654687.1	99
AIA_SPV_83	Streptomyces sp. strain 762 16S ribosomal RNA gene, partial sequence	MG654687.1	99
AIA_SPV_84	Streptomyces sp. A01111 16S ribosomal RNA gene, partial sequence	KU382716.1	99
AIA_SPS_85	Streptomyces sp. A01111 16S ribosomal RNA gene, partial sequence	KU382716.1	99
M1_CLR_86	Vibrio alginolyticus strain HH101307 16S ribosomal RNA gene, partial sequence	MG386392.1	99
MA_AMC_87	Microbacterium sp. 8 SY-2016 partial 16S rRNA gene, strain 8	LT009507.1	100
M1_AXT_88	Micrococcus luteus strain Amic_3 16S ribosomal RNA gene, partial sequence	KX223364.1	100
SCA_HAS_89	Paracoccus sp. strain KSTI85 16S ribosomal RNA gene, partial sequence	KX989451.1	100
R2A_SPS_90	Microbacterium sp. 8 SY-2016 partial 16S rRNA gene, strain 8	LT009507.1	100
R2A_CIS_91	Micrococcus luteus strain PZ-51 16S ribosomal RNA gene, partial sequence	KX108885.1	100
MA_AMC_92	Pseudovibrio sp. strain S42 16S ribosomal RNA gene, partial sequence	KX989360.1	100
MA_AMC_93	Pseudovibrio sp. strain S42 16S ribosomal RNA gene, partial sequence	KX989360.1	100
MA_AMQ_94	Pseudovibrio sp. strain S42 16S ribosomal RNA gene, partial sequence	KX989360.1	100
MA_AXT_95	Arthrobacter agilis strain OAct554 16S ribosomal RNA gene, partial sequence	KJ812397.1	100
MA_CRV_96	Flavobacterium johnsoniae strain A3 16S ribosomal RNA gene, partial sequence	EU860081.1	100
M1_HAA_97	Flavobacterium johnsoniae strain A3 16S ribosomal RNA gene, partial sequence	EU860081.1	100

MA_AMQ_98	Pseudomonas sp. strain SAM89 16S ribosomal RNA gene, partial sequence	KX670902.1	99
MA_NIS_99	Acidovorax sp. DQS-01 16S ribosomal RNA gene, partial sequence	KP126996.1	99
MA_AMQ_100	Pseudovibrio sp. Pb3 16S ribosomal RNA gene, partial sequence	HQ647018.1	99
MA_AMQ_101	Pseudovibrio sp. strain S42 16S ribosomal RNA gene, partial sequence	KX989360.1	99
MA_AXT_102	Pseudovibrio sp. 2011SOCNI42 16S ribosomal RNA gene, partial sequence	KF582882.1	100
MA_AMC_103	Psychrobacter celer strain MT3 16S ribosomal RNA gene, partial sequence	MH213234.1	99
M1_CRV_104	Vibrio harveyi strain DZ141003 16S ribosomal RNA gene, partial sequence	KU245722.1	100
M1_DAS_105	Vibrio parahaemolyticus strain AP323 16S ribosomal RNA gene, partial sequence	MG575446.1	99
M1_DAS_106	Vibrio sp. strain InS-159 16S ribosomal RNA gene, partial sequence	MF359408.1	99
M1_HAS_107	Vibrio sp. strain PrV0032 16S ribosomal RNA gene, partial sequence	MF948961.1	100
M1_HAS_108	Vibrio sp. strain PrV0032 16S ribosomal RNA gene, partial sequence	MF948961.1	100
M1_HAA_109	Vibrio harveyi strain HQB1367 16S ribosomal RNA gene, partial sequence	MH044640.1	100
M1_HAF_110	Vibrio harveyi strain DZ141003 16S ribosomal RNA gene, partial sequence	KU245722.1	100
M1_NIS_111	Vibrio harveyi strain DZ141003 16S ribosomal RNA gene, partial sequence	KU245722.1	100
M1_NIS_112	Vibrio alginolyticus strain HH101307 16S ribosomal RNA gene, partial sequence	MG386392.1	99
M1_RHG_113	Vibrio alginolyticus strain HH101307 16S ribosomal RNA gene, partial sequence	MG386392.1	100
M1_RHG_114	Vibrio harveyi strain vh10 16S ribosomal RNA gene, partial sequence	KJ700303.1	100
M1_SPV_115	Vibrio harveyi strain vh10 16S ribosomal RNA gene, partial sequence	KJ700303.1	100
R2A_SPV_116	Vibrio mediterranei partial 16S rRNA gene, strain CAIM 1601	HF541964.1	100
R2A_SPS_117	Vibrio mediterranei partial 16S rRNA gene, strain CAIM 1601	HF541964.1	99
R2A_XES_118	Chromohalobacter sp. 582s-4 16S ribosomal RNA gene, partial sequence	GU371671.1	99
R2A_SPS_119	Vibrio mediterranei partial 16S rRNA gene, strain CAIM 1601	HF541964.1	99
SCA_TES_120	Vibrio shilonii strain NIOSSK079#72 16S ribosomal RNA gene, partial sequence	KY604834.1	99
SCA_TES_121	Vibrio alginolyticus strain hq-V214 16S ribosomal RNA gene, partial sequence	MH553019.1	100
AIA_TEA_122	Vibrio alginolyticus strain hq-V214 16S ribosomal RNA gene, partial sequence	MH553019.1	100

OLIGO_XES_123	Vibrio alginolyticus strain hq-V214 16S ribosomal RNA gene, partial sequence	MH553019.1	100
SWA_XES_124	Vibrio alginolyticus strain CX-73 16S ribosomal RNA gene, partial sequence	MH368393.1	100
AIA_TES_125	Streptomyces sp. A01111 16S ribosomal RNA gene, partial sequence	KU382716.1	99
AIA_TES_126	Streptomyces sp. A01111 16S ribosomal RNA gene, partial sequence	KU382716.1	100
AIA_TEA_127	Streptomyces sp. PN1018 16S ribosomal RNA gene, partial sequence	KF287177.1	100
OLIGO_XES_128	Streptomyces sp. PN1018 16S ribosomal RNA gene, partial sequence	KF287177.1	100
SWA_XES_129	Streptomyces sp. A01111 16S ribosomal RNA gene, partial sequence	KU382716.1	100
M1_DAS_130	Alcanivorax marinus strain NIOSSD020#62 16S ribosomal RNA gene, partial sequence	KY616238.1	100
M1_AXT_131	Pseudomonas mendocina strain PRM6 16S ribosomal RNA gene, partial sequence	JN544146.1	99
M1_SPV_132	Brucella melitensis strain AUH2 16S ribosomal RNA gene, partial sequence	EF187230.1	100
MA_AMC_133	Ruegeria sp. DN71_7G3 16S ribosomal RNA gene, partial sequence	KP769439.1	100
MA_AMC_134	Ruegeria sp. DN71_7G3 16S ribosomal RNA gene, partial sequence	KP769439.1	100
M1_AXC_135	Thalassobius aestuarii strain JC2049 16S ribosomal RNA gene, partial sequence	AY442178.2	99
MA_AMQ_136	Ruegeria sp. AU267A partial 16S rRNA gene, isolate AU267A	LN878642.1	99
MA_AMQ_137	Ruegeria sp. DN71_7G3 16S ribosomal RNA gene, partial sequence	KP769439.1	99
MA_AXT_138	Ruegeria sp. AU267A partial 16S rRNA gene, isolate AU267A	LN878642.1	99
MA_AXT_139	Ruegeria sp. DN71_7G3 16S ribosomal RNA gene, partial sequence	KP769439.1	99
MA_AXT_140	Ruegeria sp. DN71_7G3 16S ribosomal RNA gene, partial sequence	KP769439.1	100
OLIGO_AMC_141	Pseudoruegeria sabulilitoris strain GJMS-35 16S ribosomal RNA, partial sequence	NR_134142.1	99
MA_AXC_142	Ruegeria sp. AD4 16S ribosomal RNA gene, partial sequence	KT731244.1	99
MA_AXC_143	Ruegeria sp. AU267A partial 16S rRNA gene, isolate AU267A	LN878642.1	99
MA_CIS_144	Ruegeria sp. AU267A partial 16S rRNA gene, isolate AU267A	LN878642.1	99
MA_CIS_145	Ruegeria sp. AU267A partial 16S rRNA gene, isolate AU267A	LN878642.1	99
MA_CIS_146	Ruegeria sp. AU103_Ted2_MB partial 16S rRNA gene, isolate AU103_Ted2_MB	LN909258.1	100
MA_CIS_147	Ruegeria sp. AU448_Ted1_MB20SE partial 16S rRNA gene, isolate AU448_Ted1_MB20SE	LN909189.1	100

MA_CLR_148	Ruegeria sp. DN71_7G3 16S ribosomal RNA gene, partial sequence	KP769439.1	100
MA_CLR_149	Ruegeria sp. AU103_Ted2_MB partial 16S rRNA gene, isolate AU103_Ted2_MB	LN909258.1	99
M1_CRV_150	Ruegeria sp. AU267A partial 16S rRNA gene, isolate AU267A	LN878642.1	99
M1_CRV_151	Ruegeria sp. AU103_Ted2_MB partial 16S rRNA gene, isolate AU103_Ted2_MB	LN909258.1	100
MA_AXC_152	Roseovarius scapharcae strain MA4-5 16S ribosomal RNA, partial sequence	NR_145897.1	99
M1_DAS_153	Ruegeria sp. 2011SOCNI17 16S ribosomal RNA gene, partial sequence	KF582894.1	99
MA_AXT_154	Labrenzia sp. strain 1334-139 16S ribosomal RNA gene, partial sequence	KY770164.1	99
M1_DAS_155	Ruegeria sp. AU267A partial 16S rRNA gene, isolate AU267A	LN878642.1	99
M1_DAS_156	Ruegeria atlantica strain 58303 16S ribosomal RNA gene, partial sequence	KX418531.1	100
M1_HAS_157	Ruegeria atlantica strain DN83_2B6 16S ribosomal RNA gene, partial sequence	KP769432.1	100
M1_HAS_158	Ruegeria sp. strain EL46 16S ribosomal RNA gene, partial sequence	MF461362.1	99
M1_HAA_159	Ruegeria sp. R214E7 16S ribosomal RNA gene, partial sequence	FJ357642.1	100
M1_CIS_160	Labrenzia sp. strain 1334-139 16S ribosomal RNA gene, partial sequence	KY770164.1	99
M1_HAA_161	Ruegeria sp. DN71_7G3 16S ribosomal RNA gene, partial sequence	KP769439.1	100
R2A_DAS_162	Labrenzia sp. strain 1334-139 16S ribosomal RNA gene, partial sequence	KY770164.1	99
R2A_AXC_163	Janibacter melonis strain IHBB 11057 16S ribosomal RNA gene, partial sequence	KR085938.1	99
M1_AXC_164	Brachybacterium sp. strain BAB-2786 16S ribosomal RNA gene, partial sequence	MF188116.1	99
MA_AMQ_165	Leisingera aquimarina strain DN172_5F6 16S ribosomal RNA gene, partial sequence	KP769430.1	100
M1_CIS_166	Leisingera aquimarina strain DN172_5F6 16S ribosomal RNA gene, partial sequence	KP769430.1	100
M1_CIS_167	Roseovarius albus strain CECT 7450 16S ribosomal RNA, partial sequence	NR_134162.1	99
R2A_CRV_168	Roseovarius albus strain CECT 7450 16S ribosomal RNA, partial sequence	NR_134162.1	99
MA_CLR_169	Bacillus sp. (in: Bacteria) MH50 gene for 16S ribosomal RNA, partial sequence	LC373525.1	99
MA_CLR_170	Bacillus sp. (in: Bacteria) strain LPOC3 16S ribosomal RNA gene, partial sequence	MH412687.1	100
M1_CRV_171	Bacillus sp. (in: Bacteria) strain LPOC3 16S ribosomal RNA gene, partial sequence	MH412687.1	100
R2A_CRV_172	Brachybacterium sp. strain BAB-2786 16S ribosomal RNA gene, partial sequence	MF188116.1	99

M1_CRV_173	Bacillus sp. (in: Bacteria) strain LPOC3 16S ribosomal RNA gene, partial sequence	MH412687.1	99
M1_CRV_174	Bacillus sp. (in: Bacteria) strain LPOC3 16S ribosomal RNA gene, partial sequence	MH412687.1	99
M1_AXC_175	Brevibacterium sp. EP11 partial 16S rRNA gene, strain EP11	AM398220.1	99
SCA_CRV_176	Brevibacterium sp. EP11 partial 16S rRNA gene, strain EP11	AM398220.1	100
MA_AXT_177	Pseudovibrio sp. BC118 16S ribosomal RNA gene, partial sequence	KP319347.1	100
R2A_HAS_178	Rhodococcus qingshengii strain B2 16S ribosomal RNA gene, partial sequence	KJ028076.1	100
MA_AXT_179	Pseudovibrio sp. AB113 16S ribosomal RNA gene, partial sequence	KP319346.1	100
MA_RHG_180	Brevundimonas vesicularis strain G2-1-80 16S ribosomal RNA gene, partial sequence	KC494336.1	99
MA_AXC_181	Pseudovibrio sp. 4G02 16S ribosomal RNA gene, partial sequence	KP319311.1	100
MA_AXC_182	Pseudovibrio sp. BC118 16S ribosomal RNA gene, partial sequence	KP319347.1	100
MA_AMQ_183	Psychrobacter sp. 228(130zx) 16S rRNA gene, strain 228(130zx)	AM403661.1	99
MA_CIS_184	Pseudovibrio sp. BC118 16S ribosomal RNA gene, partial sequence	KP319347.1	100
MA_CIS_185	Pseudovibrio sp. BC118 16S ribosomal RNA gene, partial sequence	KP319347.1	100
MA_CIS_186	Pseudovibrio sp. BC118 16S ribosomal RNA gene, partial sequence	KP319347.1	99
MA_CIS_187	Pseudovibrio sp. AB111 16S ribosomal RNA gene, partial sequence	KP319345.1	100
MA_AXT_188	Escherichia coli strain PYK21 16S ribosomal RNA gene, partial sequence	MF582348.1	100
M1_TES_189	Rhodobacter sp. TUT3735 gene for 16S rRNA, partial sequence	AB251411.1	100
OLIGO_SPV_190	Anderseniella sp. strain Gy4 16S ribosomal RNA gene, partial sequence	KY885189.1	99
M1_HAF_191	Ruegeria sp. EK38B gene for 16S ribosomal RNA, partial sequence	LC053425.1	99
M1_HAF_192	Ruegeria sp. EK38B gene for 16S ribosomal RNA, partial sequence	LC053425.1	99
MA_AXC_193	Acinetobacter radioresistens strain DSSKY-A-001 chromosome, complete genome	CP027365.1	100
R2A_AXC_194	Kocuria rhizophila strain F2 16S ribosomal RNA gene, partial sequence	KM577162.1	99
MA_CIS_195	Pseudomonas sp. Snoq 117.2 16S ribosomal RNA gene, partial sequence	KF597277.1	99
MA_AMC_196	Staphylococcus epidermidis strain CSF41498 chromosome, complete genome	CP030246.1	99
M1_CLR_197	Acinetobacter radioresistens strain DSSKY-A-001 chromosome, complete genome	CP027365.1	99

M1_AXT_198	Mycobacterium sp. NB01 16S ribosomal RNA, partial sequence	AY188086.1	99
M1_DAS_199	Bacillus sp. NCIM5373 16S ribosomal RNA gene, partial sequence	KT291159.1	99
OLIGO_AXC_200	Nonlabens arenilitoris strain DN166_3E9 16S ribosomal RNA gene, partial sequence	KP769429.1	100
MA_AXT_201	Psychrobacter celer strain MT3 16S ribosomal RNA gene, partial sequence	MH213234.1	99
MA_AXT_202	Psychrobacter celer strain MT3 16S ribosomal RNA gene, partial sequence	MH213234.1	99
R2A_DAS_203	Roseovarius albus strain CECT 7450 16S ribosomal RNA, partial sequence	NR_134162.1	99
M1_DAS_204	Bacillus sp. DN88_4G3 16S ribosomal RNA gene, partial sequence	KP769435.1	100
MA_CLR_205	Pseudovibrio sp. 8H04 16S ribosomal RNA gene, partial sequence	KP319343.1	99
MA_CLR_206	Pseudovibrio sp. MaPt6 16S ribosomal RNA gene, partial sequence	JX436420.1	100
MA_AMC_207	Bradyrhizobium sp. strain MA108 16S ribosomal RNA gene, partial sequence	MH699180.1	99
M1_DAS_208	Bacillus megaterium strain As23 16S ribosomal RNA gene, partial sequence	KC633281.1	100
MA_AMQ_209	Bradyrhizobium viridifuturi strain SEMIA 690 16S ribosomal RNA, partial sequence	NR_145860.1	100
MA_AXT_210	Staphylococcus sp. M_Sw_oHS_07/11_10_1(2) 16S ribosomal RNA gene, partial sequence	KF777597.1	100
MA_CIS_211	Bradyrhizobium viridifuturi strain SEMIA 690 16S ribosomal RNA, partial sequence	NR_145860.1	99
MA_CLR_212	Pseudovibrio sp. 4G02 16S ribosomal RNA gene, partial sequence	KP319311.1	100
M1_AXC_213	Psychrobacter celer strain DN193_4B9 16S ribosomal RNA gene, partial sequence	KP769422.1	99
M1_NIS_214	Ruegeria sp. CECT 5091 16S ribosomal RNA gene, partial sequence	MH023307.1	99
M1_NIS_215	Ruegeria atlantica strain 2 DIS 4B 16S ribosomal RNA gene, partial sequence	MK224686.1	99
R2A_CIS_216	Psychrobacter celer strain MT3 16S ribosomal RNA gene, partial sequence	MH213234.1	99
SCA_CLR_217	Micrococcus luteus strain CGAPGPBBS-084 16S ribosomal RNA gene, partial sequence	KY495217.1	100
R2A_CLR_218	Psychrobacter celer strain DN193_4B9 16S ribosomal RNA gene, partial sequence	KP769422.1	100
M1_RHG_219	Ruegeria atlantica strain DN83_2B6 16S ribosomal RNA gene, partial sequence	KP769432.1	100
SCA_CLR_220	Janibacter melonis strain IHBB 11057 16S ribosomal RNA gene, partial sequence	KR085938.1	100
M1_DAS_221	Bacillus aryabhattai strain DN67_5C7 16S ribosomal RNA gene, partial sequence	KP769436.1	100
M1_HAS_222	Bacillus aryabhattai strain DN67_5C7 16S ribosomal RNA gene, partial sequence	KP769436.1	100

R2A_HAA_223	Brachybacterium sp. strain BAB-2786 16S ribosomal RNA gene, partial sequence	MF188116.1	100
R2A_CRV_224	Psychrobacter celer strain DN193_4B9 16S ribosomal RNA gene, partial sequence	KP769422.1	100
M1_HAS_225	Bacillus pumilus strain PPL-SSC2 16S ribosomal RNA gene, partial sequence	KM226935.1	99
R2A_CLR_226	Mycobacterium septicum strain D13 16S ribosomal RNA gene, partial sequence	AY772166.1	99
M1_HAS_227	Bacillus stratosphericus strain 907R 16S ribosomal RNA gene, partial sequence	MF083091.1	100
R2A_CLR_228	Leisingera aquimarina strain DN172_5F6 16S ribosomal RNA gene, partial sequence	KP769430.1	100
SCA_DAS_229	Leisingera aquimarina strain DN172_5F6 16S ribosomal RNA gene, partial sequence	KP769430.1	100
SCA_HAF_230	Brachybacterium sp. V589 16S ribosomal RNA gene, partial sequence	AF324202.2	100
MA_CRV_231	Pseudovibrio sp. BC118 16S ribosomal RNA gene, partial sequence	KP319347.1	100
MA_CRV_232	Pseudovibrio sp. 1D08 16S ribosomal RNA gene, partial sequence	KP319277.1	100
MA_CRV_233	Pseudovibrio sp. 7H04 16S ribosomal RNA gene, partial sequence	KP319334.1	99
M1_HAA_234	Bacillus sp. FS2 16S ribosomal RNA gene, partial sequence	GU084156.1	100
M1_DAS_235	Pseudovibrio sp. BC118 16S ribosomal RNA gene, partial sequence	KP319347.1	100
M1_DAS_236	Pseudovibrio sp. BC118 16S ribosomal RNA gene, partial sequence	KP319347.1	99
M1_DAS_237	Pseudovibrio sp. 1D08 16S ribosomal RNA gene, partial sequence	KP319277.1	100
M1_DAS_238	Pseudovibrio sp. 8H06 16S ribosomal RNA gene, partial sequence	KP319344.1	100
SWA_TES_239	Anderseniella sp. strain Gy4 16S ribosomal RNA gene, partial sequence	KY885189.1	99
M1_CLR_240	Staphylococcus saprophyticus strain P45L1B 16S ribosomal RNA gene, partial sequence	MH748281.1	100
MA_AXC_241	Sphingobium sp. RAC03 chromosome, complete genome	CP016456.1	99
R2A_DAS_242	Psychrobacter celer strain DN193_4B9 16S ribosomal RNA gene, partial sequence	KP769422.1	100
M1_HAA_243	Bacillus horikoshii strain DSM 8719 16S ribosomal RNA, partial sequence	NR_040852.1	99
M1_CIS_244	Sphingobium sp. RAC03 chromosome, complete genome	CP016456.1	99
R2A_CRV_245	Sphingobium sp. RAC03 chromosome, complete genome	CP016456.1	99
M1_HAA_246	Bacterium BAB-662 16S ribosomal RNA gene, partial sequence	KF913655.1	100
M1_RHG_247	Ruegeria sp. DN71_7G3 16S ribosomal RNA gene, partial sequence	KP769439.1	99

M1_HAS_248	Pseudovibrio sp. 8H04 16S ribosomal RNA gene, partial sequence	KP319343.1	100
M1_HAF_249	Bacillus sp. A5-11 16S ribosomal RNA gene, partial sequence	JX134465.1	100
M1_HAF_250	Bacillus algicola strain HMF4132 16S ribosomal RNA gene, partial sequence	KT984000.1	100
M1_HAS_251	Pseudovibrio sp. 8G09 16S ribosomal RNA gene, partial sequence	KP319341.1	100
SCA_NIS_252	Brachybacterium sp. strain BAB-2786 16S ribosomal RNA gene, partial sequence	MF188116.1	100
SCA_HAS_253	Sphingobium sp. RAC03 chromosome, complete genome	CP016456.1	100
OLIGO_RHG_254	Leisingera aquimarina strain DN172_5F6 16S ribosomal RNA gene, partial sequence	KP769430.1	100
R2A_HAS_255	Psychrobacter sp. RKEM 1523 16S ribosomal RNA gene, partial sequence	KU198810.1	100
SCA_HAS_256	Roseovarius albus strain CECT 7450 16S ribosomal RNA, partial sequence	NR_134162.1	99
AIA_DAS_257	Acinetobacter radioresistens strain DSSKY-A-001 chromosome, complete genome	CP027365.1	100
OLIGO_HAS_258	Acinetobacter radioresistens strain DSSKY-A-001 chromosome, complete genome	CP027365.1	100
R2A_AXC_259	Pseudomonas sp. C127 16S ribosomal RNA gene, partial sequence	DQ005892.1	99
OLIGO_SPV_260	Leisingera aquimarina strain DN172_5F6 16S ribosomal RNA gene, partial sequence	KP769430.1	100
SCA_HAA_261	Psychrobacter sp. RKEM 1523 16S ribosomal RNA gene, partial sequence	KU198810.1	100
AIA_HAA_262	Janibacter melonis strain IHBB 11057 16S ribosomal RNA gene, partial sequence	KR085938.1	100
M1_HAS_263	Pseudovibrio sp. 7H02 16S ribosomal RNA gene, partial sequence	KP319333.1	99
AIA_HAS_264	Brevibacterium linens strain Bv7 16S ribosomal RNA gene, partial sequence	MH493690.1	100
M1_HAA_265	Pseudovibrio sp. 7H02 16S ribosomal RNA gene, partial sequence	KP319333.1	100
M1_HAA_266	Pseudovibrio sp. 7H02 16S ribosomal RNA gene, partial sequence	KP319333.1	100
M1_HAA_267	Pseudovibrio sp. 7H02 16S ribosomal RNA gene, partial sequence	KP319333.1	100
M1_HAF_268	Pseudovibrio sp. 7H02 16S ribosomal RNA gene, partial sequence	KP319333.1	100
M1_HAF_269	Pseudovibrio sp. 8H04 16S ribosomal RNA gene, partial sequence	KP319343.1	99
M1_HAF_270	Pseudovibrio sp. BC118 16S ribosomal RNA gene, partial sequence	KP319347.1	99
M1_NIS_271	Pseudovibrio sp. 3H01 16S ribosomal RNA gene, partial sequence	KP319304.1	100
M1_HAF_272	Bacillus sp. (in: Bacteria) strain DE024 16S ribosomal RNA gene, partial sequence	KY860719.1	100

R2A_RHG_273	Ruegeria atlantica strain DN83_2B6 16S ribosomal RNA gene, partial sequence	KP769432.1	100
M1_NIS_274	Bacillus sp. DN88_4G3 16S ribosomal RNA gene, partial sequence	KP769435.1	100
R2A_NIS_275	Bacillus sp. 42-7 16S ribosomal RNA gene, partial sequence	FJ607051.1	100
R2A_NIS_276	Uncultured Bacillus sp. clone ACH-S-9 16S ribosomal RNA gene, partial sequence	KM873141.1	99
SCA_HAF_277	Uncultured Psychrobacter sp. clone CI45 16S ribosomal RNA gene, partial sequence	FJ695565.1	99
SCA_NIS_278	Psychrobacter celer strain DN193_4B9 16S ribosomal RNA gene, partial sequence	KP769422.1	100
SCA_CLR_279	Pseudomonas sp. strain Po_C2_3 16S ribosomal RNA gene, partial sequence	KY653040.1	100
SCA_CRV_280	Pseudomonas sp. CJ11064 16S ribosomal RNA gene, partial sequence	AF500211.1	99
AIA_NIS_281	Roseovarius albus strain CECT 7450 16S ribosomal RNA, partial sequence	NR_134162.1	99
AIA_RHG_282	Psychrobacter celer strain z1099 16S ribosomal RNA gene, partial sequence	KY582846.1	100
OLIGO_TES_283	Leisingera methylohalidivorans DSM 14336 strain MB2 16S ribosomal RNA, complete sequence	NR_121711.2	99
R2A_RHG_284	Bacillus sp. DN88_4G3 16S ribosomal RNA gene, partial sequence	KP769435.1	100
SCA_HAS_285	Pseudomonas sp. strain Po_C2_3 16S ribosomal RNA gene, partial sequence	KY653040.1	100
AIA_SPV_286	Roseovarius albus strain CECT 7450 16S ribosomal RNA, partial sequence	NR_134162.1	99
M1_NIS_287	Pseudovibrio sp. BC118 16S ribosomal RNA gene, partial sequence	KP319347.1	100
M1_NIS_288	Pseudovibrio axinellae strain Ad2 16S ribosomal RNA, partial sequence	NR_118255.1	99
M1_RHG_289	Pseudovibrio sp. 7H02 16S ribosomal RNA gene, partial sequence	KP319333.1	100
M1_RHG_290	Pseudovibrio sp. 7H02 16S ribosomal RNA gene, partial sequence	KP319333.1	99
R2A_RHG_291	Pseudovibrio axinellae strain Ad2 16S ribosomal RNA, partial sequence	NR_118255.1	99
R2A_RHG_292	Pseudovibrio axinellae strain Ad2 16S ribosomal RNA, partial sequence	NR_118255.1	99
MA_NIS_293	Aquimarina megaterium XH134 16S ribosomal RNA, partial sequence	NR_118560.1	100
M1_DAS_294	Bradyrhizobium sp. strain MA108 16S ribosomal RNA gene, partial sequence	MH699180.1	99
M1_HAA_295	Bradyrhizobium sp. strain MA108 16S ribosomal RNA gene, partial sequence	MH699180.1	99
R2A_RHG_296	Bacillus sp. (in: Bacteria) strain DE024 16S ribosomal RNA gene, partial sequence	KY860719.1	100
R2A_HAF_297	Uncultured Bradyrhizobium sp. clone PSC8 16S ribosomal RNA gene, partial sequence	GU293167.1	100

OLIGO_TEA_298	Tenacibaculum sp. MOLA 512 partial 16S rRNA gene, culture collection MOLA:512	AM990737.1	100
AIA_SPS_299	Brachybacterium paraconglomeratum strain CM22 16S ribosomal RNA gene, partial sequence	EU660345.1	99
R2A_RHG_300	Bradyrhizobium sp. strain MA108 16S ribosomal RNA gene, partial sequence	MH699180.1	100
R2A_RHG_301	Pseudovibrio sp. DN206_4B7 16S ribosomal RNA gene, partial sequence	KP769445.1	99
OLIGO_TEA_302	Brachybacterium sp. strain BAB-2786 16S ribosomal RNA gene, partial sequence	MF188116.1	100
AIA_NIS_303	Brevibacterium aurantiacum strain SMQ-1335 chromosome, complete genome	CP017150.1	100
OLIGO_SPV_304	Psychrobacter celer strain B_IV_3L25 16S ribosomal RNA gene, partial sequence	JF710994.1	100
R2A_RHG_305	Ruegeria atlantica strain 2 DIS 4B 16S ribosomal RNA gene, partial sequence	MK224686.1	99
R2A_SPV_306	Ruegeria sp. ws1-3 16S ribosomal RNA gene, partial sequence	JF719277.1	100
R2A_SPV_307	Ruegeria atlantica strain 2 DIS 4B 16S ribosomal RNA gene, partial sequence	MK224686.1	99
OLIGO_HAF_308	Sphingobium sp. RAC03 chromosome, complete genome	CP016456.1	100
M1_RHG_309	Aquimarina megaterium XH134 16S ribosomal RNA, partial sequence	NR_118560.1	100
R2A_RHG_310	Bacillus megaterium strain H-1 16S ribosomal RNA gene, partial sequence	KT273285.1	100
OLIGO_TES_311	Sphingobium sp. SA2 16S ribosomal RNA gene, partial sequence	KJ767657.1	99
R2A_RHG_312	Bacillus sp. DN88_4G3 16S ribosomal RNA gene, partial sequence	KP769435.1	99
AIA_HAA_313	Pseudomonas sp. strain Po_C2_3 16S ribosomal RNA gene, partial sequence	KY653040.1	99
OLIGO_SPS_314	Roseovarius sp. EF1C-B42 16S ribosomal RNA gene, partial sequence	KC545259.1	99
R2A_SPV_315	Pseudovibrio sp. DN206_4B7 16S ribosomal RNA gene, partial sequence	KP769445.1	99
R2A_SPV_316	Pseudovibrio sp. AB113 16S ribosomal RNA gene, partial sequence	KP319346.1	100
R2A_SPV_317	Uncultured Pseudovibrio sp. clone 0307_BHM1_39 16S ribosomal RNA gene, partial sequence	JQ515611.1	99
R2A_SPV_318	Uncultured Pseudovibrio sp. clone 0307_BHM1_39 16S ribosomal RNA gene, partial sequence	JQ515611.1	99
OLIGO_HAF_319	Pseudomonas stutzeri Hiro-3 gene for 16S ribosomal RNA, partial sequence	LC339941.1	99
R2A_RHG_320	Bacillus horikoshii strain DSM 8719 16S ribosomal RNA, partial sequence	NR_040852.1	99
MA_AMQ_321	Sphingomonas sp. DN81_6F7 16S ribosomal RNA gene, partial sequence	KP769438.1	99
R2A_SPV_322	Pseudovibrio sp. 8H04 16S ribosomal RNA gene, partial sequence	KP319343.1	99

SWA_SPS_323	Psychrobacter celer strain DN193_4B9 16S ribosomal RNA gene, partial sequence	KP769422.1	100
SWA_TES_324	Psychrobacter celer strain DN193_4B9 16S ribosomal RNA gene, partial sequence	KP769422.1	100
R2A_SPV_325	Ruegeria atlantica strain DN83_2B6 16S ribosomal RNA gene, partial sequence	KP769432.1	100
R2A_SPV_326	Bacillus sp. DN88_4G3 16S ribosomal RNA gene, partial sequence	KP769435.1	100
OLIGO_NIS_327	Pseudomonas sp. strain Po_C2_3 16S ribosomal RNA gene, partial sequence	KY653040.1	100
OLIGO_TES_328	Roseovarius albus strain CECT 7450 16S ribosomal RNA, partial sequence	NR_134162.1	99
SWA_TEA_329	Nonlabens arenilitoris strain DN166_3E9 16S ribosomal RNA gene, partial sequence	KP769429.1	100
SWA_TEA_330	Psychrobacter celer strain DN193_4B9 16S ribosomal RNA gene, partial sequence	KP769422.1	100
SWA_XES_331	Psychrobacter celer strain DN193_4B9 16S ribosomal RNA gene, partial sequence	KP769422.1	100
R2A_SPV_332	Bacillus sp. Ar1 16S ribosomal RNA gene, partial sequence	EU009208.1	99
OLIGO_RHG_333	Pseudomonas sp. strain Po_C2_3 16S ribosomal RNA gene, partial sequence	KY653040.1	100
AIA_XES_334	Aquimarina megaterium XH134 16S ribosomal RNA, partial sequence	NR_118560.1	99
OLIGO_SPS_335	Janibacter melonis strain IHBB 11057 16S ribosomal RNA gene, partial sequence	KR085938.1	100
SCA_CLR_336	Kocuria rhizophila strain RW13 16S ribosomal RNA gene, partial sequence	MH715224.1	99
R2A_SPV_337	Bacillus horikoshii strain DSM 8719 16S ribosomal RNA, partial sequence	NR_040852.1	99
R2A_SPV_338	Bacillus horikoshii strain DSM 8719 16S ribosomal RNA, partial sequence	NR_040852.1	99
SCA_SPV_339	Bacillus stratosphericus strain PgKB21 16S ribosomal RNA gene, partial sequence	MF979092.1	100
SCA_SPS_340	Bacillus aerophilus strain IHBB 11116 16S ribosomal RNA gene, partial sequence	KR085933.1	100
R2A_HAA_341	Mycobacterium sp. strain TM-B39 16S ribosomal RNA gene, partial sequence	MH698707.1	99
SCA_SPS_342	Bradyrhizobium sp. strain MA108 16S ribosomal RNA gene, partial sequence	MH699180.1	100
AIA_XES_343	Bradyrhizobium sp. strain MA108 16S ribosomal RNA gene, partial sequence	MH699180.1	99
SCA_SPS_344	Bacillus pumilus strain MB1 NIOT 2010 16S ribosomal RNA gene, partial sequence	HQ858057.1	99
SCA_SPS_345	Bacillus altitudinis strain CF13 16S ribosomal RNA gene, partial sequence	JX438703.1	100
SCA_TES_346	Bacillus algicola strain F12 16S ribosomal RNA, partial sequence	NR_029077.1	99
SCA_TES_347	Bacillus algicola strain HMF4132 16S ribosomal RNA gene, partial sequence	KT984000.1	100

SCA_TES_348	Bacillus algicola strain HMF4132 16S ribosomal RNA gene, partial sequence	KT984000.1	100
M1_CLR_349	Sphingomonas sp. DN81_6F7 16S ribosomal RNA gene, partial sequence	KP769438.1	100
SCA_CRV_350	Sphingomonas sp. DN81_6F7 16S ribosomal RNA gene, partial sequence	KP769438.1	100
R2A_SPS_351	Pseudovibrio sp. DN206_4B7 16S ribosomal RNA gene, partial sequence	KP769445.1	99
R2A_SPS_352	Pseudovibrio sp. DN206_4B7 16S ribosomal RNA gene, partial sequence	KP769445.1	99
M1_AMC_353	Algibacter lectus strain KMM 3902 16S ribosomal RNA, partial sequence	NR_025696.1	99
R2A_AMQ_354	Algibacter lectus strain KMM 3902 16S ribosomal RNA, partial sequence	NR_025696.1	99
M1_HAF_355	Kriegella aquimaris strain KMM 3942 16S ribosomal RNA gene, partial sequence	EU246690.1	99
R2A_SPS_356	Kriegella aquimaris strain KMM 3942 16S ribosomal RNA gene, partial sequence	EU246690.1	99
R2A_AMQ_357	Olleya namhaensis strain WT-MY15 16S ribosomal RNA, partial sequence	NR_109551.1	100
SCA_CLR_358	Olleya namhaensis strain WT-MY15 16S ribosomal RNA, partial sequence	NR_109551.1	100
SCA_DAS_359	Algibacter lectus strain KMM 3902 16S ribosomal RNA, partial sequence	NR_025696.1	100
AIA_XES_360	Algibacter lectus strain KMM 3902 16S ribosomal RNA, partial sequence	NR_025696.1	100
SCA_TES_361	Kriegella aquimaris strain KMM 3942 16S ribosomal RNA gene, partial sequence	EU246690.1	99
AIA_TEA_362	Kriegella aquimaris strain KMM 3942 16S ribosomal RNA gene, partial sequence	EU246690.1	99
R2A_NIS_363	Mycobacterium sp. strain DL90 16S ribosomal RNA gene, partial sequence	MH290160.3	99
SCA_RHG_364	Mycobacterium sp. strain DL90 16S ribosomal RNA gene, partial sequence	MH290160.3	100
SCA_TES_365	Bacillus sp. ADB_BD072 16S ribosomal RNA gene, partial sequence	KX027018.1	99
AIA_DAS_366	Micrococcus yunnanensis strain BAE13 16S ribosomal RNA gene, partial sequence	KP717416.1	99
SCA_SPS_367	Pseudovibrio sp. 8H04 16S ribosomal RNA gene, partial sequence	KP319343.1	99
SCA_TES_368	Pseudovibrio sp. AB113 16S ribosomal RNA gene, partial sequence	KP319346.1	100
R2A_HAS_369	Staphylococcus warneri strain FP12 16S ribosomal RNA gene, partial sequence	MH037141.1	100
R2A_HAA_370	Staphylococcus saprophyticus strain P45L1B 16S ribosomal RNA gene, partial sequence	MH748281.1	100
SCA_HAF_371	Staphylococcus saprophyticus strain P45L1B 16S ribosomal RNA gene, partial sequence	MH748281.1	100
SWA_XES_372	Sphingobium sp. strain dr-6 16S ribosomal RNA gene, partial sequence	MH173287.1	100

AIA_NIS_373	Sphingobium sp. strain dr-6 16S ribosomal RNA gene, partial sequence	MH173287.1	99
SCA_TES_374	Pseudovibrio sp. 8G10 16S ribosomal RNA gene, partial sequence	KP319342.1	100
AIA_SPV_375	Brevibacterium linens strain Bv7 16S ribosomal RNA gene, partial sequence	MH493690.1	100
OLIGO_TEA_376	Leisingera aquimarina strain DN172_5F6 16S ribosomal RNA gene, partial sequence	KP769430.1	100
SCA_SPV_377	Mycobacterium sp. strain 7402 16S ribosomal RNA gene, partial sequence	KX607208.1	100
AIA_TES_378	Bacillus oryzaecorticis strain KN-23 16S ribosomal RNA gene, partial sequence	KU933480.1	99
SWA_TEA_379	Brevibacterium linens strain Bv7 16S ribosomal RNA gene, partial sequence	MH493690.1	100
SWA_TEA_380	Roseovarius albus strain CECT 7450 16S ribosomal RNA, partial sequence	NR_134162.1	99
R2A_SPV_381	Ruegeria atlantica strain DN83_2B6 16S ribosomal RNA gene, partial sequence	KP769432.1	99
MA_AMC_382	Eudoraea chungangensis strain CAU 1296 16S ribosomal RNA, partial sequence	NR_1482991	99
AIA_RHG_383	Labrenzia sp. strain 1334-139 16S ribosomal RNA gene, partial sequence	KY770164.1	99
OLIGO_SPS_384	Labrenzia sp. A-3-20 16S ribosomal RNA gene, partial sequence	KT583494.1	99
R2A_SPS_385	Ruegeria atlantica strain DN83_2B6 16S ribosomal RNA gene, partial sequence	KP769432.1	100
R2A_SPS_386	Ruegeria atlantica strain DN83_2B6 16S ribosomal RNA gene, partial sequence	KP769432.1	99
SCA_TES_387	Ruegeria atlantica strain DN83_2B6 16S ribosomal RNA gene, partial sequence	KP769432.1	99
SCA_TES_388	Ruegeria atlantica strain DN83_2B6 16S ribosomal RNA gene, partial sequence	KP769432.1	99
SCA_TES_389	Ruegeria atlantica strain DN83_2B6 16S ribosomal RNA gene, partial sequence	KP769432.1	99
SCA_TES_390	Ruegeria atlantica strain DN83_2B6 16S ribosomal RNA gene, partial sequence	KP769432.1	99
AIA_TEA_391	Ruegeria atlantica strain DN83_2B6 16S ribosomal RNA gene, partial sequence	KP769432.1	99
OLIGO_TES_392	Labrenzia sp. A-3-20 16S ribosomal RNA gene, partial sequence	KT583494.1	99
SWA_XES_393	Labrenzia sp. A-3-20 16S ribosomal RNA gene, partial sequence	KT583494.1	99
M1_AMQ_394	Eudoraea chungangensis strain CAU 1296 16S ribosomal RNA, partial sequence	NR_1482991	99
R2A_AXT_395	Eudoraea chungangensis strain CAU 1296 16S ribosomal RNA, partial sequence	NR_1482991	99
SCA_TES_396	Pseudovibrio sp. Mexcell6.3B 16S ribosomal RNA gene, partial sequence	JX436424.1	100
SCA_TES_397	Pseudovibrio sp. Mexcell6.3B 16S ribosomal RNA gene, partial sequence	JX436424.1	100

SCA_TES_398	Pseudovibrio sp. 2011SOCNI18 16S ribosomal RNA gene, partial sequence	KF582862.1	99
SCA_TEA_399	Pseudovibrio sp. Mexcell6.3B 16S ribosomal RNA gene, partial sequence	JX436424.1	100
AIA_TEA_400	Pseudovibrio sp. 2011SOCNI18 16S ribosomal RNA gene, partial sequence	KF582862.1	100
AIA_TEA_401	Pseudovibrio sp. 2011SOCNI18 16S ribosomal RNA gene, partial sequence	KF582862.1	100
AIA_XES_402	Pseudovibrio sp. 2011SOCNI2 16S ribosomal RNA gene, partial sequence	KF582856.1	100
AIA_XES_403	Pseudovibrio sp. 2011SOCNI18 16S ribosomal RNA gene, partial sequence	KF582862.1	100
SCA_CIS_404	Eudoraea chungangensis strain CAU 1296 16S ribosomal RNA, partial sequence	NR_1482991	99
SCA_CLR_405	Eudoraea chungangensis strain CAU 1296 16S ribosomal RNA, partial sequence	NR_1482991	99
AIA_DAS_406	Eudoraea chungangensis strain CAU 1296 16S ribosomal RNA, partial sequence	NR_1482991	99
OLIGO_HAF_407	Eudoraea chungangensis strain CAU 1296 16S ribosomal RNA, partial sequence	NR_1482991	99
OLIGO_NIS_408	Eudoraea chungangensis strain CAU 1296 16S ribosomal RNA, partial sequence	NR_1482991	99
OLIGO_RHG_409	Eudoraea chungangensis strain CAU 1296 16S ribosomal RNA, partial sequence	NR_1482991	99
OLIGO_SPV_410	Eudoraea chungangensis strain CAU 1296 16S ribosomal RNA, partial sequence	NR_1482991	99
AIA_XES_411	Pseudovibrio sp. 2011SOCNI18 16S ribosomal RNA gene, partial sequence	KF582862.1	100
AIA_TEA_412	Ruegeria sp. DN71_7G3 16S ribosomal RNA gene, partial sequence	KP769439.1	99
OLIGO_XES_413	Pseudovibrio sp. 2011SOCNI18 16S ribosomal RNA gene, partial sequence	KF582862.1	100
AIA_XES_414	Ruegeria sp. 2011SOCNI17 16S ribosomal RNA gene, partial sequence	KF582894.1	100
AIA_XES_415	Ruegeria sp. 2011SOCNI17 16S ribosomal RNA gene, partial sequence	KF582894.1	100
SWA_RHG_416	Micrococcus luteus strain FC1737 16S ribosomal RNA gene, partial sequence	MH665979.1	100
SWA_SPV_417	Micrococcaceae bacterium HW-2 16S ribosomal RNA gene, partial sequence	KM885169.1	100
SWA_TES_418	Micrococcus luteus strain SSW15 16S ribosomal RNA gene, partial sequence	KU922513.1	100
SWA_XES_419	Pseudovibrio sp. 2011SOCNI18 16S ribosomal RNA gene, partial sequence	KF582862.1	100
SWA_XES_420	Micrococcus sp. strain T3211-1-1 16S ribosomal RNA gene, partial sequence	MG254804.1	100
AIA_XES_421	Ruegeria sp. 2011SOCNI17 16S ribosomal RNA gene, partial sequence	KF582894.1	100
OLIGO_XES_422	Ruegeria sp. 2011SOCNI17 16S ribosomal RNA gene, partial sequence	KF582894.1	100

SWA_SPS_423	Eudoraea chungangensis strain CAU 1296 16S ribosomal RNA, partial sequence	NR_1482991	99
SWA_TES_424	Eudoraea chungangensis strain CAU 1296 16S ribosomal RNA, partial sequence	NR_1482991	99
SWA_TEA_425	Eudoraea chungangensis strain CAU 1296 16S ribosomal RNA, partial sequence	NR_1482991	99
SWA_XES_426	Eudoraea chungangensis strain CAU 1296 16S ribosomal RNA, partial sequence	NR_1482991	99
MA_CIS_427	Cellulosimicrobium cellulans partial 16S rRNA gene, strain PrF	HG000003.1	99
M1_RHG_428	Cellulosimicrobium cellulans partial 16S rRNA gene, strain PrF	HG000003.1	99
SCA_SPS_429	Staphylococcus sp. 29-3pA 16S ribosomal RNA gene, partial sequence	KU060115.1	99
OLIGO_SPV_430	Pseudomonas plecoglossicida strain SLr02 16S ribosomal RNA gene, partial sequence	MG755757.1	100
M1_AMC_431	Burkholderia sp. A-3 gene for 16S ribosomal RNA, partial sequence	AB694974.1	99
R2A_CRV_432	Burkholderia sp. strain MR5 16S ribosomal RNA gene, partial sequence	MG674315.1	100
R2A_NIS_433	Burkholderia sp. strain JGSB06 16S ribosomal RNA gene, partial sequence	MF285777.1	100
AIA_DAS_434	Uncultured Chryseobacterium sp. clone 6 16S ribosomal RNA gene, partial sequence	EU026431.1	99
M1_AMQ_435	Ralstonia sp. strain CWS_Control 16S ribosomal RNA gene, partial sequence	KX025096.1	99
R2A_CIS_436	Uncultured Ralstonia sp. clone EC34BC01 16S ribosomal RNA gene, partial sequence	JN032362.1	99
SCA_SPV_437	Burkholderia sp. strain JGSB06 16S ribosomal RNA gene, partial sequence	MF285777.1	100
AIA_TEA_438	Bacillus sp. strain MML5312 16S ribosomal RNA gene, partial sequence	MF687522.1	99
AIA_TES_439	Burkholderia sp. strain MR5 16S ribosomal RNA gene, partial sequence	MG674315.1	100
AIA_SPS_440	Kocuria rosea partial 16S rRNA gene, strain PrD	HG000002.1	100
SWA_HAA_441	Acinetobacter indicus strain SGAir0564 chromosome, complete genome	CP024620.1	100
R2A_HAA_442	Exiguobacterium mexicanum strain MSSRFS9 16S ribosomal RNA gene, partial sequence	KF471138.1	99
AIA_XES_443	Staphylococcus saprophyticus strain FDAARGOS_168 chromosome, complete genome	CP014113.2	100
OLIGO_HAF_444	Curtobacterium sp. strain B4 16S ribosomal RNA gene, partial sequence	MG234436.1	100
SWA_TES_445	Pseudomonas stutzeri partial 16S rRNA gene, strain 24a75	AJ312229.1	99
AIA_XES_446	Acidovorax sp. DQS-01 16S ribosomal RNA gene, partial sequence	KP126996.1	100
M1_RHG_447	Escherichia coli strain F3-1-9 16S ribosomal RNA gene, partial sequence	KX349997.1	100

R2A_SPV_448	Escherichia coli strain F3-1-9 16S ribosomal RNA gene, partial sequence	KX349997.1	100
SCA_TEA_449	Escherichia coli strain 2452 chromosome, complete genome	CP031833.1	100
SWA_XES_450	Ruegeria atlantica strain DN83_2B6 16S ribosomal RNA gene, partial sequence	KP769432.1	99
AIA_TEA_451	Bacillus sp. (in: Bacteria) strain CL42 16S ribosomal RNA gene, partial sequence	MH605371.1	100
MA_CIS_452	Halobacillus sp. strain Q-13 16S ribosomal RNA gene, partial sequence	MF035455.1	100
AIA_TEA_453	Uncultured Bacillus sp. clone TOPO4 16S ribosomal RNA gene, partial sequence	KY963640.1	99
AIA_XES_454	Bacillus sp. strain pc-3w 16S ribosomal RNA gene, partial sequence	MF993476.1	100
SCA_CRV_455	Ralstonia pickettii strain VIT-SRM1 16S ribosomal RNA gene, partial sequence	KJ716446.1	99
AIA_XES_456	Bacillus altitudinis strain TR38T1 16S ribosomal RNA gene, partial sequence	KF737162.1	100
SWA_XES_457	Pseudomonas sp. BBAL-02d 16S ribosomal RNA gene, partial sequence	FJ217180.1	99
AIA_XES_458	Bacillus sp. (in: Bacteria) strain T16-2-1 16S ribosomal RNA gene, partial sequence	MG254766.1	100
OLIGO_XES_459	Bacillus sp. (in: Bacteria) strain Firmi-75 16S ribosomal RNA gene, partial sequence	MH683164.1	99
SWA_XES_460	Bacillus infantis NRRL B-14911, complete genome	CP006643.1	100

marine drugs

MDPI

Table S6. ^{1}H and ^{13}C NMR data of Cyclo-(L-Pro-L-Tyr) (**1**)

Position	#δ_C a	δ_C a,b	δ_H a,c mult (J = Hz)
1	165.6	166.9	-
2	-	-	-
3	44.5	45.9	3.37 m<br>3.57 m
4	21.3	22.7	1.82 (2H, m)
5	28.0	29.3	2.11 (2H, m)
6	58.7	60.0	4.07 (m)
7	169.4	170.8	-
8	-	-	-
9	56.5	57.9	4.37 (m)
10	36.3	37.6	3.07 (2H, m)
1'	126.4	127.6	-
2', 6'	130.7	132.1	7.05 (2H, d, 8.5)
3', 5'	114.8	116.2	6.72 (2H, d, 8.5)
4'	156.3	157.6	-

Measured in a)CD_3OD, b)125 MHz, c) 500 MHz, #δ_C of Cyclo-(L-Pro-L-Tyr) [1]

Table S7. ^{1}H and ^{13}C NMR data of macrolactin A (**2**)

Position	$^{\#}\delta_C{}^a$	$\delta_C{}^{a,b}$	$\delta_H{}^{a,c}$ mult (*J* = Hz)
1	166.83	168.0	
2	118.15	117.9	5.56 (m)
3	145.12	144.9	6.65 (t, 11.5)
4	130.42	130.2	7.23 (m)
5	142.34	142.1	6.19 (m)
6	43.02	42.8	2.44 (2H, m)
7	72.37	72.3	4.28 (m)
8	137.71	137.5	5.77 (dd, 6.0, 15.0)
9	126.10	125.9	6.58 (dd, 11.0, 15.0)
10	131.56	131.3	6.13 (m)
11	128.54	128.4	5.55 (m)
12	36.67	36.4	2.34 (m)<br>2.50 (m)
13	69.37	69.2	3.87 (m)
14	44.07	43.9	1.63 (2H, m)
15	69.97	69.8	4.32 (m)
16	135.40	135.2	5.57 (m)
17	131.36	131.2	6.10 (m)
18	131.87	131.7	6.05 (m)
19	135.29	135.1	5.66 (m)
20	25.81	25.6	1.52 (2H, m)
21	33.14	32.9	2.12 (m)<br>2.20 (m)
22	36.17	36.0	1.59 (m)<br>1.65 (m)
23	72.49	72.2	5.02 (m)
24	20.27	20.1	1.27 (3H, d, 6.5)

Measured in $^{a)}$ *CD_3OD,* $^{b)}$ *500 MHz,* $^{c)}$ *125 MHz,* $^{\#}\delta_C$ *of macrolactin A [2]*

Table S8. ^{1}H and ^{13}C NMR data of macrolactin H (**3**)

Position	$^{\#}\delta_C{}^{d}$	$\delta_C{}^{a,b}$	$\delta_H{}^{a,c}$ mult (*J* = Hz)
1	166.3	166.3	-
2	117.9	118.0	5.55 (m)
3	143.7	144.8	6.65 (m)
4	130.1	130.4	5.59 (m)
5	139.9	141.7	6.17 (m)
6	41.5	42.5	2.45 (2H, m)
7	70.6	72.0	4.98 (m)
8	135.9	137.2	5.76 (m)
9	126.0	126.1	6.75 (m)
10	130.7	131.0	5.63 (m)
11	134.2	134.7	5.46 (m)
12	36.1	36.5	1.58 (m)<br>2.23 (m)
13	69.8	69.7	4.32 (m)
14	41.5	44.6	1.59 (2H, m)
15	70.6	70.2	4.26 (m)
16	128.1	128.3	5.55 (m)
17	130.4	131.7	5.65 (m)
18	32.1	32.9	2.08 (2H, m)
19	24.7	25.9	1.40 (2H, m)
20	35.0	36.6	1.65 (2H, m)
21	70.8	70.8	4.28 (m)
22	19.6	20.1	1.27 (3H, d, 6.5)

Measured in $^{a)}$ *CD_3OD,* $^{b)}$*125 MHz,* $^{c)}$ *500 MHz,* $^{d)}$ *Benzen-d_6,* $^{\#}\delta_C$ *of macrolactin H* [3]

Table S9. ^{1}H and ^{13}C NMR data of 15,17-epoxy-16-hydroxy macrolactin A (**4**)

Position	$^{\#}\delta_C{}^{a,d}$	$\delta_C{}^{a,b}$	$\delta_H{}^{a,c}$ mult (J = Hz)
1	168.3	168.2	
2	118.3	118.2	5.54 (d, 11.5)
3	144.8	144.6	6.67 (t, 11.5)
4	130.7	130.6	7.25 (m)
5	141.6	141.4	6.27 (m)
6	42.9	42.7	2.51 (m)<br>2.42 (m)
7	73.2	73.1	4.27 (m)
8	136.4	136.2	5.64 (m)
9	127.9	127.7	6.56 (dd, 11.0, 15.0)
10	131.5	131.4	6.07 (t, 11.0)
11	129.7	129.5	5.46 (m)
12	35.3	35.1	2.57 (m)<br>2.13 (m)
13	76.1	76.0	3.46 (m)
14	40.9	40.8	1.99 (m)<br>1.40 (m)
15	74.0	73.9	3.54 (m)
16	77.8	77.7	2.94 (t, 9.0)
17	80.3	80.2	3.46 (m)
18	129.2	129.1	5.67 (m)
19	132.3	132.2	5.70 (m)
20	34.6	34.4	2.11 (m)<br>2.05 (m)
21	26.8	26.6	1.52 (2H, m)
22	37.0	36.8	1.67 (2H, m)
23	73.1	72.9	4.98 (m)
24	20.0	19.8	1.28 (3H, d, 6.0)

Measured in [a)] CD_3OD, [b)] 125 MHz, [c)] 500 MHz, $^{\#}\delta_C$ of 15,17-epoxy-16-hydroxy macrolactin A [4]

Figure S1-S20. Spectra of the isolated compounds

Figure S1. ESI-MS spectrum of Cyclo-(L-Pro-L-Tyr) (**1**)
Figure S2. ^{1}H NMR spectrum of Cyclo-(L-Pro-L-Tyr) (**1**)
Figure S3. ^{13}C NMR spectrum of Cyclo-(L-Pro-L-Tyr) (**1**)
Figure S4. HSQC spectrum of Cyclo-(L-Pro-L-Tyr) (**1**)
Figure S5. HMBC spectrum of Cyclo-(L-Pro-L-Tyr) (**1**)
Figure S6. ESI-MS spectrum of macrolactin A (**2**)
Figure S7. ^{1}H NMR spectrum of macrolactin A (**2**)
Figure S8. ^{13}C NMR spectrum of macrolactin A (**2**)
Figure S9. HSQC spectrum of macrolactin A (**2**)
Figure S10. HMBC spectrum of macrolactin A (**2**)
Figure S11. ESI-MS spectrum of macrolactin H (**3**)
Figure S12. ^{1}H NMR spectrum of macrolactin H (**3**)
Figure S13. ^{13}C NMR spectrum of macrolactin H (**3**)
Figure S14. HSQC spectrum of macrolactin H (**3**)
Figure S15. HMBC spectrum of macrolactin H (**3**)
Figure S16. ESI-MS spectrum of 15,17-epoxy-16-hydroxy macrolactin A (**4**)
Figure S17. ^{1}H NMR spectrum of 15,17-epoxy-16-hydroxy macrolactin A (**4**)
Figure S18. ^{13}C NMR spectrum of 15,17-epoxy-16-hydroxy macrolactin A (**4**)
Figure S19. HSQC spectrum of 15,17-epoxy-16-hydroxy macrolactin A (**4**)
Figure S20. HMBC spectrum of 15,17-epoxy-16-hydroxy macrolactin A (**4**)

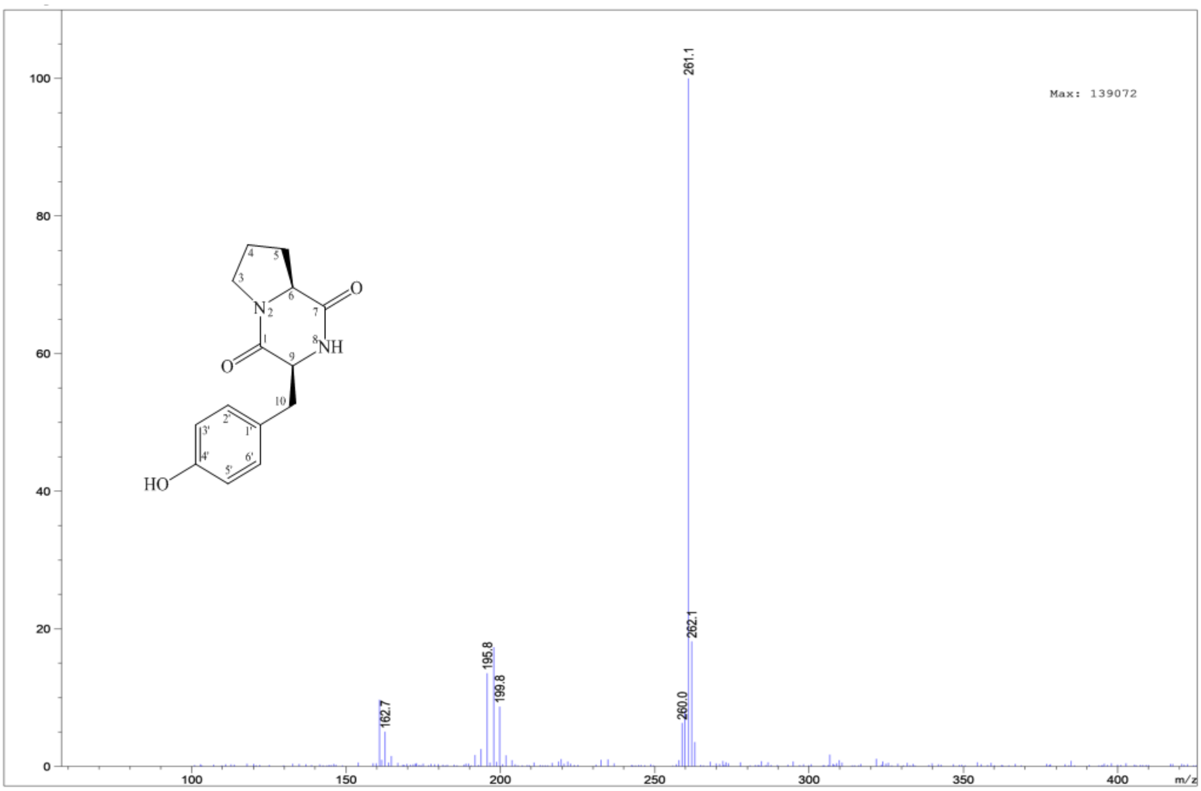

Figure S1. ESI-MS spectrum of Cyclo-(L-Pro-L-Tyr) (**1**)

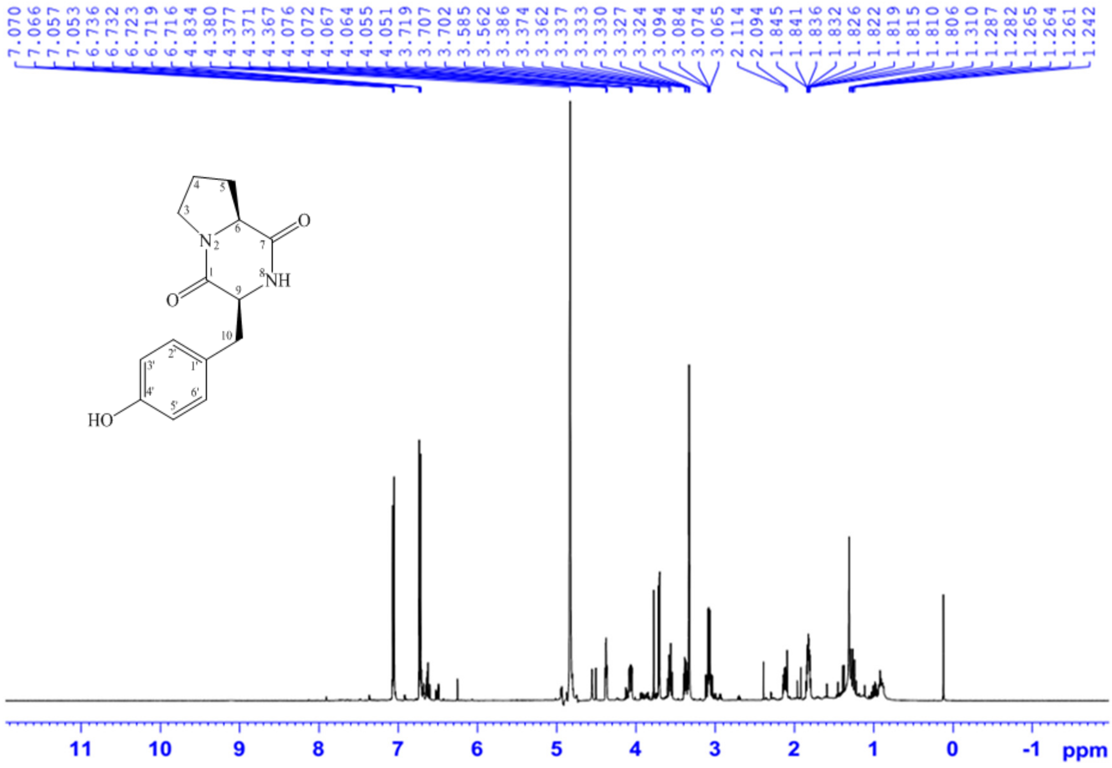

Figure S2. ^{1}H NMR spectrum of Cyclo-(L-Pro-L-Tyr) (**1**)

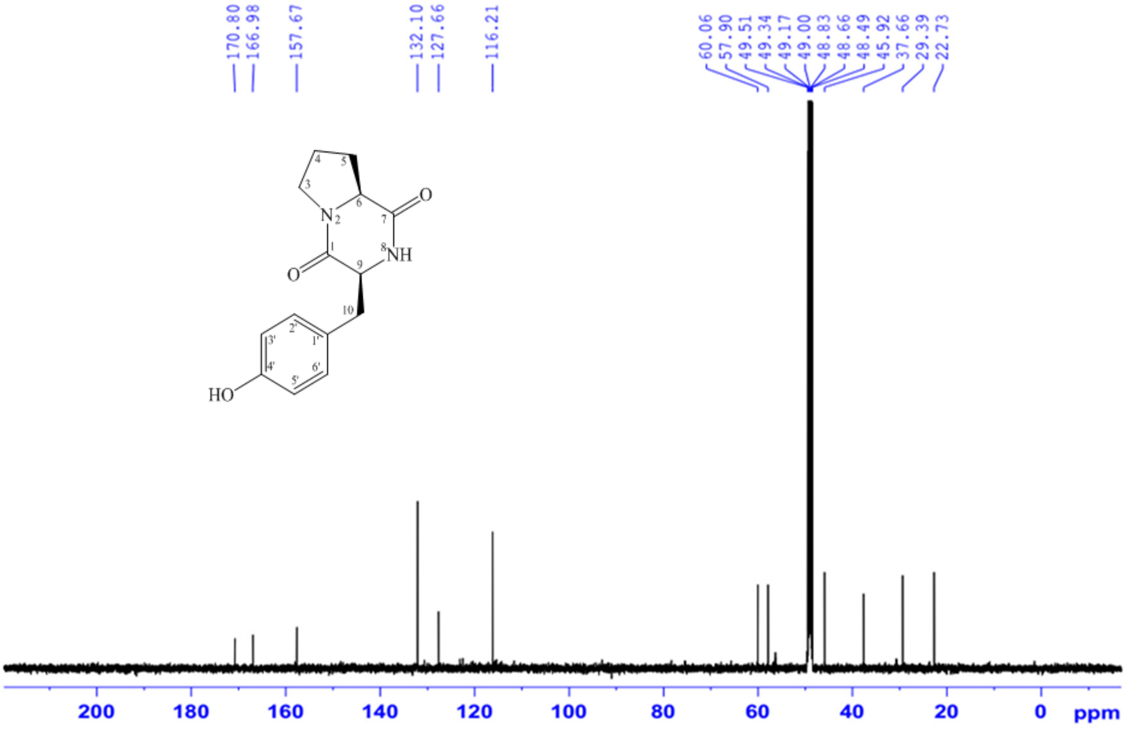

Figure S3. ^{13}C NMR spectrum of Cyclo-(L-Pro-L-Tyr) (**1**)

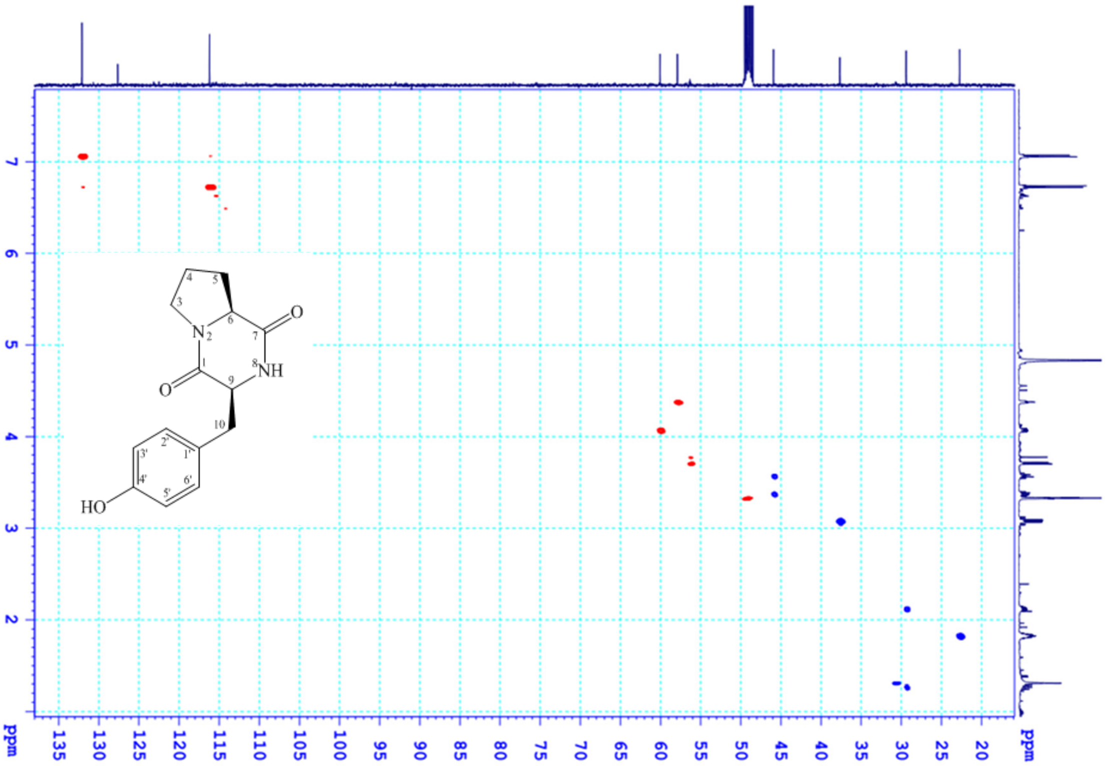

Figure S4. HSQC spectrum of Cyclo-(L-Pro-L-Tyr) (**1**)

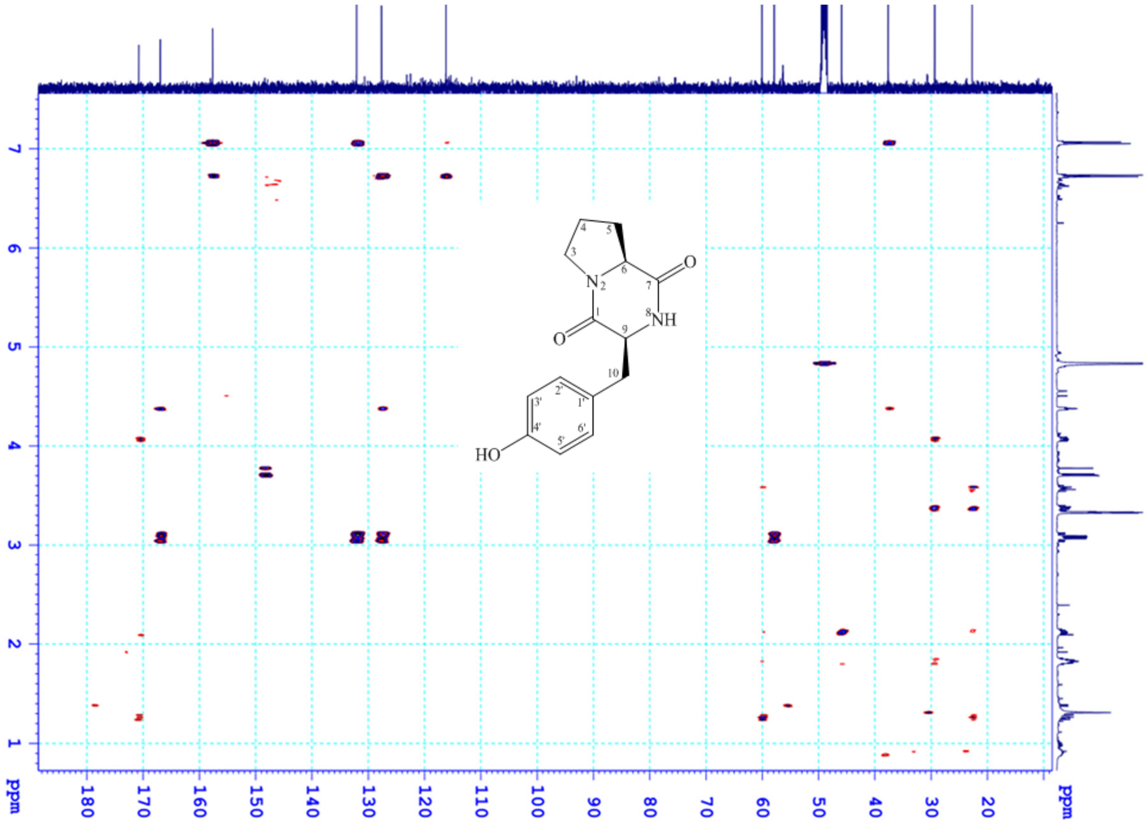

Figure S5. HMBC spectrum of Cyclo-(L-Pro-L-Tyr) (**1**)

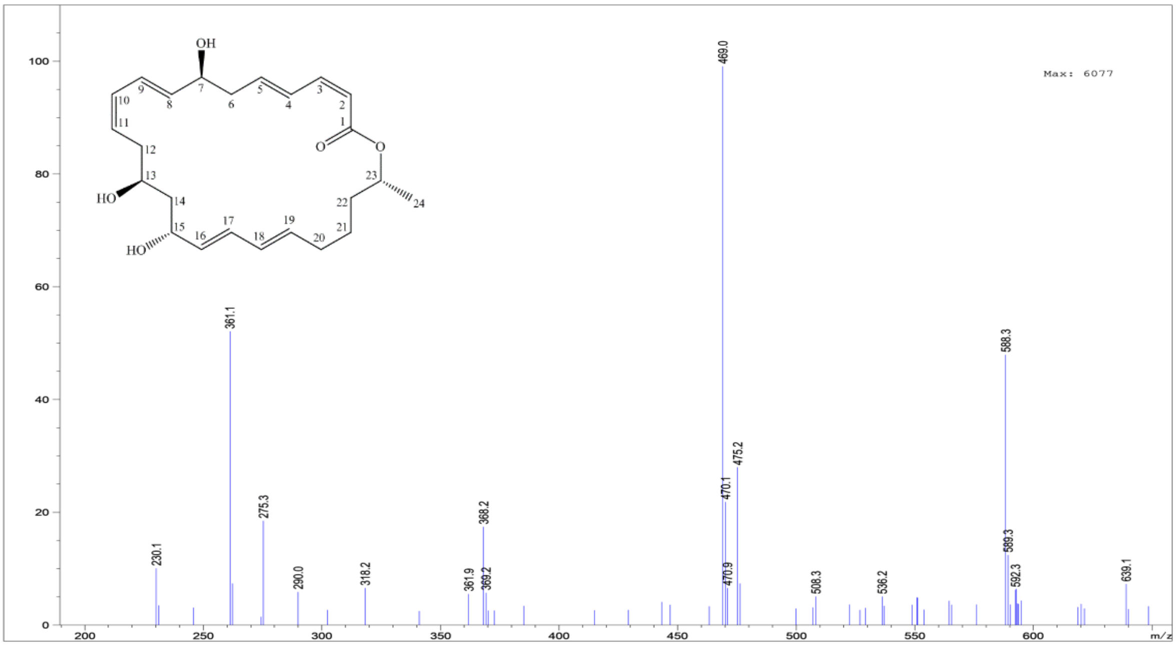

Figure S6. ESI-MS spectrum of macrolactin A (**2**)

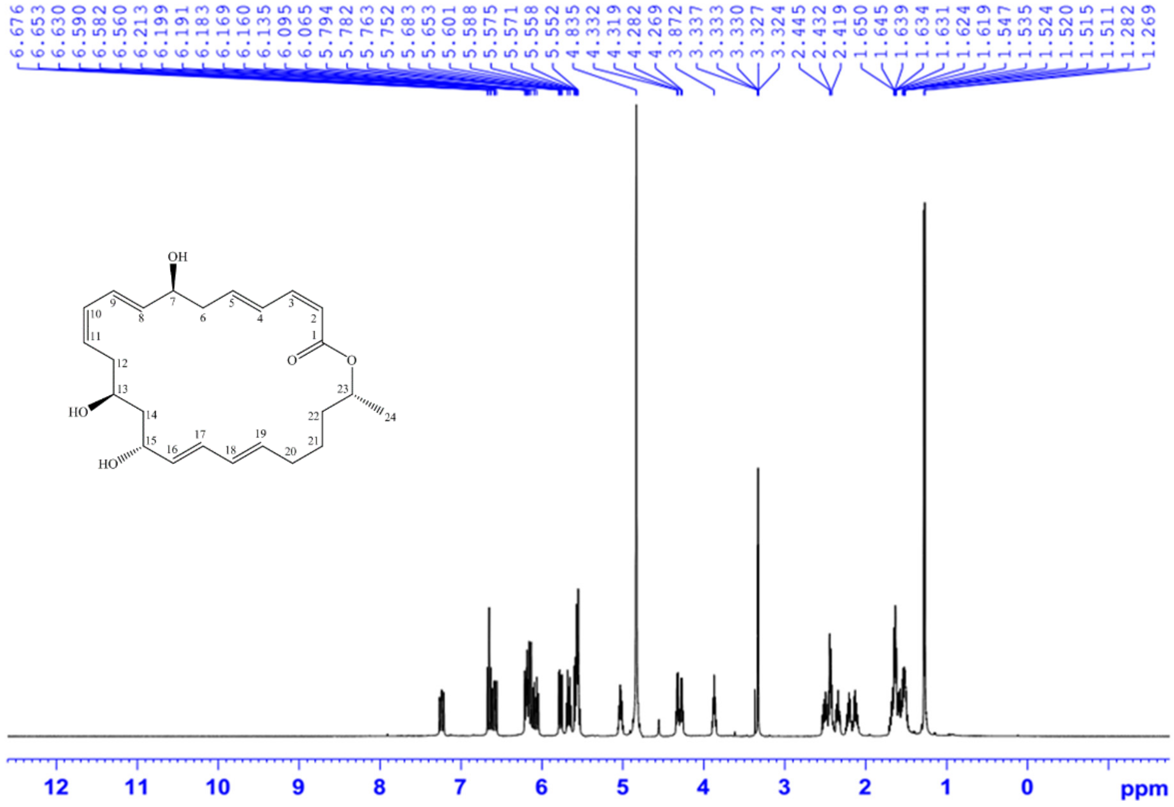

Figure S7. ^{1}H NMR spectrum of macrolactin A (**2**)

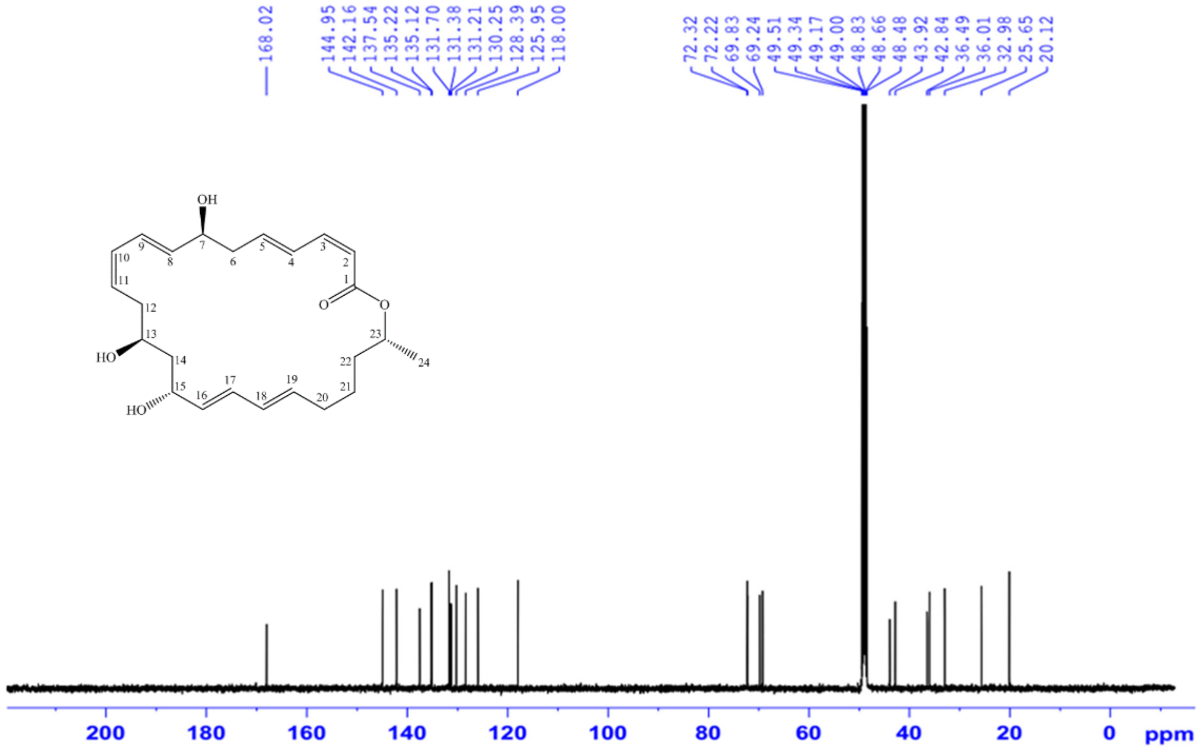

Figure S8. ^{13}C NMR spectrum of macrolactin A (**2**)

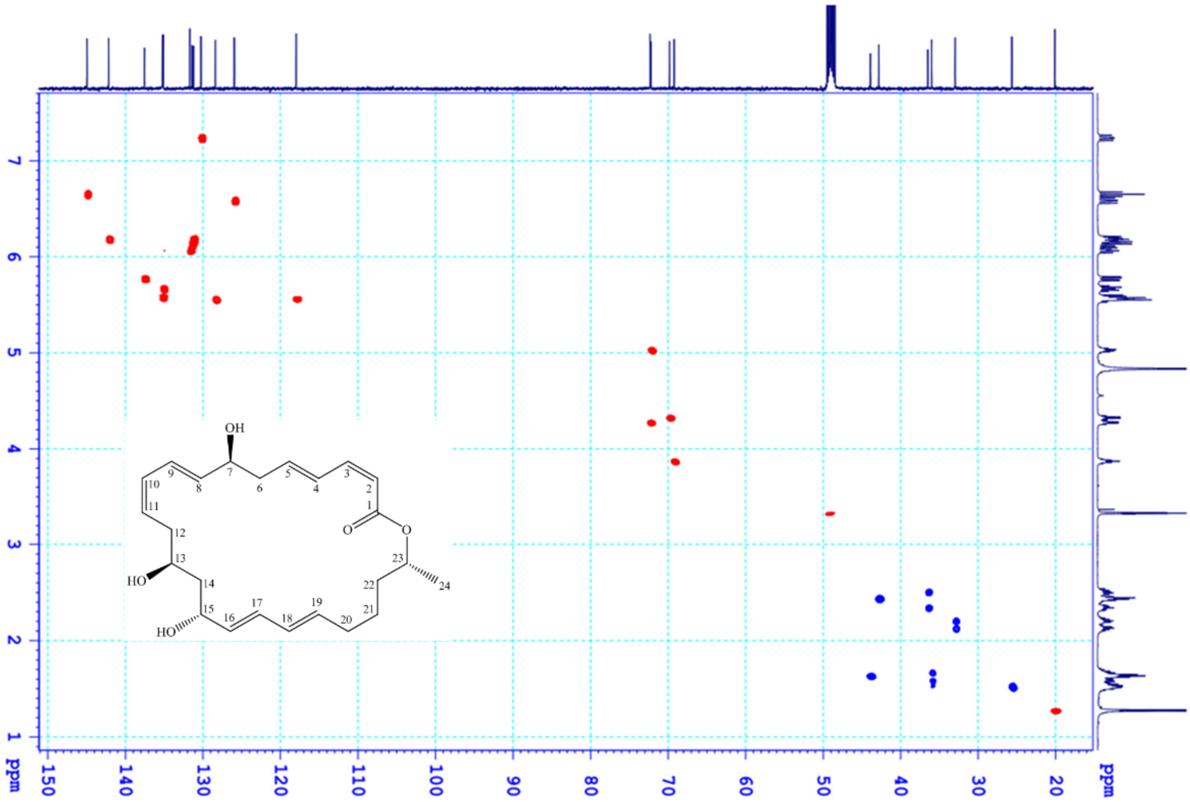

Figure S9. HSQC spectrum of macrolactin A (**2**)

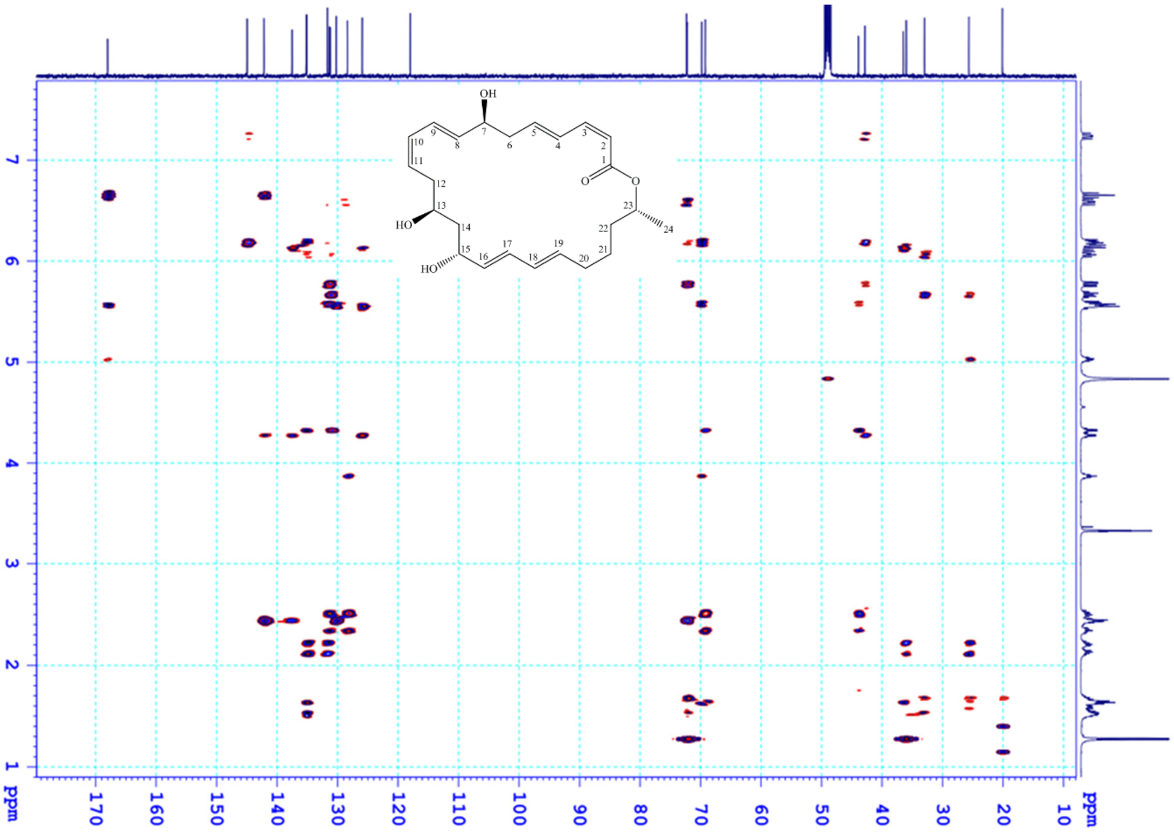

Figure S10. HMBC spectrum of macrolactin A (**2**)

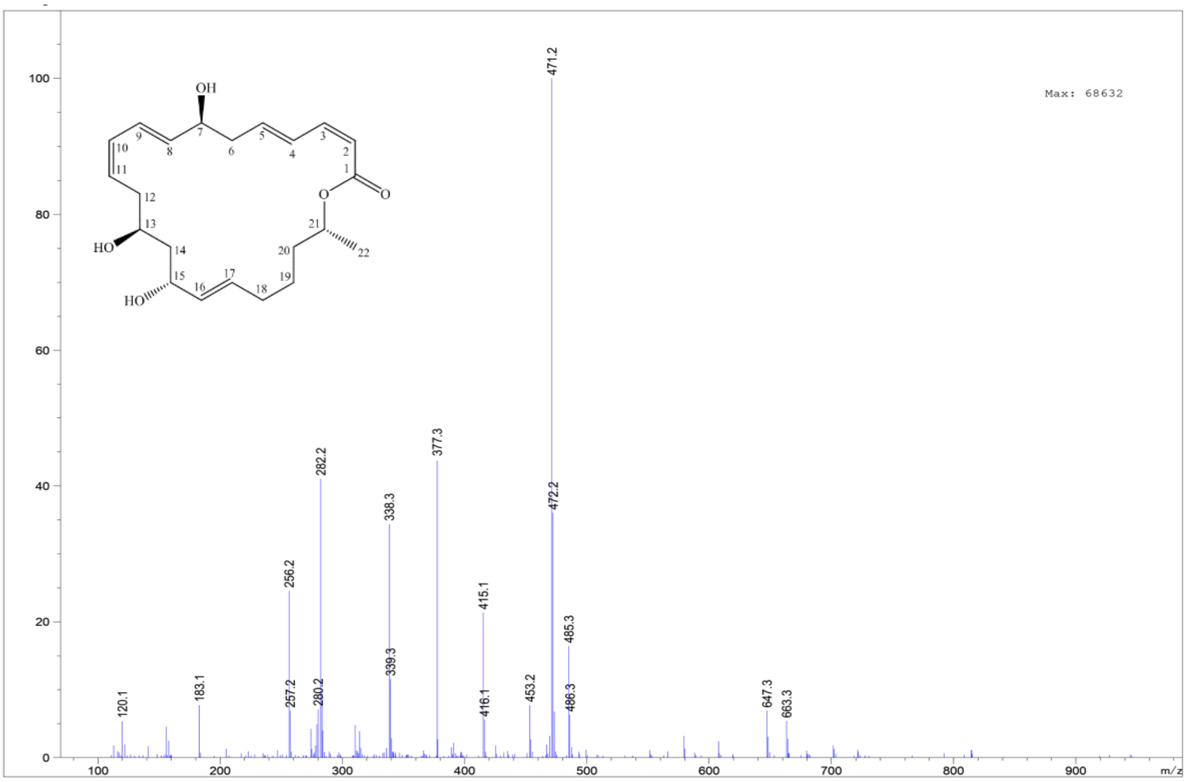

Figure S11. ESI-MS spectrum of macrolactin H (**3**)

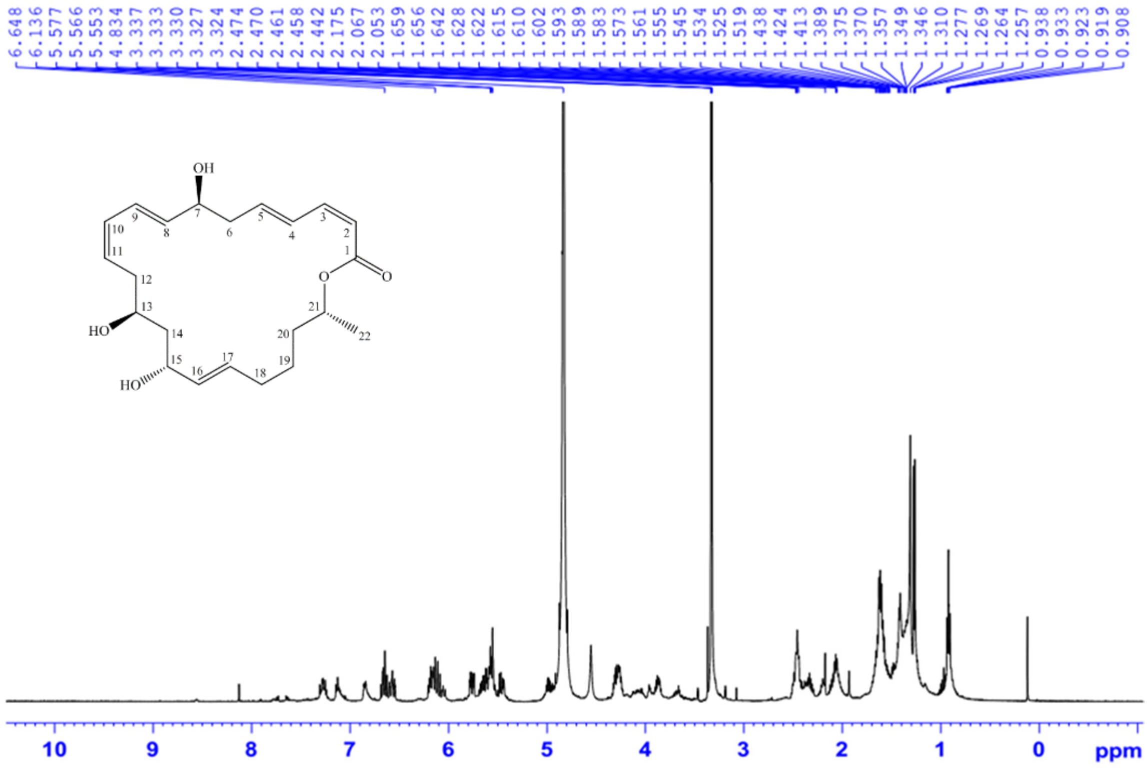

Figure S12. ^{1}H NMR spectrum of macrolactin H (**3**)

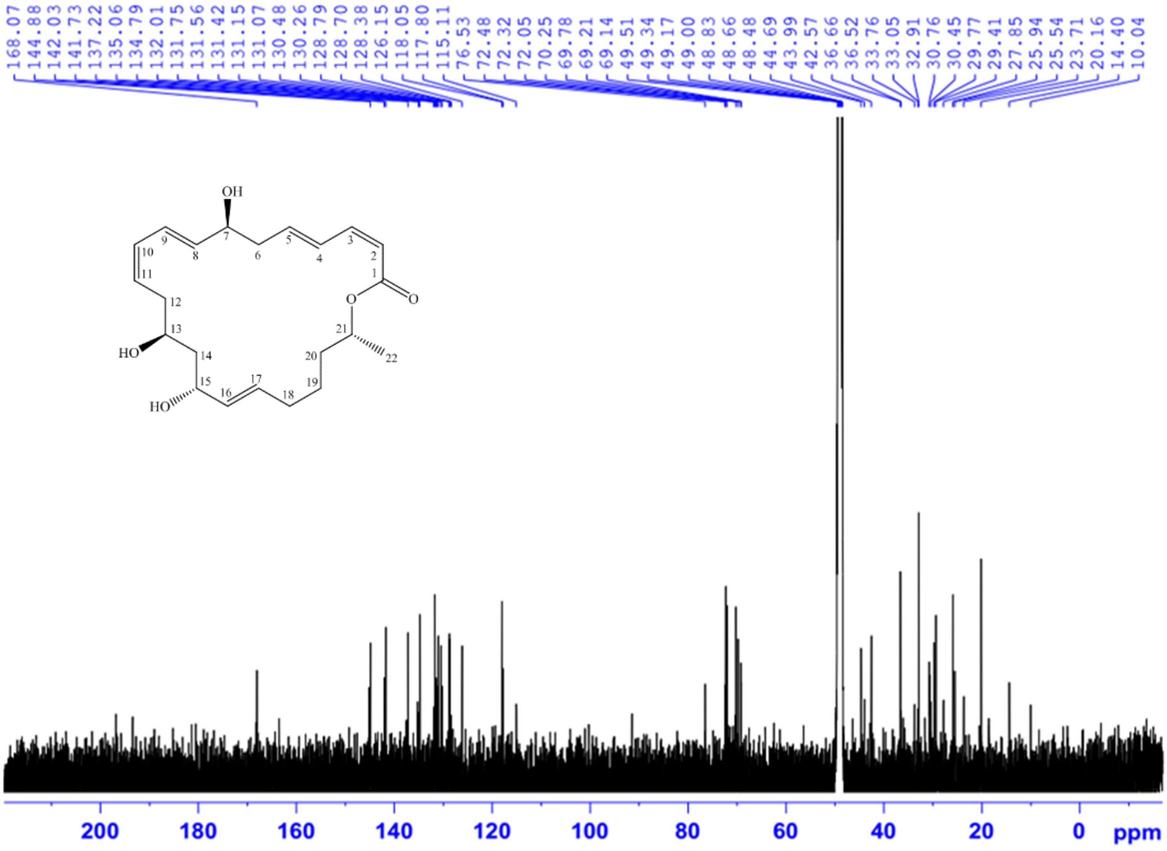

Figure S13. ^{13}C NMR spectrum of macrolactin H (**3**)

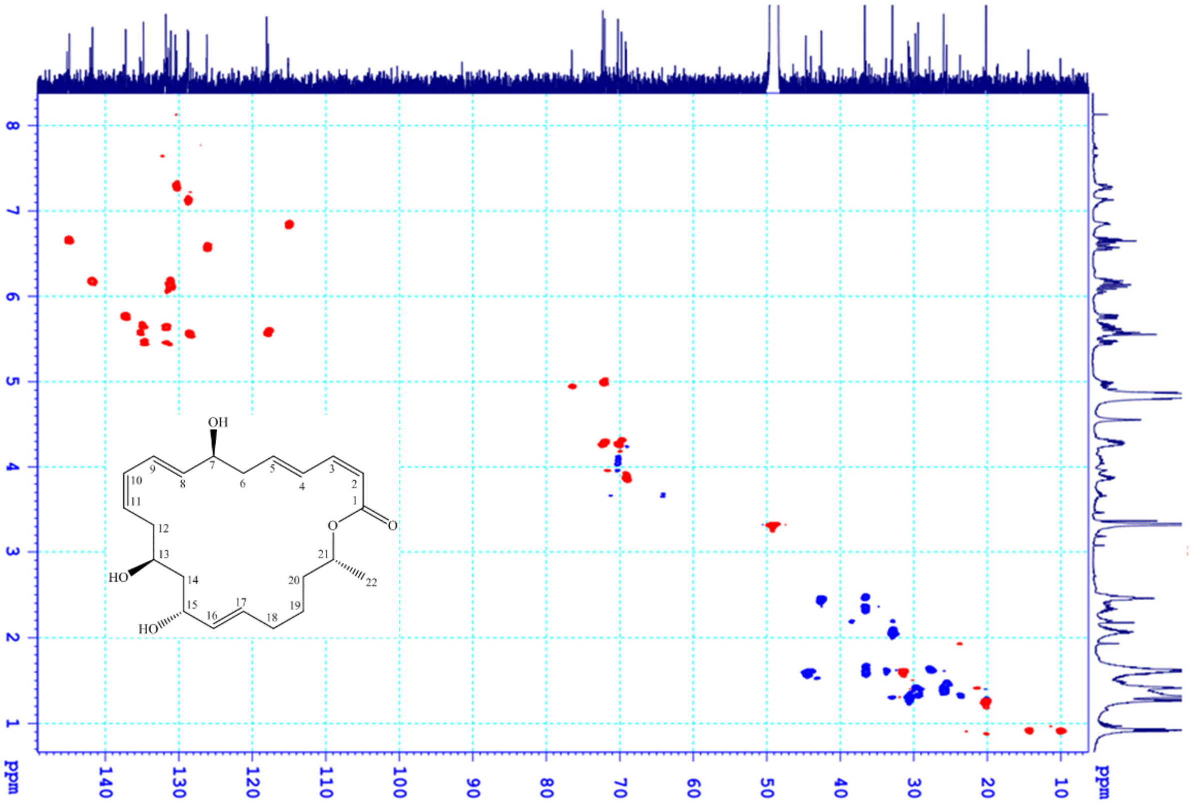

Figure S14. HSQC spectrum of macrolactin H (**3**)

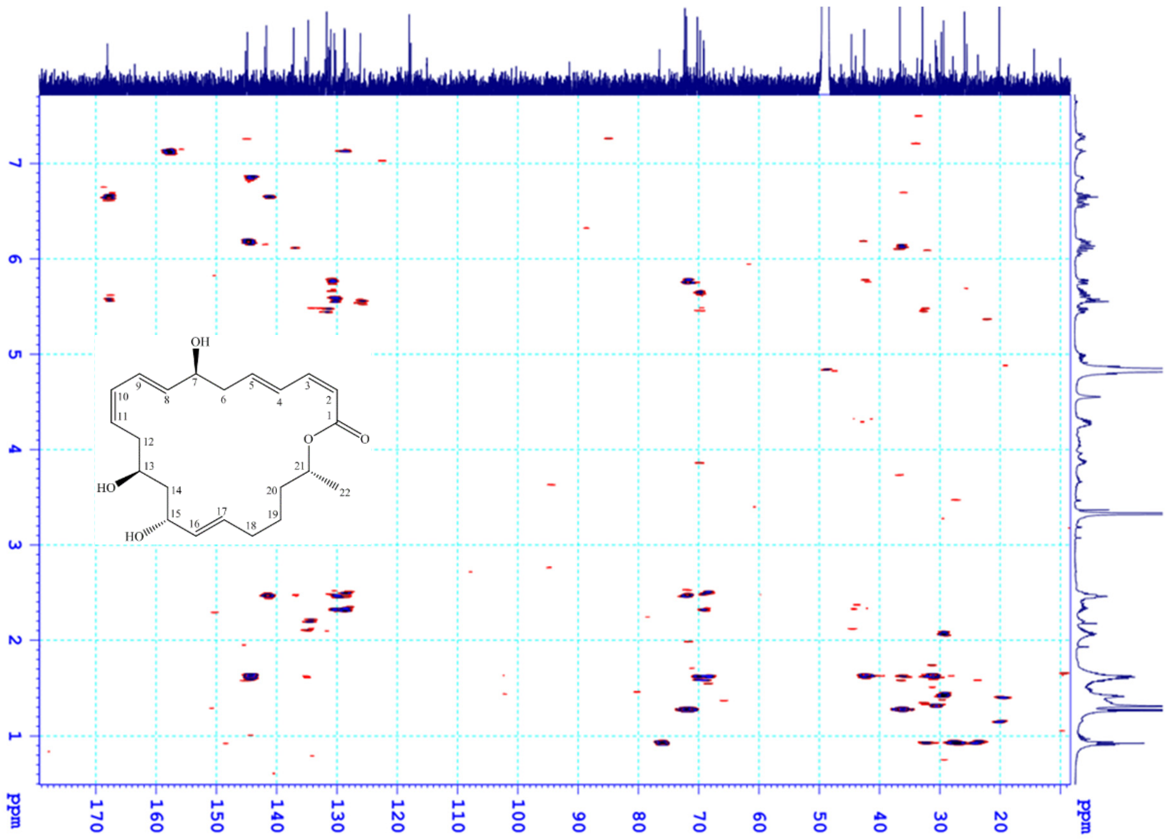

Figure S15. HMBC spectrum of macrolactin H (**3**)

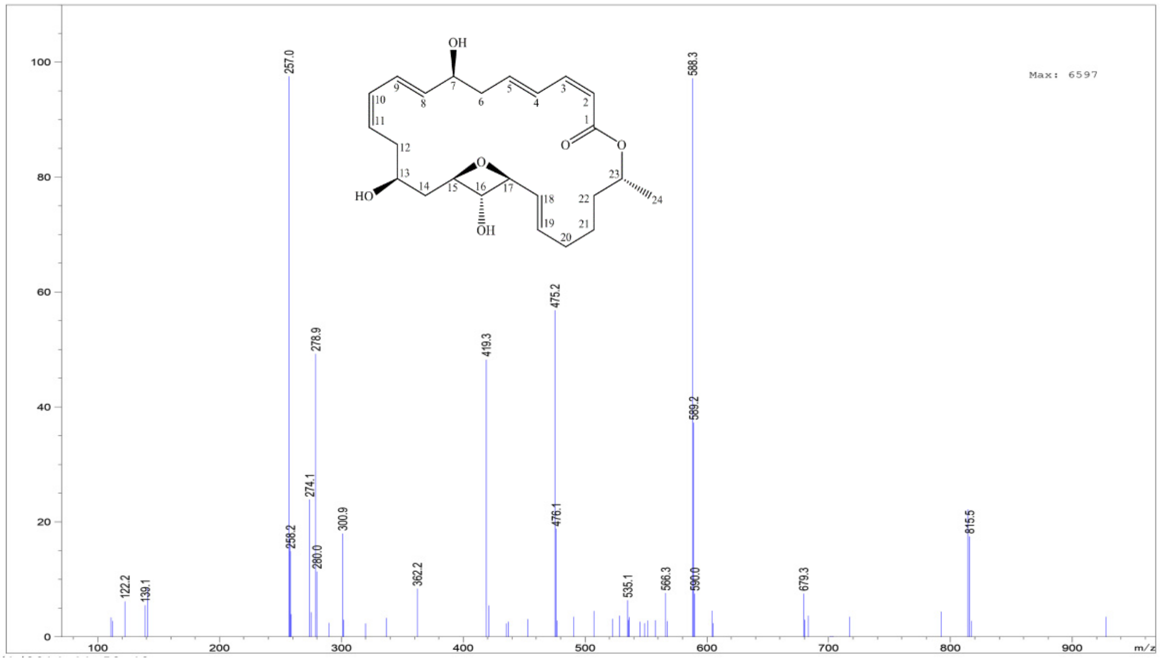

Figure S16. ESI-MS spectrum of 15,17-epoxy-16-hydroxy macrolactin A (**4**)

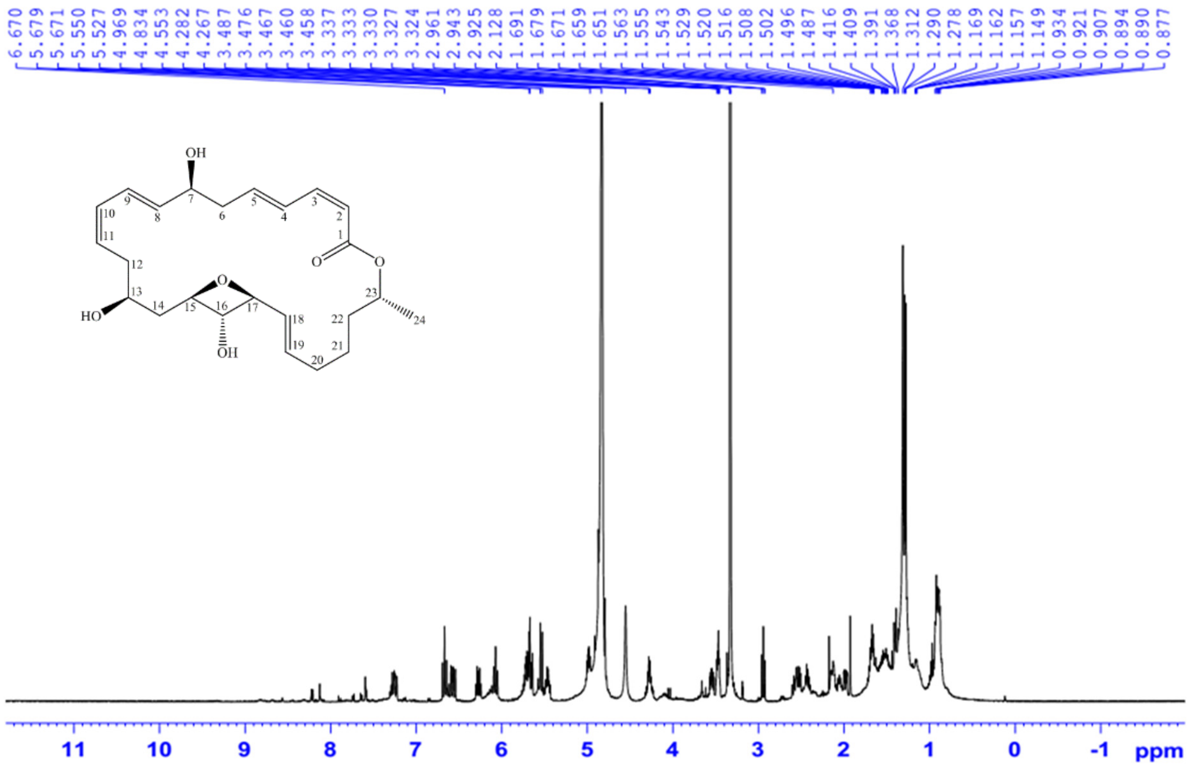

Figure S17. ^{1}H NMR spectrum of 15,17-epoxy-16-hydroxy macrolactin A (**4**)

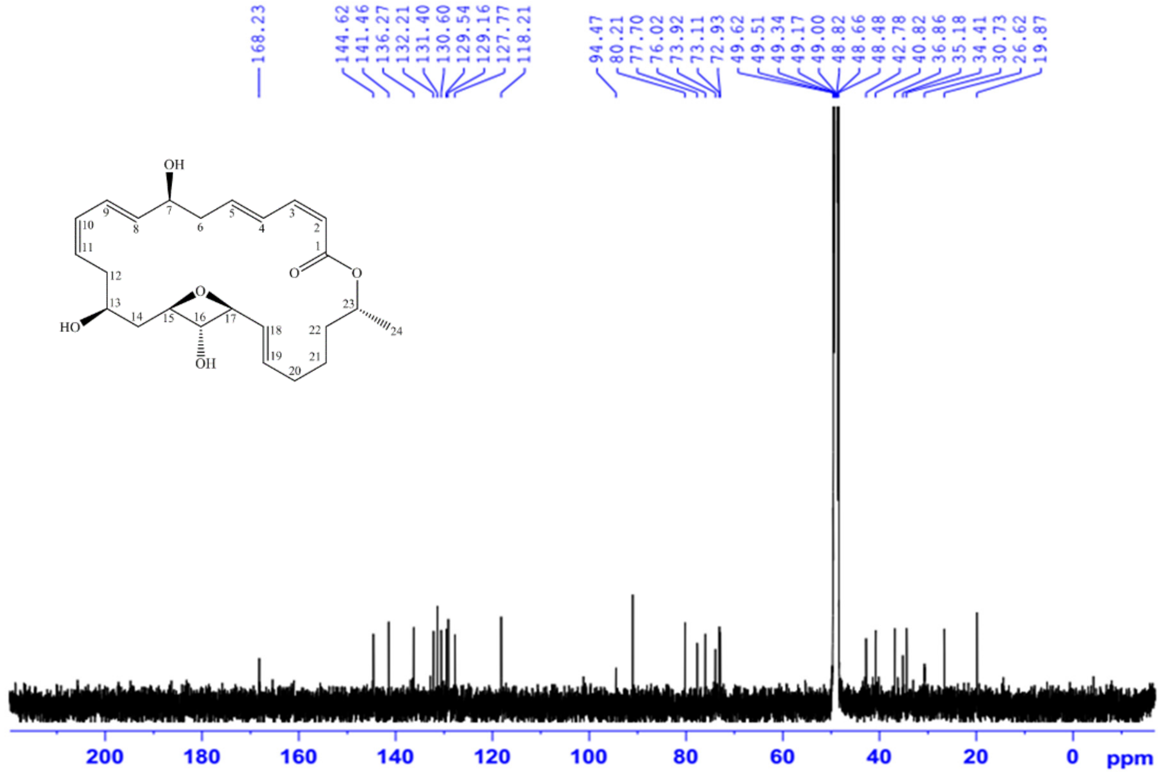

Figure S18. ^{13}C NMR spectrum of 15,17-epoxy-16-hydroxy macrolactin A (**4**)

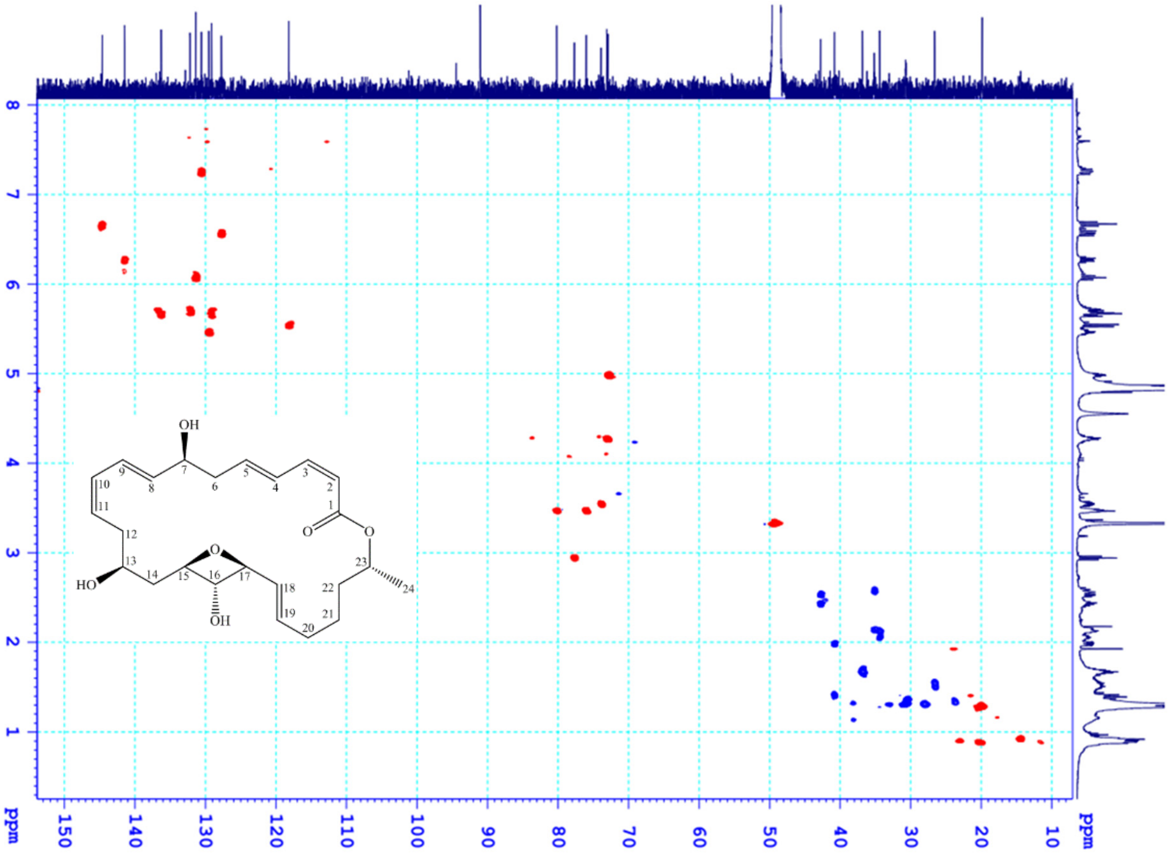

Figure S19. HSQC spectrum of 15,17-epoxy-16-hydroxy macrolactin A (**4**)

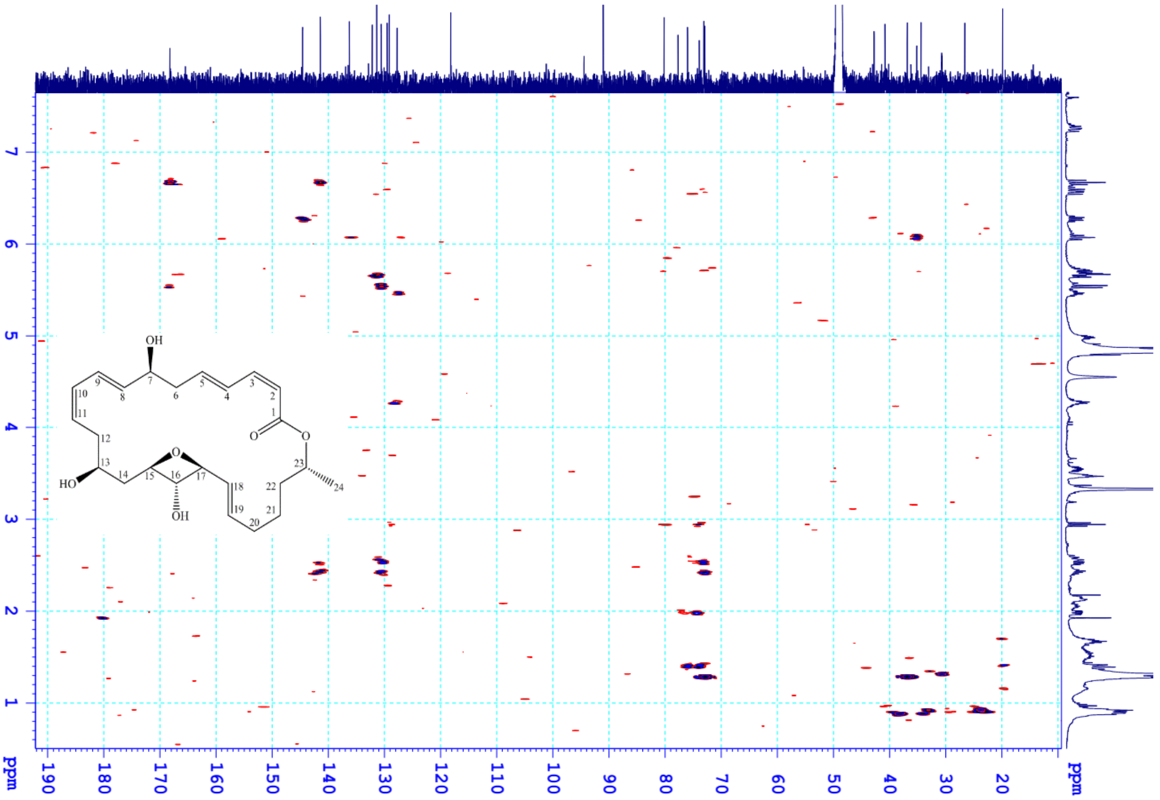

Figure S20. HMBC spectrum of 15,17-epoxy-16-hydroxy macrolactin A (**4**)

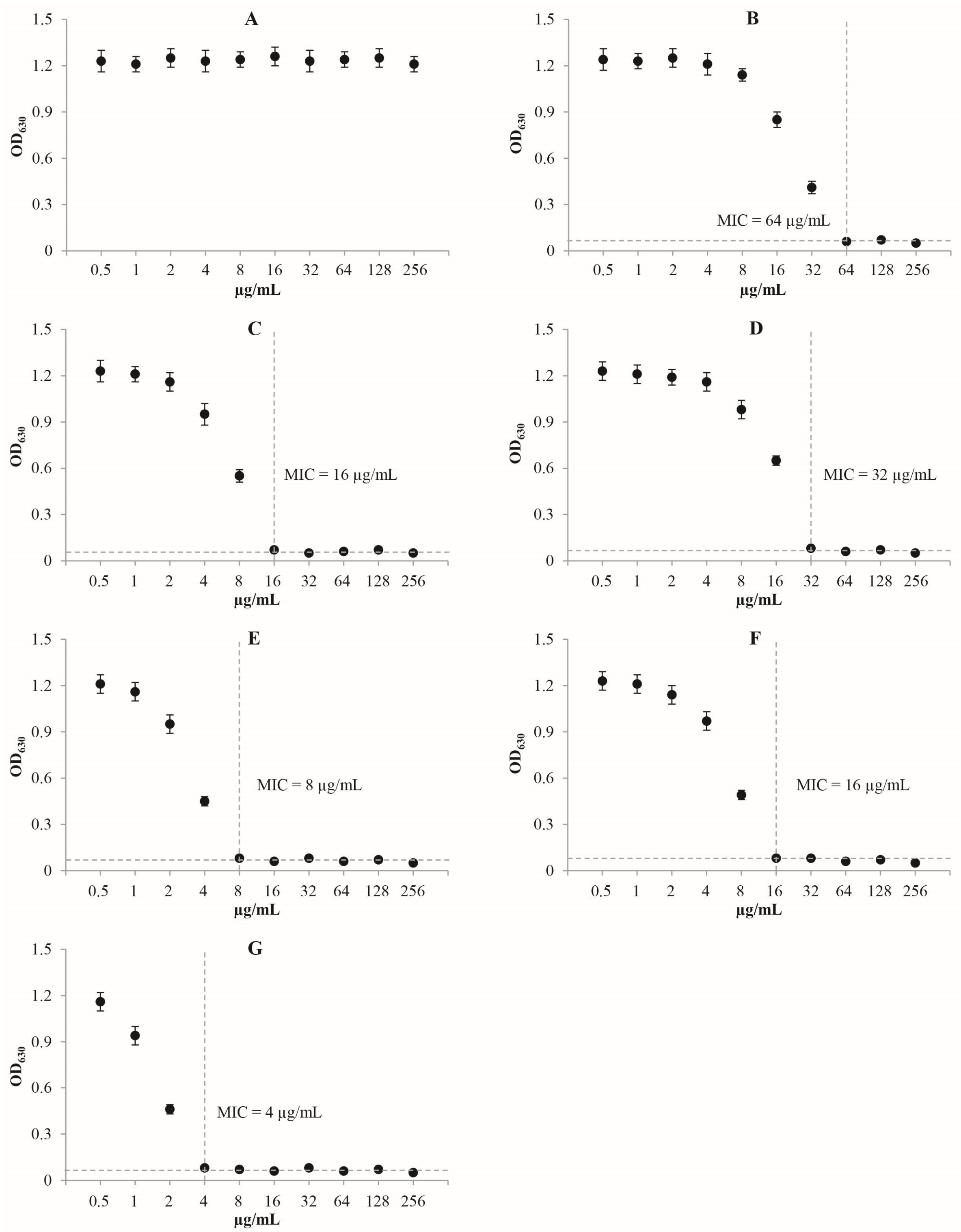

Figure S21. Effects of compounds Cyclo-(L-Pro-L-Tyr) (**A**), macrolactin A (**B**), macrolactin H (**C**), 15,17-epoxy-16-hydroxy macrolactin A (**D**), Ampicillin (**E**), Kanamycin (**F**) and Tetracycline (**G**) on the growth of *E. coli* ATCC 25922

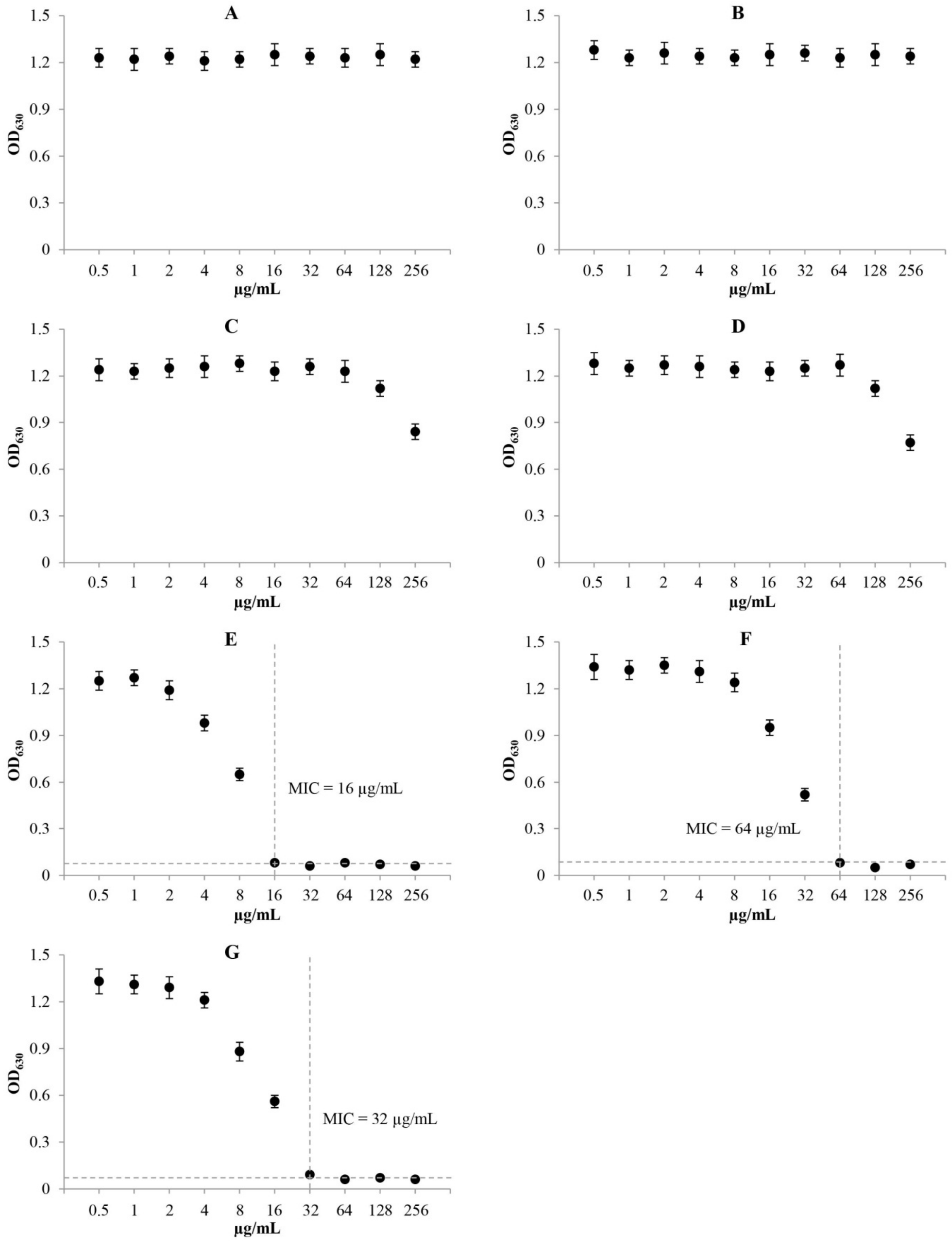

Figure S22. Effects of compounds Cyclo-(L-Pro-L-Tyr) (**A**), macrolactin A (**B**), macrolactin H (**C**), 15,17-epoxy-16-hydroxy macrolactin A (**D**), Ampicillin (**E**), Kanamycin (**F**) and Tetracycline (**G**) on the growth of *E. enterica* ATCC 13076

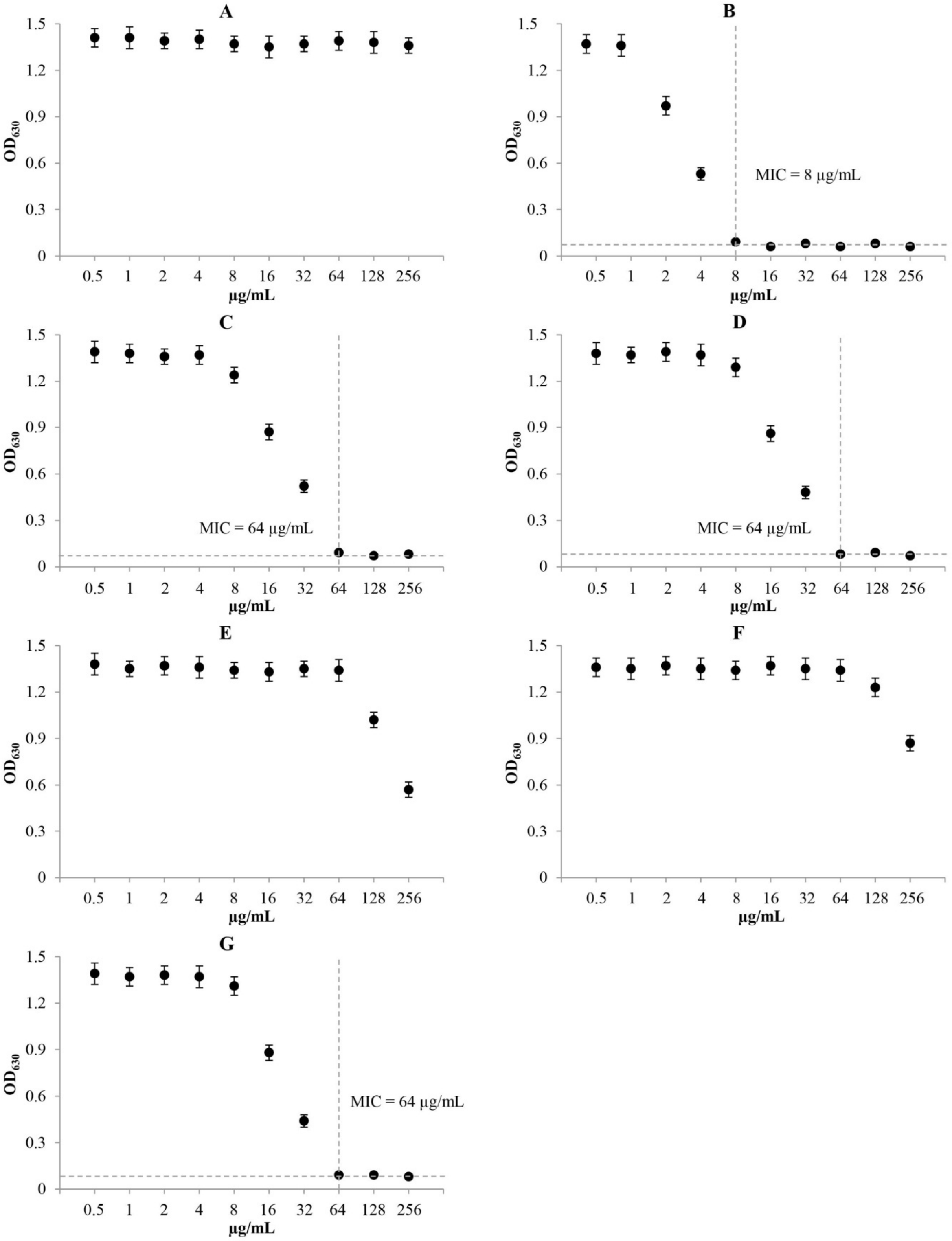

Figure S23. Effects of compounds Cyclo-(L-Pro-L-Tyr) (**A**), macrolactin A (**B**), macrolactin H (**C**), 15,17-epoxy-16-hydroxy macrolactin A (**D**), Ampicillin (**E**), Kanamycin (**F**) and Tetracycline (**G**) on the growth of *P. aeruginosa* ATCC 27853

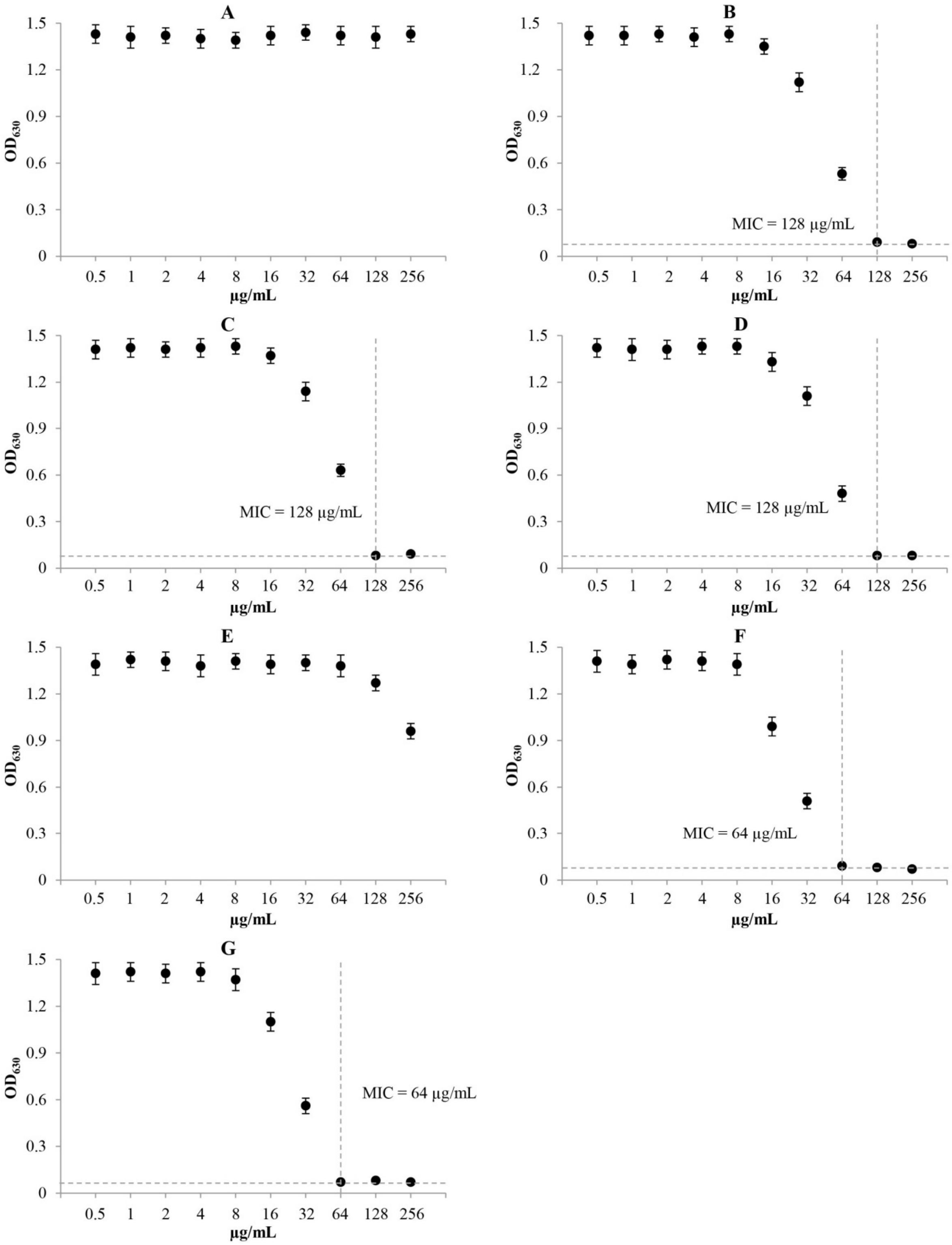

Figure S24. Effects of compounds Cyclo-(L-Pro-L-Tyr) (**A**), macrolactin A (**B**), macrolactin H (**C**), 15,17-epoxy-16-hydroxy macrolactin A (**D**), Ampicillin (**E**), Kanamycin (**F**) and Tetracycline (**G**) on the growth of *P. putida* MISR 71218

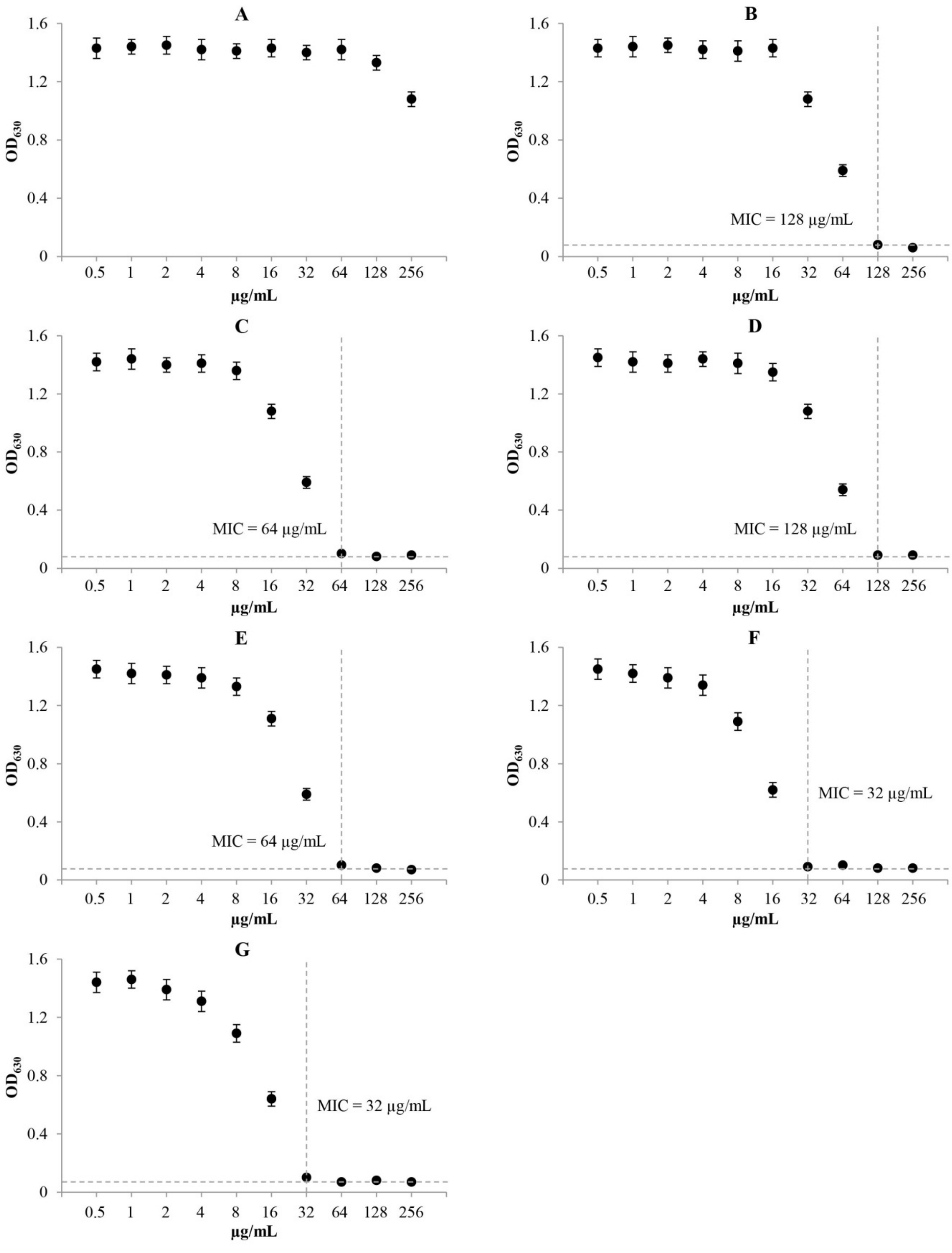

Figure S25. Effects of compounds Cyclo-(L-Pro-L-Tyr) (**A**), macrolactin A (**B**), macrolactin H (**C**), 15,17-epoxy-16-hydroxy macrolactin A (**D**), Ampicillin (**E**), Kanamycin (**F**) and Tetracycline (**G**) on the growth of *V. parahaemolyticus* MISR 21116

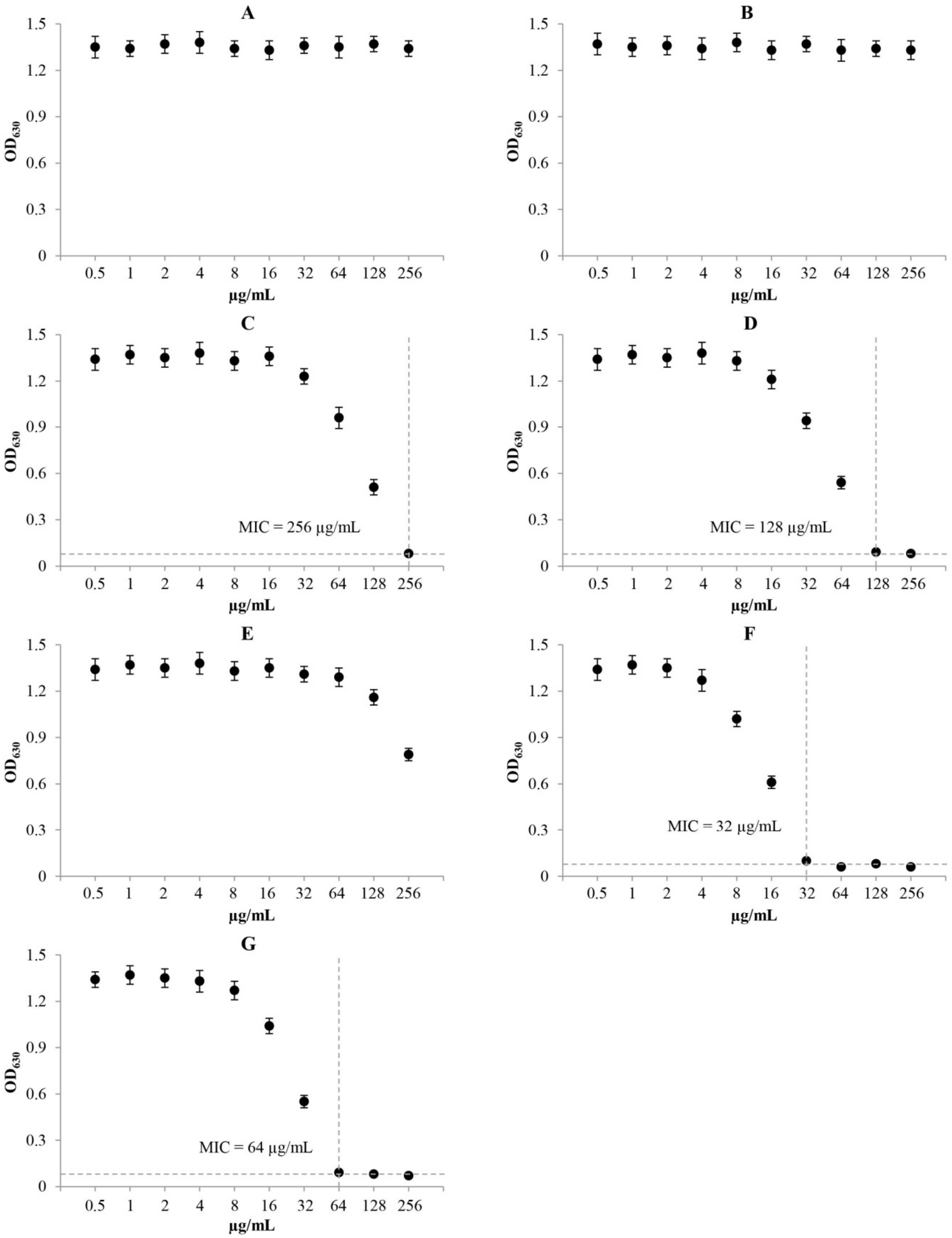

Figure S26. Effects of compounds Cyclo-(L-Pro-L-Tyr) (**A**), macrolactin A (**B**), macrolactin H (**C**), 15,17-epoxy-16-hydroxy macrolactin A (**D**), Ampicillin (**E**), Kanamycin (**F**) and Tetracycline (**G**) on the growth of *V. alginolyticus* MISR 30816

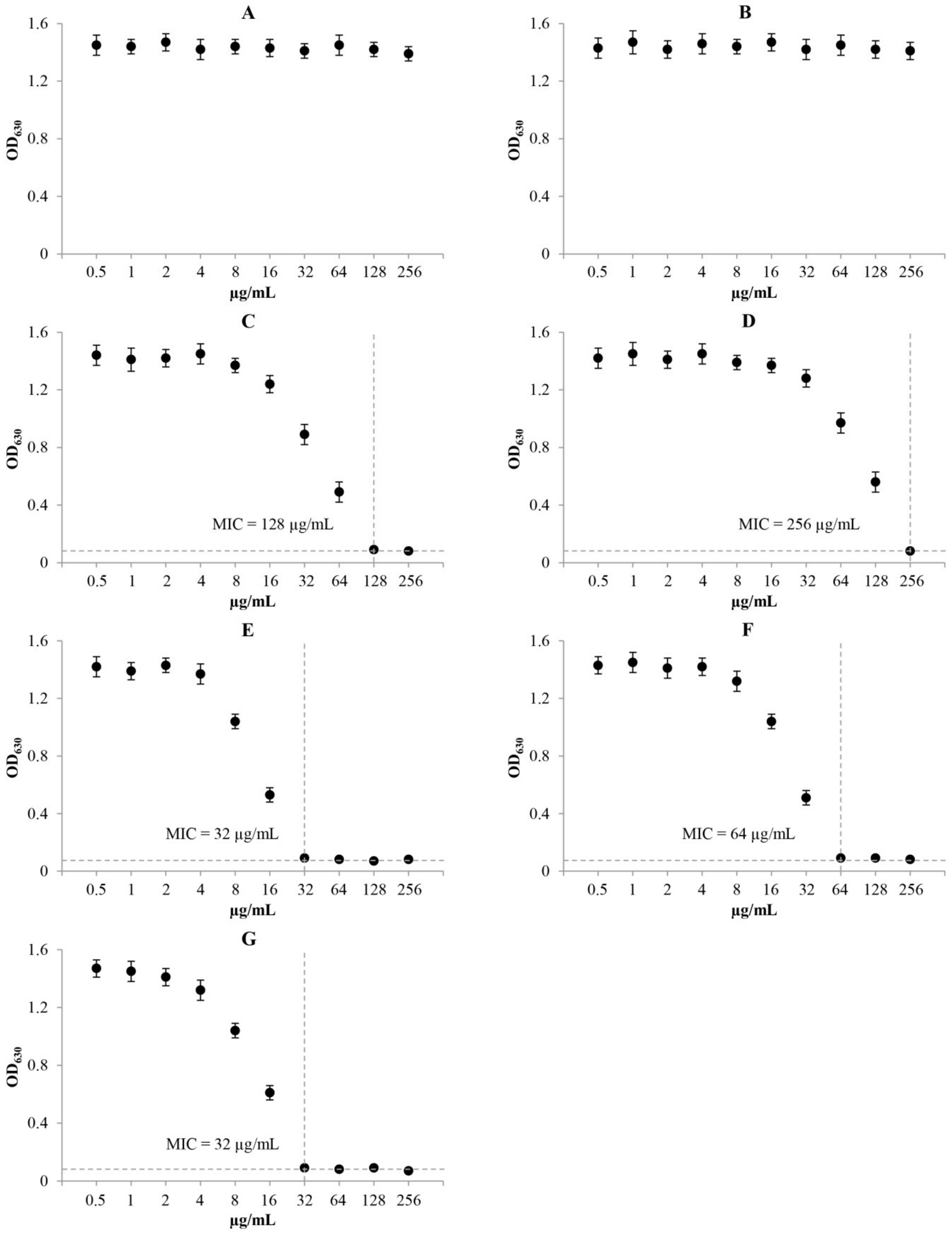

Figure S27. Effects of compounds Cyclo-(L-Pro-L-Tyr) (**A**), macrolactin A (**B**), macrolactin H (**C**), 15,17-epoxy-16-hydroxy macrolactin A (**D**), Ampicillin (**E**), Kanamycin (**F**) and Tetracycline (**G**) on the growth of *V. vulnificus* MISR 20716

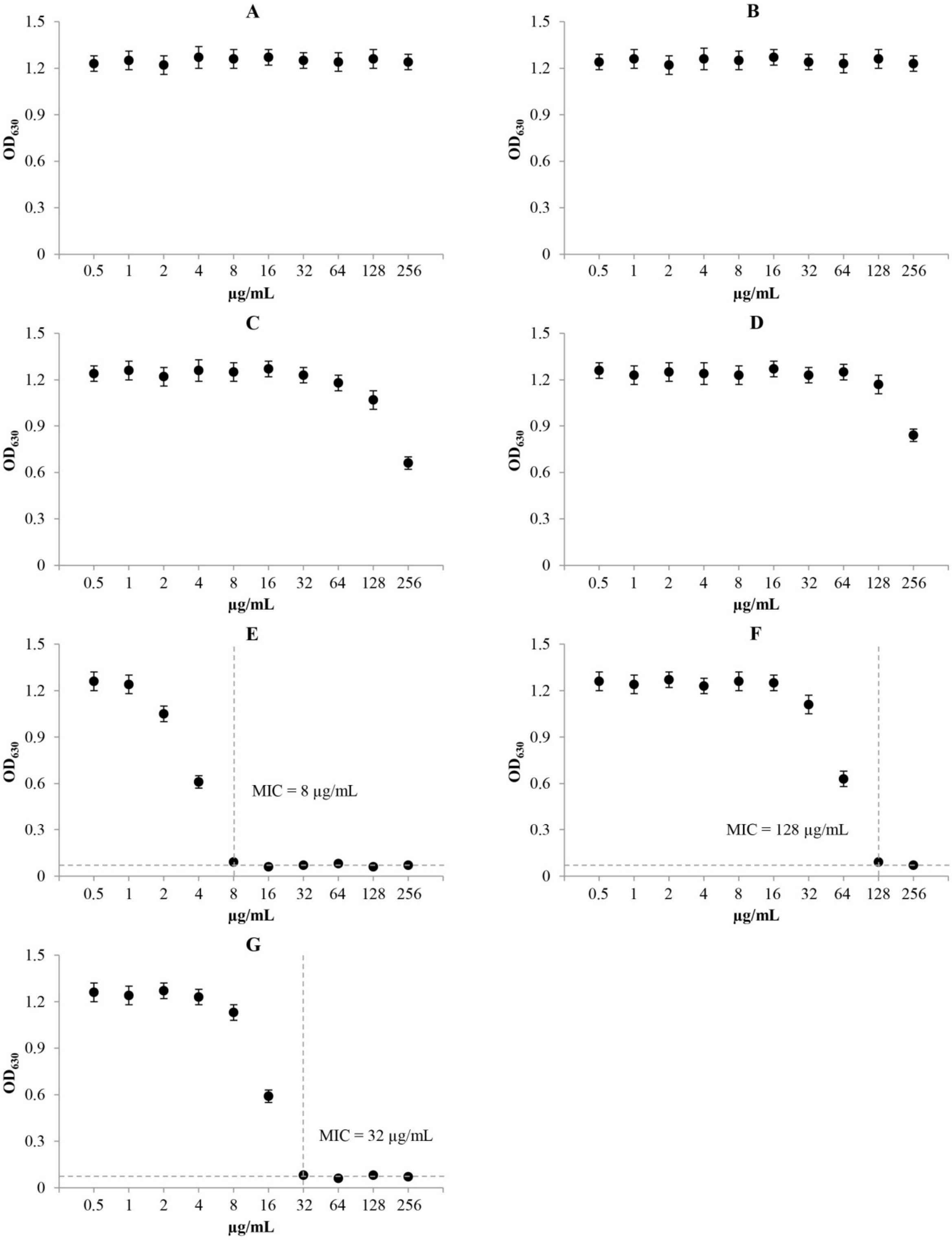

Figure S28. Effects of compounds Cyclo-(L-Pro-L-Tyr) (**A**), macrolactin A (**B**), macrolactin H (**C**), 15,17-epoxy-16-hydroxy macrolactin A (**D**), Ampicillin (**E**), Kanamycin (**F**) and Tetracycline (**G**) on the growth of *E. faecalis* ATCC 29212

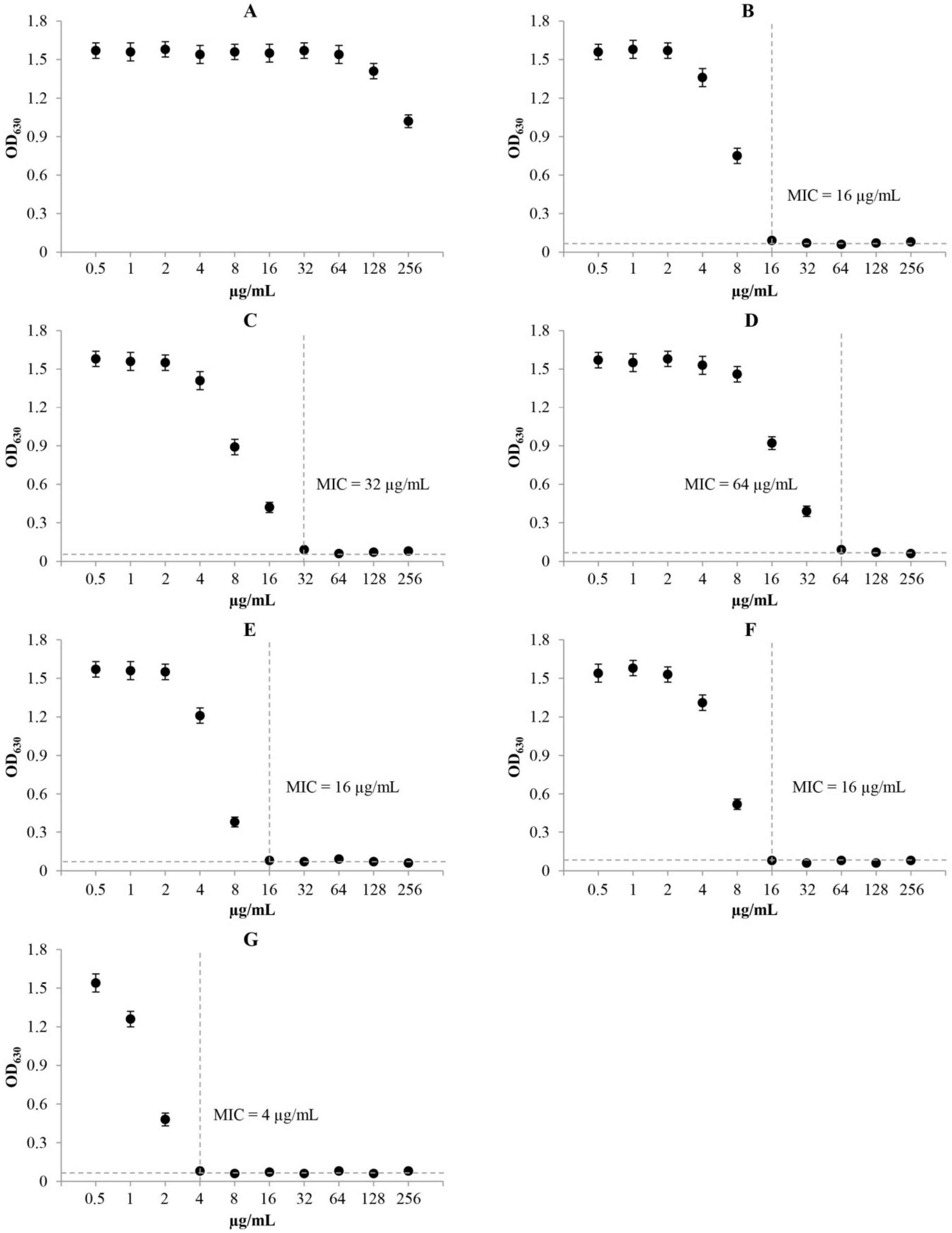

Figure S29. Effects of compounds Cyclo-(L-Pro-L-Tyr) (**A**), macrolactin A (**B**), macrolactin H (**C**), 15,17-epoxy-16-hydroxy macrolactin A (**D**), Ampicillin (**E**), Kanamycin (**F**) and Tetracycline (**G**) on the growth of *S. aureus* ATCC 25923

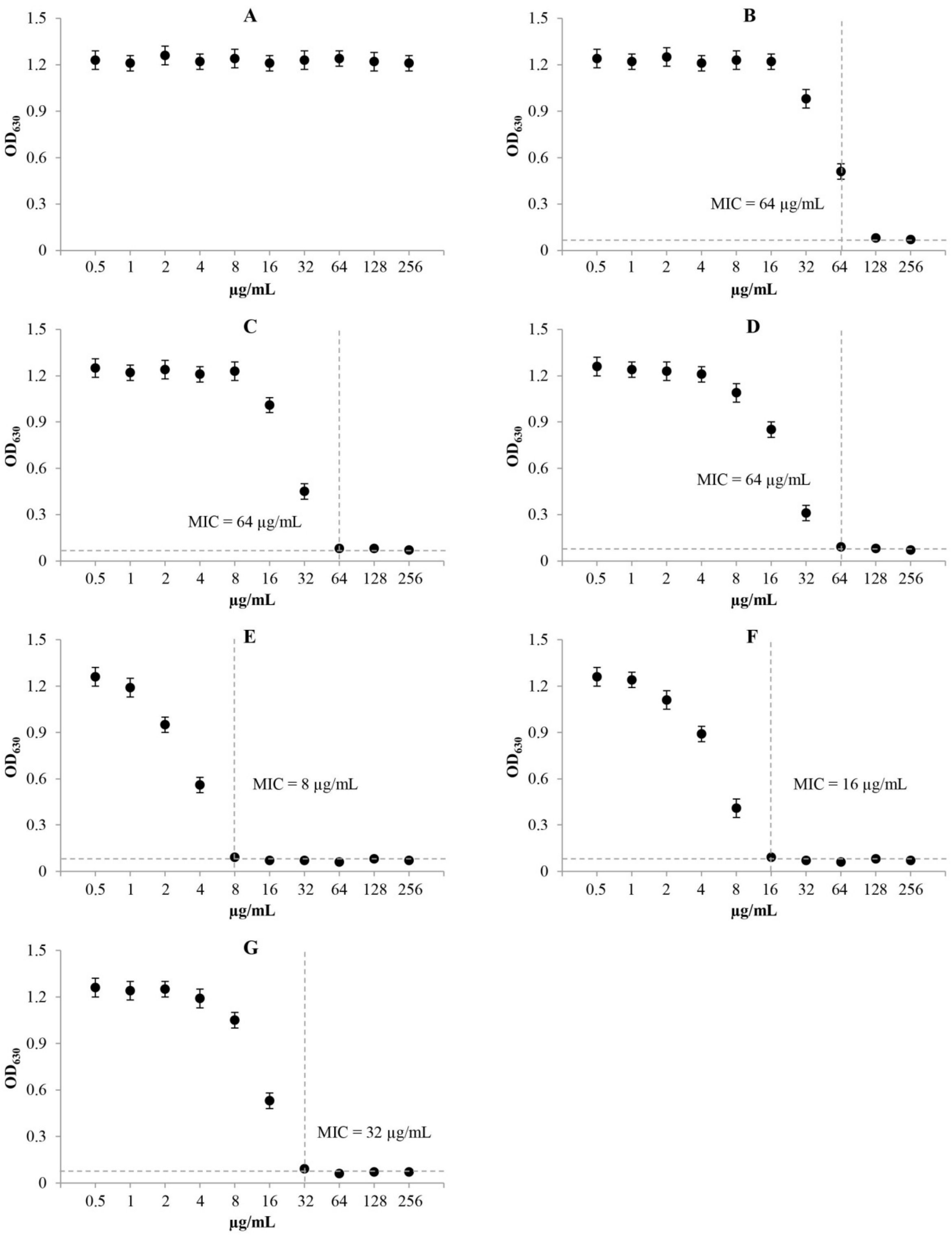

Figure S30. Effects of compounds Cyclo-(L-Pro-L-Tyr) (**A**), macrolactin A (**B**), macrolactin H (**C**), 15,17-epoxy-16-hydroxy macrolactin A (**D**), Ampicillin (**E**), Kanamycin (**F**) and Tetracycline (**G**) on the growth of *B. subtilis* ATCC 6633

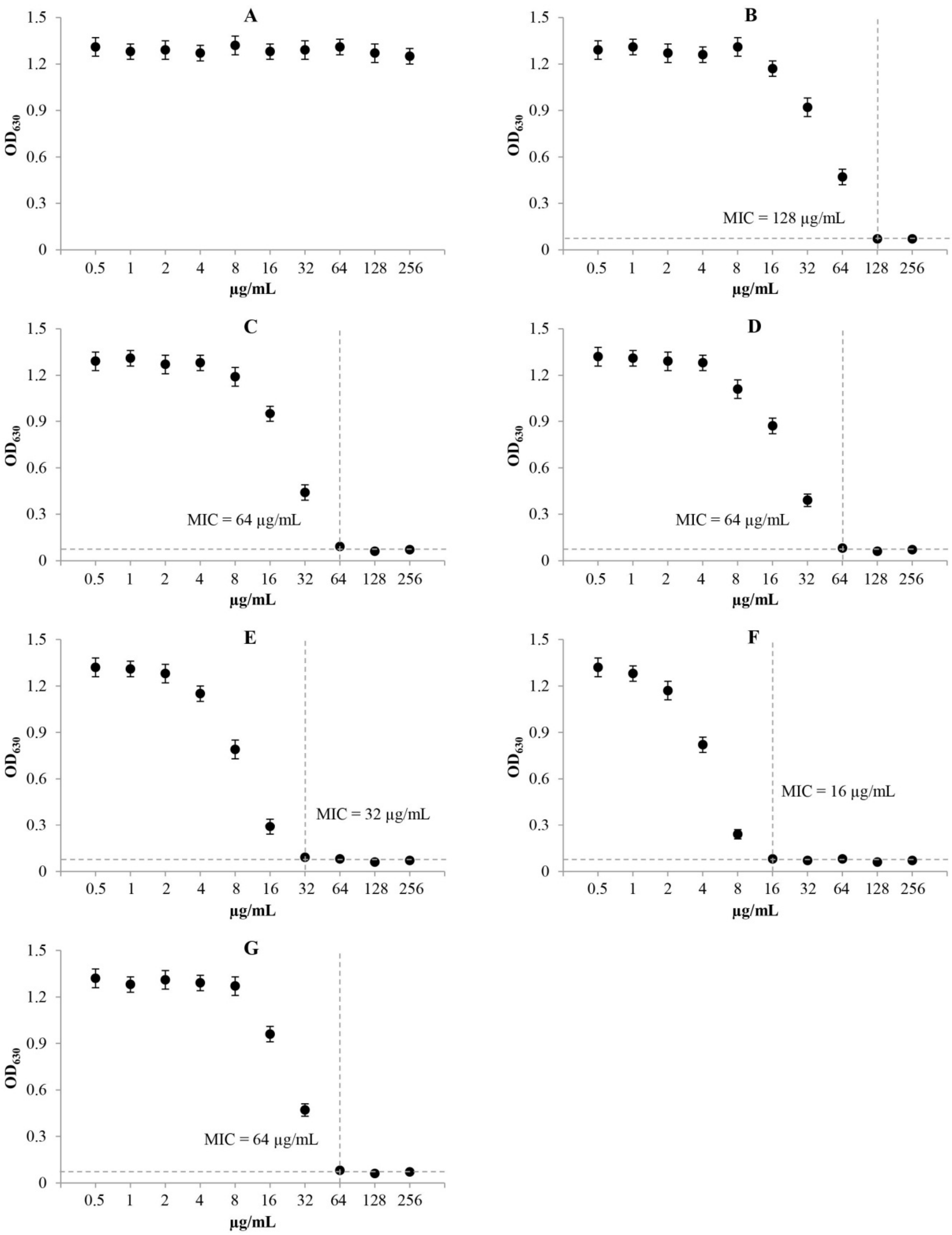

Figure S31. Effects of compounds Cyclo-(L-Pro-L-Tyr) (**A**), macrolactin A (**B**), macrolactin H (**C**), 15,17-epoxy-16-hydroxy macrolactin A (**D**), Ampicillin (**E**), Kanamycin (**F**) and Tetracycline (**G**) on the growth of *B. cereus* MISR 12818

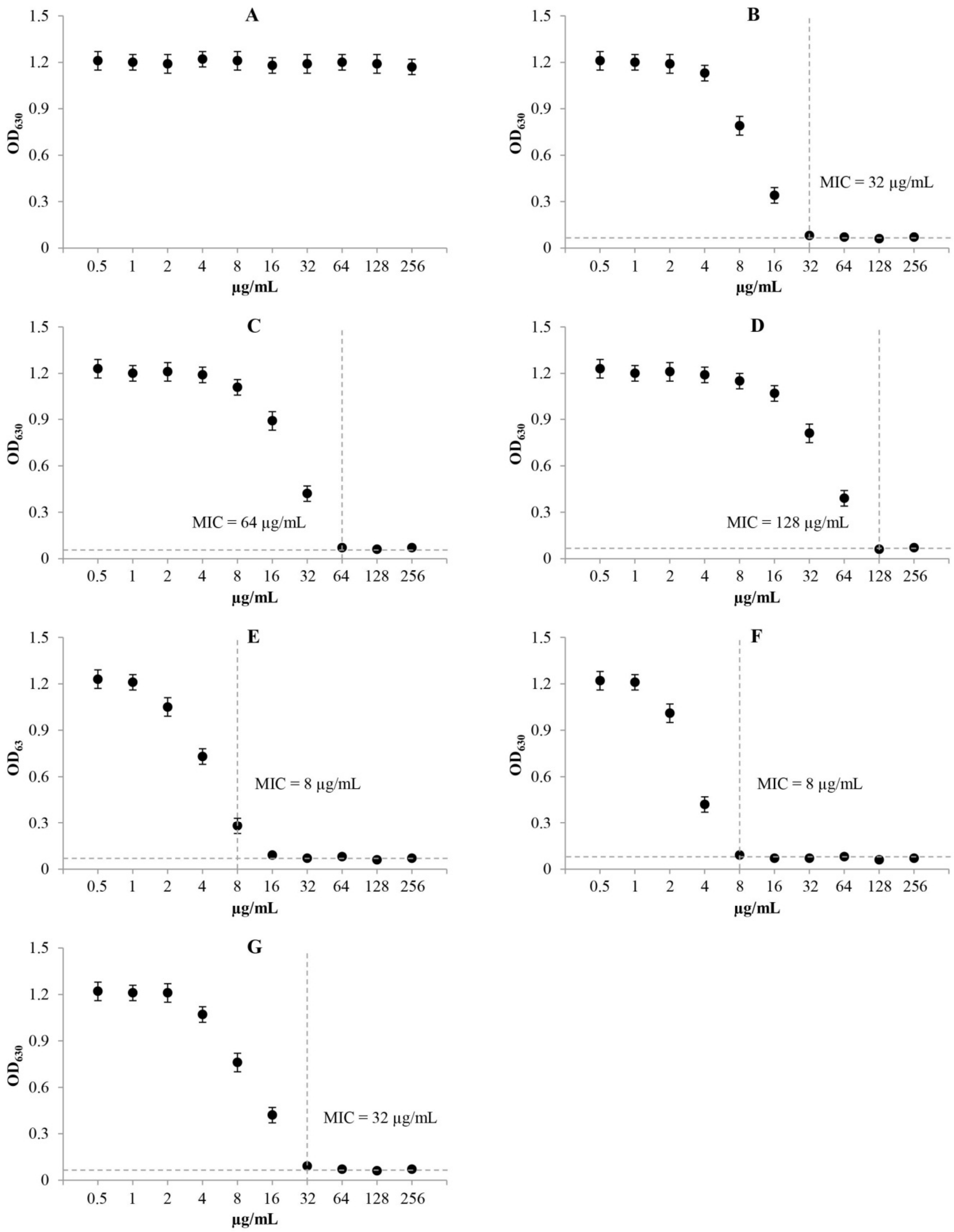

Figure S32. Effects of compounds Cyclo-(L-Pro-L-Tyr) (**A**), macrolactin A (**B**), macrolactin H (**C**), 15,17-epoxy-16-hydroxy macrolactin A (**D**), Ampicillin (**E**), Kanamycin (**F**) and Tetracycline (**G**) on the growth of *Rhodococcus* sp. MISR 16518

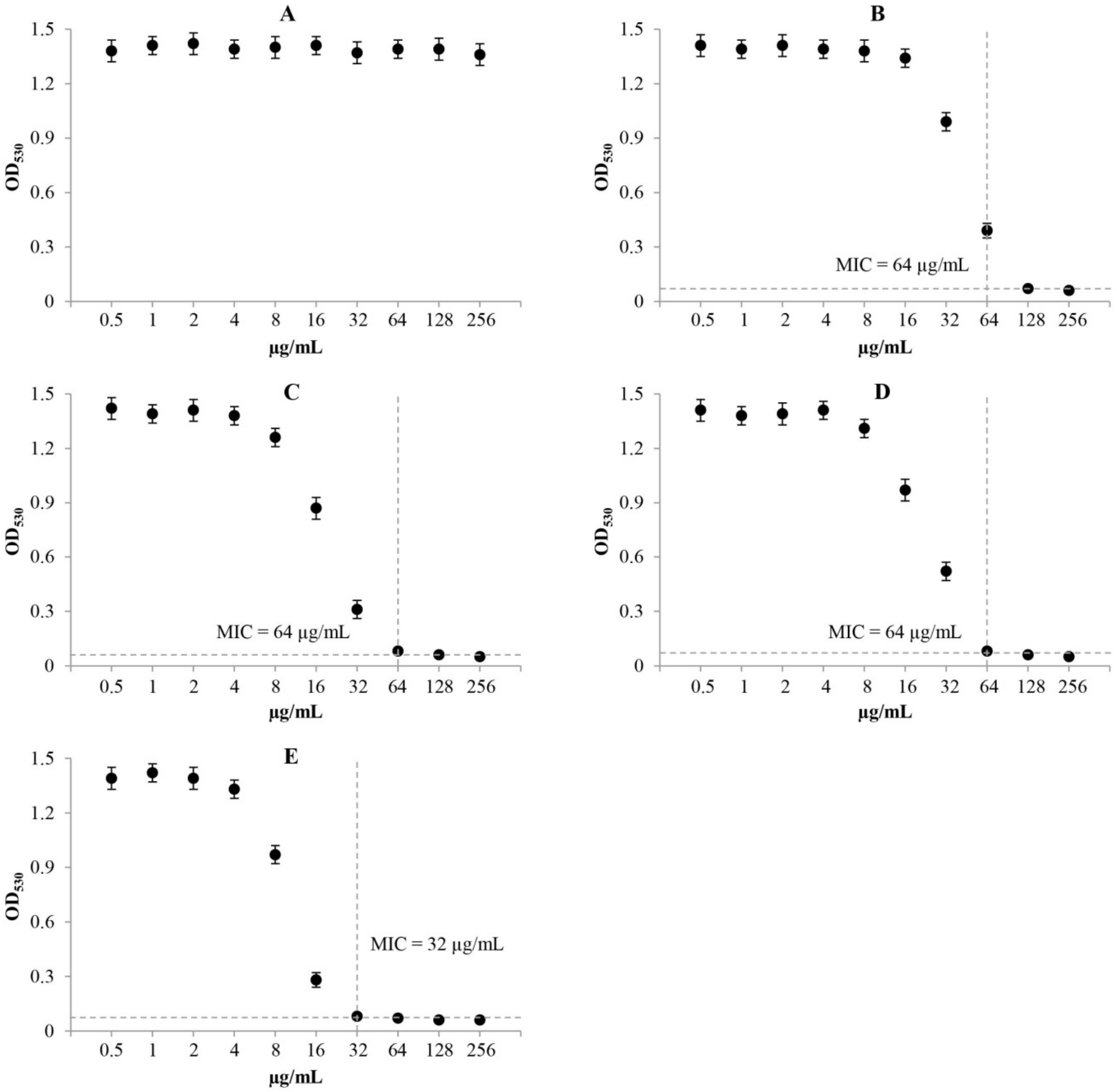

Figure S33. Effects of compounds Cyclo-(L-Pro-L-Tyr) (**A**), macrolactin A (**B**), macrolactin H (**C**), 15,17-epoxy-16-hydroxy macrolactin A (**D**), Miconazole (**E**) on the growth of *C. albicans* ATCC 10231

1. Quyen, T. V.; Ngan, T. B.; Huong, D. T. M.; Kondracki, M. L. B.; Longeon, A.; Murphy, B.; Minh, C. V.; Cuong, P. V., Secondary metabolites from Micromonospora ectrinospora G017. *Vietnam Journal of Chemistry* **2015,** 53, (2e), 146-149.
2. Lee, S.-J.; Cho, J.-Y.; Cho, J.-I.; Moon, J.-H.; Park, K. D.; Lee, Y. J.; Park, K.-H., Isolation and Characterization of Antimicrobial Substance Macrolactin A Produced from Bacillus amyloliquefaciens CHO104 Isolated from Soil. *Journal of Microbiology and Biotechnology* **2004,** 14, (3), 525-531.
3. Nagao, T.; Adachi, K.; Sakai, M.; Nishijima, M.; Sano, H., Novel Macrolactins as antibiotic lactones from a marine bacterium. *The Journal of antibiotics* **2001,** 54, (4), 333-339.
4. Mondol, M. A. M.; Tareq, F. S.; Kim, J. H.; Lee, M. a.; Lee, H.-S.; Lee, Y.-J.; Lee, J. S.; Shin, H. J., Cyclic Ether-Containing Macrolactins, Antimicrobial 24-Membered Isomeric Macrolactones from a Marine Bacillus sp. *Journal of Natural Products* **2011,** 74, (12), 2582-2587.
